# Structure-Guided Semisynthesis of Blasticidin S–Amicetin Chimeras as Selective Ribosome Inhibitors

**DOI:** 10.1021/jacs.5c13979

**Published:** 2026-01-12

**Authors:** Cole Gannett, Kateland Tiller, Somaia Abdelmegeed, Micah Hoernig, Ahmed A. Abouelkhair, Mohamed N. Seleem, James Weger-Lucarelli, Anne M. Brown, Andrew N. Lowell

**Affiliations:** † Department of Chemistry, 1757 Virginia Polytechnic Institute and State University (Virginia Tech), Blacksburg, Virginia 24061, United States; ‡ Center for Emerging, Zoonotic, and Arthropod-borne Pathogens, Virginia Tech, Blacksburg, Virginia 24061, United States; § Virginia Tech Center for Drug Discovery, Virginia Tech, Blacksburg, Virginia 24061, United States; ∥ Department of Biomedical Sciences and Pathobiology, Virginia Tech, VA-MD Regional College of Veterinary Medicine, Blacksburg, Virginia 24061, United States; ⊥ Department of Biochemistry, Virginia Tech, Blacksburg, Virginia 24061, United States; # Center for One Health Research, Virginia Tech, Blacksburg, Virginia 24061, United States; ∇ Research and Informatics, University Libraries, Virginia Tech, Blacksburg, Virginia 24061, United States; ○ Faculty of Health Sciences, Virginia Polytechnic Institute and State University (Virginia Tech), Blacksburg, Virginia 24061, United States

## Abstract

Peptidyl nucleosides are broad-acting inhibitors, but their dense functionality and complex reactivity have historically limited the modification of these scaffolds. Guided by structural overlays and molecular modeling, we designed blasticidin S-amicetin chimeras to exploit a bacterial-specific pocket of the ribosomal PTC while reducing eukaryotic ribosome engagement. To test this hypothesis, we developed a semisynthetic route enabling sequential C6′ derivatization and C4 amine coupling on the blasticidin S scaffold, facilitated by counterion exchange to prevent side reactions. This approach furnished four C6′ classes (acid, methyl ester, primary amide, phenethyl amide), each diversified at C4 with *para*-aminobenzoate motifs, delivering densely functionalized chimeras in as few as four steps and up to 38% yield. Across the series, antibacterial potency was retained while mammalian cytotoxicity dropped sharply, with selectivity indices approaching >50 and cytotoxicity values >256 μg/mL for the phenethyl amide series. Comparison of resolved ribosome structures supplemented by modeling rationalizes the observed selectivity gains as engagement of a termination-compatible bacterial pocket that is disfavored during eukaryotic elongation. These results demonstrate how structure-guided semisynthesis can transform a challenging natural product into selective translation inhibitors and establish a practical framework for diversifying chemically complex scaffolds.

## Graphical abstract

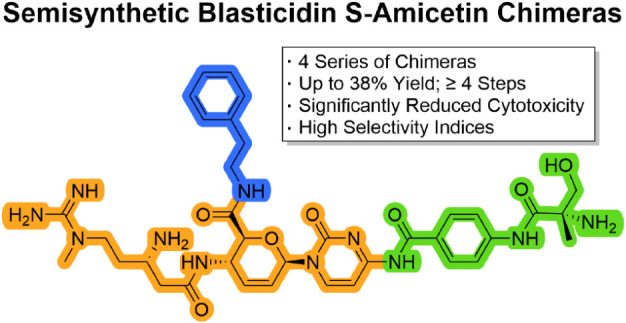

## Introduction

Natural products remain indispensable tools for probing and modulating biological machinery, yet their structural complexity often precludes systematic derivatization. Peptidyl nucleosides^1^ are a prime example with multiple reactive functional groups, poor solubility, and acid/base sensitivity creating reactivity issues that resist conventional strategies. This synthetic intractability limits exploration of structure–activity space, constraining both mechanistic investigations and the optimization of promising hits.

Blasticidin S (**1**, Figure 1) is a peptidyl nucleoside that inhibits protein synthesis in both bacterial and eukaryotic ribosomes, acting as a potent cytotoxin with little intrinsic selectivity.^2,3^ In contrast, the related nucleoside antibiotic amicetin (**5**)^4^ achieves greater bacterial selectivity, presumably by means of a *para*-aminobenzoate (PABA) arm that extends into an adjacent pocket near the ribosomal peptidyltransferase center (PTC).^5^ Overlays of resolved ribosome structures revealed that blasticidin S (PDB IDs: 4v9q,^3^ 6b4v^6^ 4u56,^7^ 7nwi,^8^ 7q0r)^9^ and amicetin (6czr)^10^ share a common cytosine base interaction in a pocket partially bounded by bacterial proteins L16 ^11^ and L27 ^12^ or eukaryotic protein L10.^13^ Structural investigation suggested that appending a PABA extension to blasticidin S could retain productive binding in bacterial termination complexes^3,6^ where this pocket is accessible, while creating a steric clash during eukaryotic elongation,^8^ thereby reducing off-target inhibition (Figure 2).

**Figure 1:**
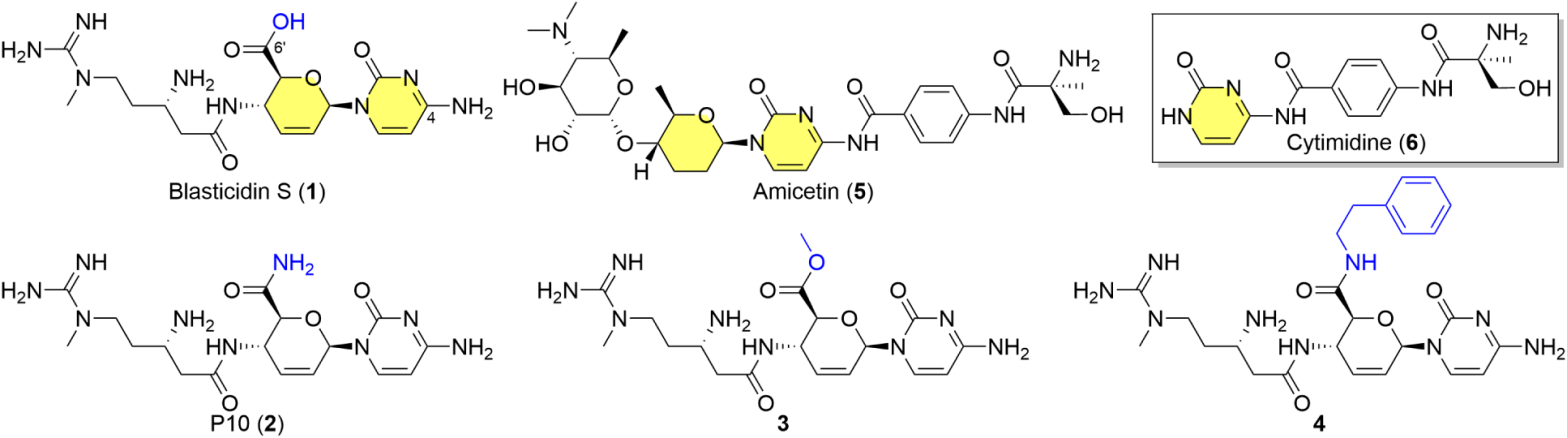
Structures of blasticidin S (**1**), P10 (**2**), blasticidin S methyl ester (**3**), blasticidin S phenethylamide (**4**), amicetin (**5**), and cytimidine (*inset*, **6**). The pyrimidine or nucleoside core (*yellow*) is shared while C6′ derivatization (*blue*) enhanced potency.

**Figure 2:**
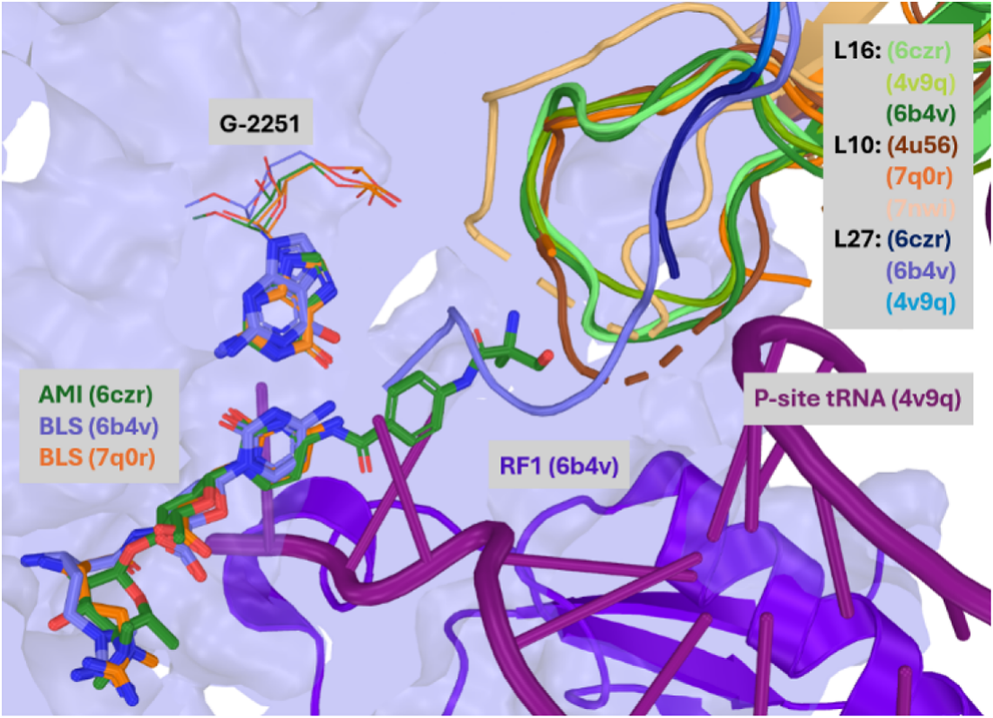
Overlay of amicetin (PDB ID: 6czr, *green sticks*) with blasticidin S bound to the bacterial ribosome (6b4v, *blue surface*). RF1 protein (PDB ID: 6b4v) and P-site tRNA (PDB ID: 4v9q) are shown in purple cartoons. The L27 proteins (*blue cartoons*) project toward the PABA extension of amicetin (clashing in the case of 6b4v) but have flexibility to shift as evidenced by the accommodation of the α-methylserine-PABA arm of amicetin in 6czr and the lack of sufficient electron density to model their termini in PDB IDs: 4v9q and 6czr. The clearly defined short loops of the bacterial protein L16 (*green cartoons*) also avoid the α-methylserine-PABA arm of amicetin, while the eukaryotic L10 proteins (*orange cartoons*) have 10–11 unresolved residues, suggesting a potential clash with the PABA arm.

Testing this structure-guided hypothesis required chemistry that could selectively and efficiently modify two distinct positions on the blasticidin S scaffold: the C6′ acid and the C4 amine. The scaffold’s multiple nucleophiles, acid/base lability, and solubility constraints,^2,14^ have historically made such transformations low-yielding or impractical. We therefore developed a semisynthetic route that exploits reactivity differences within the molecule to first selectively functionalizes the C6′ position followed by C4 amine coupling with amicetin-inspired derivatives. Key advances include counterion exchange to remove competing electrophiles, preventing side reactions, and selective protection to make the molecule tractable. This approach enabled access to densely functionalized blasticidin S derivatives without extensive protecting-group manipulation or onerous purifications.

Here we apply this route to generate four C6′ classes of blasticidin–amicetin chimeras, each diversified at C4 with PABA-derived arms. Antibacterial and cytotoxicity assays reveal that these chimeras retain potent antibacterial activity while exhibiting sharply reduced mammalian toxicity. Structural overlays of literature bacterial and eukaryotic ribosome complexes, supplemented by modeling, provide a plausible rationale for the observed selectivity gains, supporting the initial design hypothesis. This work demonstrates how structure-guided semisynthesis can transform a challenging natural product into selective translation inhibitors and outlines a generalizable strategy for selectively diversifying other chemically complex scaffolds.

## Results

To test the structure-guided design hypothesis, we prepared four series of blasticidin S–amicetin chimeras combining C6′ functional group diversity with C4 PABA extensions. These derivatives evaluated whether the semisynthetic route of selective C6′ modification followed by C4 amine coupling could deliver densely functionalized analogs with enhanced bacterial selectivity. Each series varied the C6′ group (methyl ester, primary amide, phenethyl amide, or carboxylate) while incorporating PABA-derived C4 arms up to the complete α-methylserine–PABA moiety, enabling direct comparison of both chemical and biological effects. As the largest derivative would be a combination of blasticidin S hybridized with the cytimidine^4^ fragment (**6**) of amicetin, the resulting series were named blastimidines M, A, P, and C, with the letter designator corresponding to the C6′ methyl ester (**3**), amide (**2**), phenethyl amide (**4**), and carboxylate (**1**), respectively. Derivatives already possessing more potent and selective C6′ functionality (blastimidines M and P), along with the active P10-derived primary amide series (blastimidines A), were prioritized over the blastimidines C, whose parent compound (blasticidin S with its intact acid) displayed the poorest antibacterial activity and selectivity. Nevertheless, synthesizing the full panel of blastimidine chimeras enabled a comprehensive structure–activity relationship (SAR) analysis of modifications at the C4 and C6′ positions. This complete data set demonstrates that a selective semisynthetic approach can be applied even to a scaffold as challenging as blasticidin S.

Toward the methyl ester series of blastimidine M chimeras (Scheme 1), esterification of blasticidin S (**1**) cleanly afforded **7**.^15^ Subsequent Boc protection furnished **8**, which could be prepared on scale using a novel adaptation of automated flash chromatography methods.^16^ Compound **8** was chosen as a common intermediate to enable selective use of the C4 amine as a nucleophile in peptide-coupling reactions. This selectivity arises from masking the nucleophilic β-amine as a Boc carbamate and removing the C6′ acid present in **1**, which would otherwise interfere with peptide coupling. Furthermore, the high p*K*_a_ (>12.5) of the guanidinium^14^ disfavors it as a nucleophile, making the C4 amine the most reactive site under peptide-coupling conditions.

**Scheme 1:**
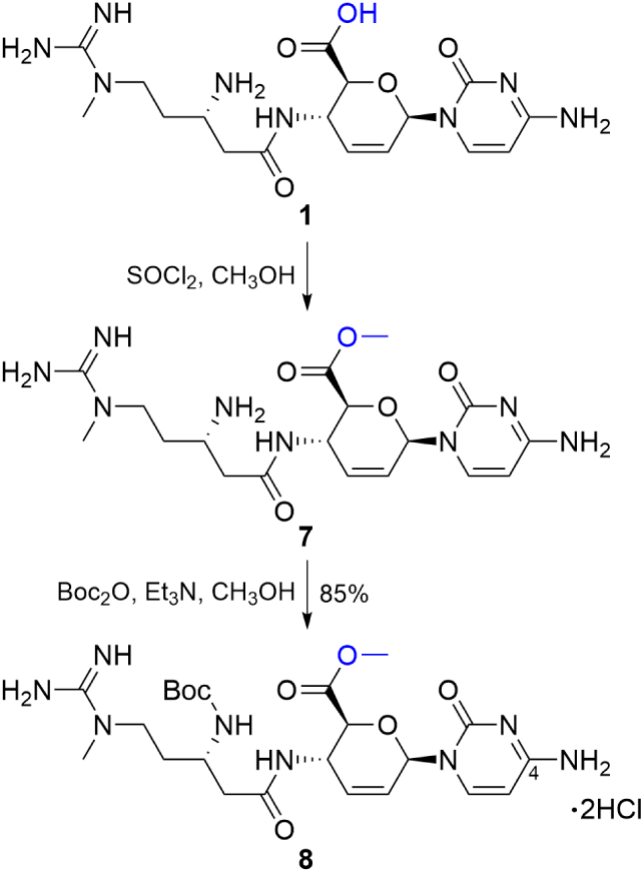
Synthesis of Protected Blasticidin S methyl ester 8

A peptide coupling screen between protected methyl ester **8** ^16^ and Boc-protected PABA (**9**) evaluated the performance of EDC, HATU, COMU, PyAOP, PyBOP, TNTU, and TSTU.^17^ Of these, only HATU, COMU, and PyAOP gave appreciable conversion with HATU being superior. Our previously established reversed-phase automated flash chromatography method reliably furnished pure **8** in a high yield, but as the monoformate salt, due to the use of formic acid as a solvent modifier during purification. To eliminate residual formic acid, which would participate in peptide-coupling reactions and reduce product yield, a modified protocol was developed in which impure **8** was treated with ten equivalents of aqueous 1 M HCl immediately before loading the sample for purification. By adding this excess mineral acid and using unmodified water/acetonitrile for elution, **8** was consistently isolated as the dihydrochloride salt with only a slight decrease in yield (85% versus 90% for the diformate salt).

Coupling was performed with HATU and DIPEA in DMF, and optimal yields were obtained when two equivalents of acid (**9**–**12**) and coupling reagent were used relative to amine **8**. Under these conditions, **8** and Boc-protected PABA (**9**) were coupled to produce **13** (Scheme 2) in an 84% yield. The same protocol was applied to Boc-protected *N*-methyl-PABA (**10**) and *N*-acetyl-PABA (**11**), furnishing **14** and **15**, respectively. While **13** and **14** were readily purified using reversed-phase flash chromatography, this method failed to fully separate **15** from unreacted **11**. Instead, TFA-mediated Boc cleavage was carried out directly on the mixture, which, after reversed-phase flash chromatography, successfully yielded pure **21**. Compounds **13** and **14** were also deprotected using TFA to afford **19** and **20**, respectively, in a similar overall yield.

**Scheme 2:**
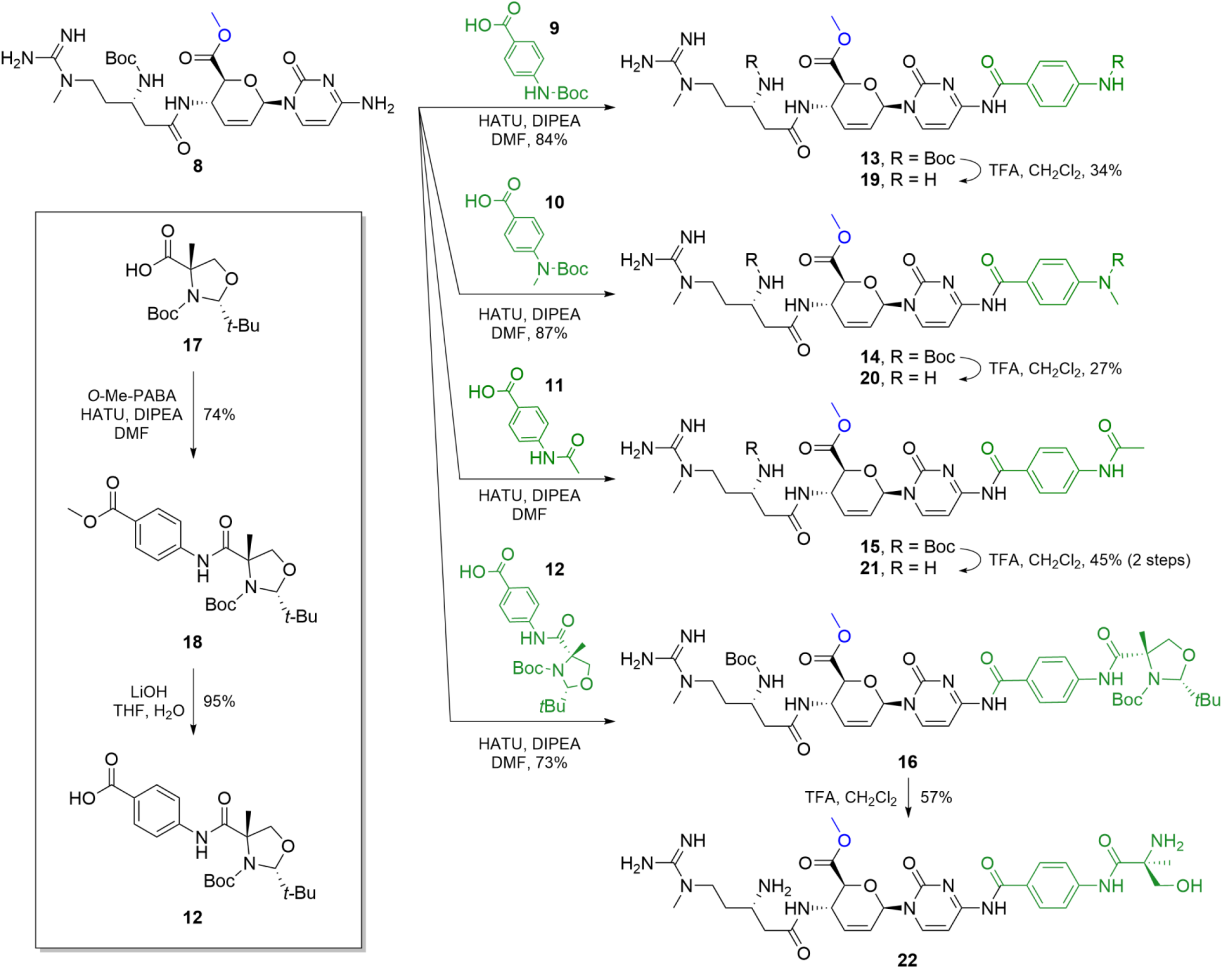
Synthesis of Protected Methyl Ester Intermediates **13–16** and Blastimidine M Hybrids **19**–**22** [Fn sch2-fn1]

a
*Inset*: Preparation of protected α-methylserine-PABA 12.

To access the full α-methylserine-PABA chimera (**22**), we first coupled a cyclic, protected form of α-methylserine (**17**, *inset*) with PABA methyl ester and hydrolyzed the resulting compound (**18**) to provide the free acid **12**.^18^ Prior work on protected amino acids of this type^18^ used a multistep sequence involving selective opening of the uncoupled oxazolidine (**18**) under acidic, nucleophilic conditions, protection of the resulting alcohol, hydrolysis to generate the PABA free acid, peptide coupling, and finally global deprotection of the Boc and alcohol protecting groups. As such, it was unclear if our direct approach of peptide coupling followed by deprotection would proceed without a good nucleophile (e.g., methanol or water) in solution for the deprotection. To our delight, HATU coupling of **12** with **8** achieved protected oxazolidine intermediate **16** in high yield, and subsequent TFA deprotection gave desired product **22** directly, completing the efficient synthesis of the blastimidine M series.

Thorough characterization of **22** after flash purification with formic acid as a modifier revealed one equivalent of formic acid by ^1^H NMR as well as the presence of trifluoroacetic acid (TFA) by ^13^C NMR. Quantitative ^19^F NMR, using α,α,α-trifluorotoluene as an external standard, confirmed two equivalents of TFA, indicating that **22** existed as a mixed salt form containing one equivalent of formic acid and two equivalents of TFA. To suppress this phenomenon, subsequent reversed-phase flash purification of blastimidines used TFA as the modifier, furnishing products exclusively as their TFA salt forms. Additional ^19^F NMR experiments revealed that the derivatives existed as either di- and tri-TFA salts, in accordance with the number of basic amines present on each blastimidine.

Toward the blastimidine A and P series, we adapted our previous nucleophilic displacement strategy where ammonia or phenethylamine reacted with the methyl ester of **8** to produce the corresponding amides (Scheme 3).^16^ This approach, combined with reversed-phase purification using hydrochloric acid in place of formic acid, furnished the protected divergent amide intermediates **23** and **24** as their dihydrochloride salts, suitable for peptide coupling.

**Scheme 3:**
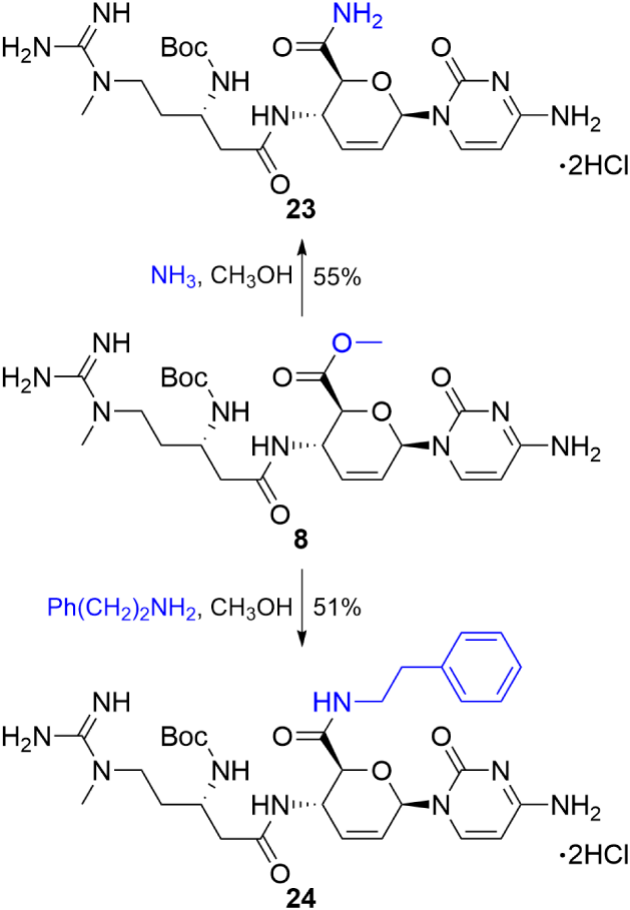
Synthesis of Diversifiable Intermediates for Blastimidines A (**23**) and P (**24**) from 8

Peptide coupling of **23** using the same conditions used for the blastimidine M series successfully yielded blastimidine A intermediates **25**–**28** (Scheme 4). Yields for the couplings were lower than those obtained with the methyl esters, potentially due to competition from the weakly nucleophilic primary amide. However, TFA-mediated deprotection of **25**–**28** produced **29**–**32** in higher yields than the corresponding reaction with the methyl ester, likely due to the increased acid stability of the amides.

**Scheme 4:**
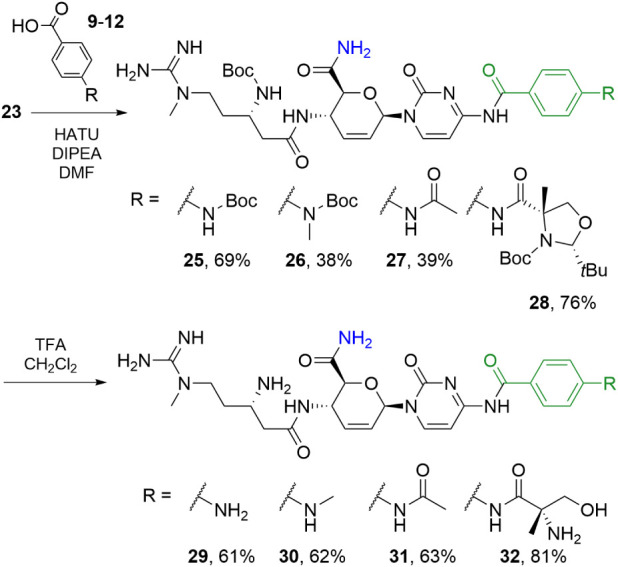
Synthesis of Protected Primary Amide Series **25–28** and Blastimidine A Hybrids **29–32**

The blastimidine P chimeras diverged from protected phenethyl amide 24 (Scheme 5) and proceeded similarly to the other series. Peptide couplings with HATU yielded protected intermediates **33**–**36**, although **33**–**35** coeluted with their starting acids. As with **15**, these mixtures were subjected to Boc deprotection. The two-step yields for **37**–**39** were comparable to those from earlier protocols and consistent with the overall yield of **40** via purified intermediate **36**.

**Scheme 5:**
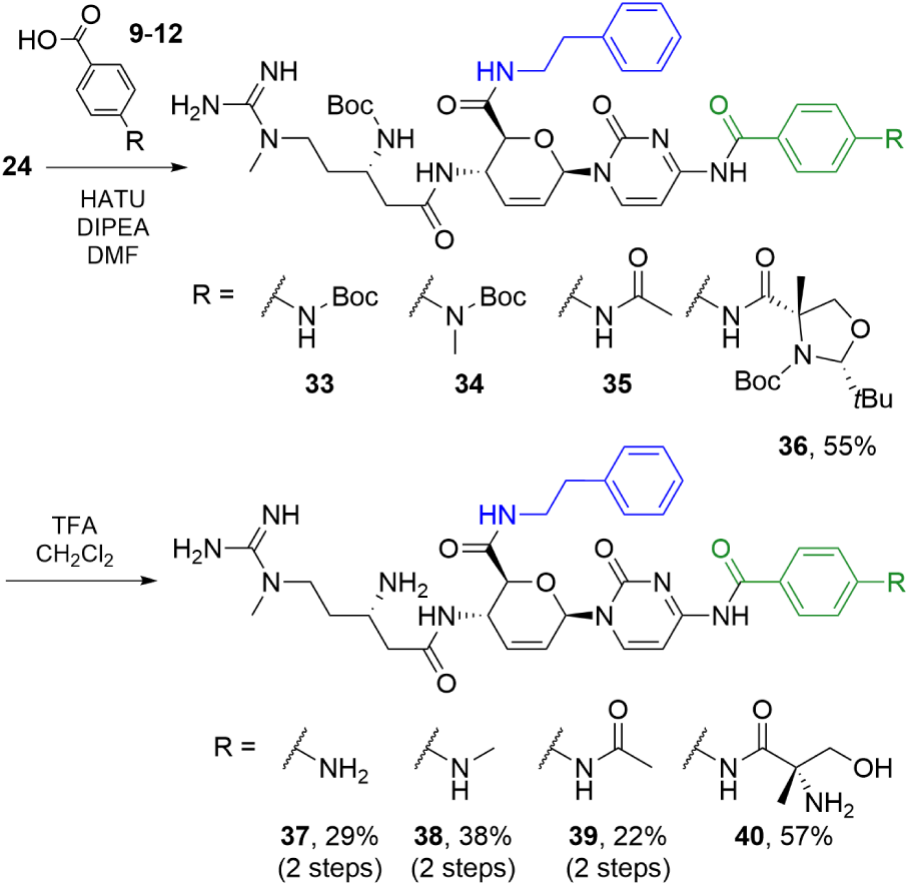
Synthesis of Protected Phenethyl Amide Series **33–36** and Blastimidine P Hybrids **37–40**

Hybridization of blasticidin S, which contains a free acid, into the blastimidine C series proved to be the most challenging. We initially attempted a protocol similar to that used for the other blastimidines, but Boc protection of **1** could only be optimized to a 50–60% yield, and subsequent peptide couplings were much lower yielding (∼20%). As an alternative, we attempted to hydrolyze the ester of the previously constructed intermediates from the blastimidine M series (**13**–**16**), aiming to access the free C6′ carboxylate. However, treatment of **13** with lithium hydroxide in water/THF did not result in the expected free acid; instead, the labile cytosine amide and other bonds underwent unwanted hydrolysis. A literature search revealed a somewhat unconventional method from the total synthesis of blasticidin S.^19^ In that work, triethylamine in methanol/water was used to hydrolyze the C6′ methyl ester. In our hands, however, these conditions resulted in complete solvolysis of the C4 PABA amide of **13** (Scheme 6) in addition to the hydrolysis of the ester (**41**). Because the methyl ester (**42**) was recovered and not the corresponding acid, we hypothesized that replacing methanol with acetonitrile might prevent solvolysis of the Boc-PABA moiety. Gratifyingly, treatment of **13** with triethylamine in acetonitrile/water afforded the target product.

**Scheme 6:**
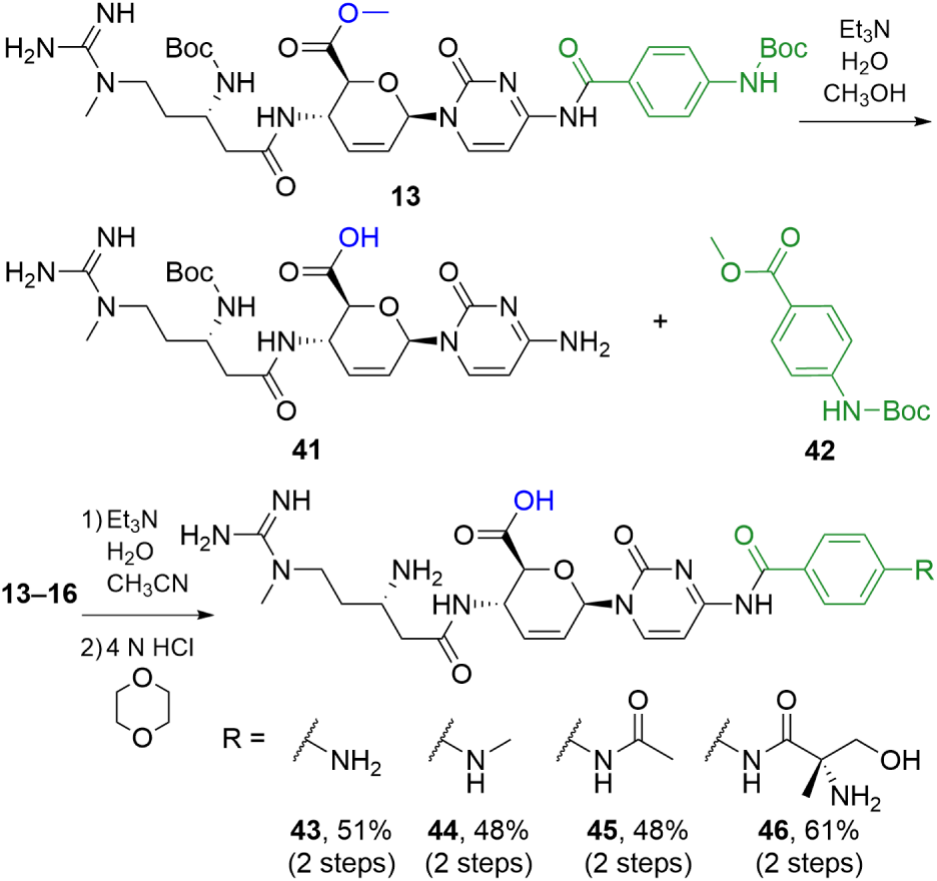
Synthetic Route to Carboxylate Blastimidine C Hybrids **43–46**

Based on our previous success using a two-step coupling/deprotection protocol, we intended to carry the acid intermediates constructed via this approach directly through deprotection without purification. Initial deprotection experiments in TFA resulted in peak duplication in the ^1^H NMR spectrum of the purified product **43**, likely due to partial disruption of an internal zwitterionic interaction—a complication not present with the blastimidine M, A, or P series. However, when HCl in 1,4-dioxane was used instead, no mixture was observed, and **43** was isolated cleanly as the dihydrochloride after reversed-phase flash purification with no acidic modifier. The remaining blastimidine C chimeras (**44**–**46**) were globally deprotected under the same conditions, thereby completing the synthesis of all four blastimidine series.

Our previous studies showed that modifying the C6′ acid of blasticidin S to esters^15^ or amides^16^ enhanced antimicrobial activity. Building on that insight, the four series of blastimidine chimeras were designed to examine how combining C6′ modification with C4 PABA hybridization influences biological outcomes. Antibacterial activity and cytotoxicity values, and especially their combination into a selectivity index (SI), provided critical insights, revealing clear trends in both potency and selectivity across the series.

**Table 1:**
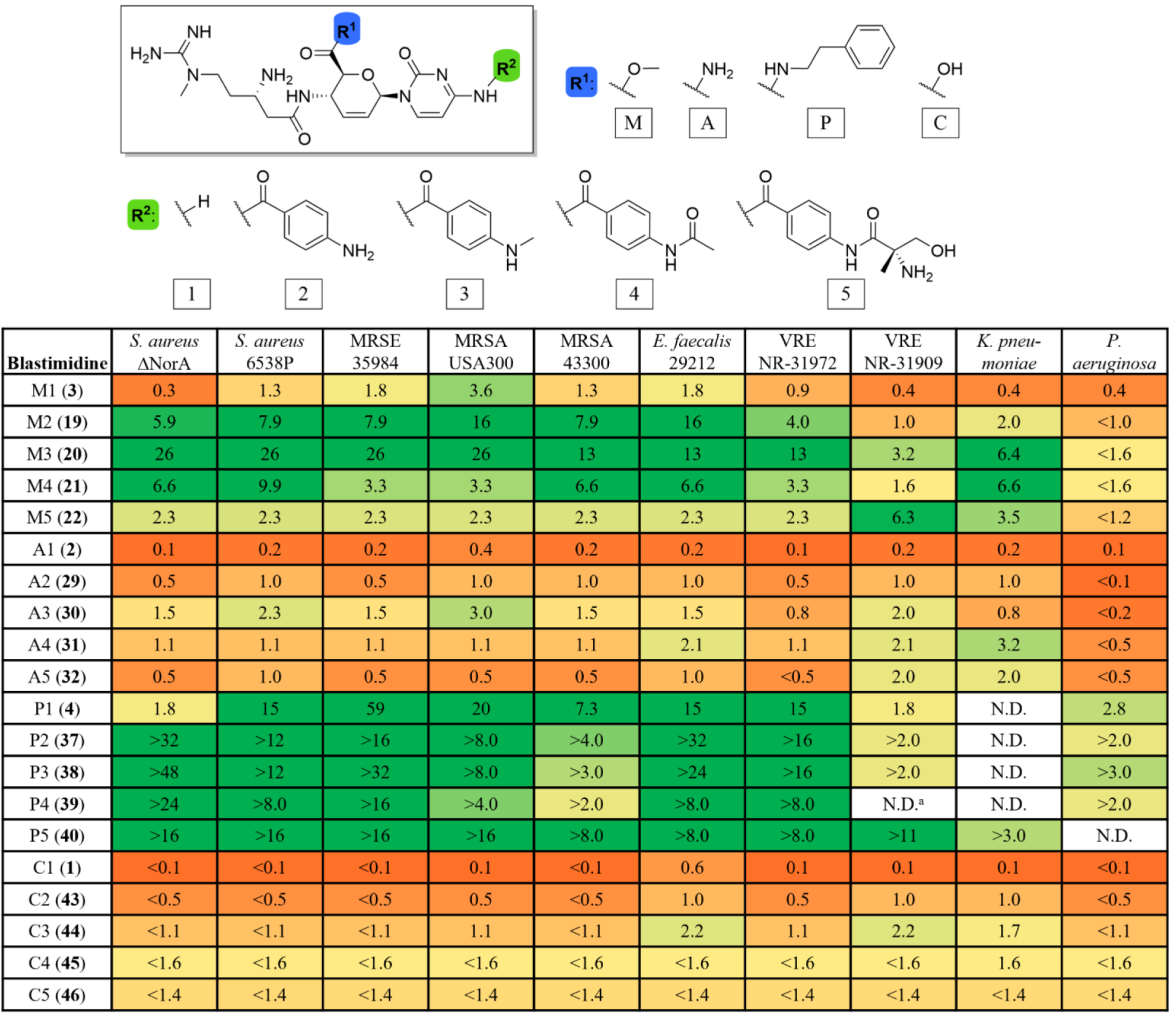
Selectivity Indices (CC_50_/MIC)^a^

a Not determined as both the MIC (>128 μg/mL) and CC_50_ (>256 μg/mL) values were out of test range.

Blastimidine C derivatives were largely inactive against bacteria (Table S1), consistent with the poor activity of the blasticidin S parent (C1). In contrast, activity across the blastimidine A, M, and P series generally improved over their respective parent compounds (A1, M1, P1), with peak activity observed in the midseries derivatives bearing PABA (A2, M2, P2) or methyl PABA (A3, M3, P3). The P series showed notable exceptions with P4 and P5 also exhibiting significant minimum inhibitory concentration (MIC) activity. Among the most active compounds, MIC values against Gram-positive pathogens ranged from 8–16 μg/mL with the M series performing best against *Staphylococcus aureus*, the A series against vancomycin-resistant enterococci (VRE), and the P series against *Enterococcus faecalis*. An increase in Gram-negative activity against *Klebsiella pneumoniae* was also observed for both the M and A series with MICs as low as 16 μg/mL.

Cytotoxicity testing revealed a substantial decrease in mammalian cell toxicity for blastimidine chimeras relative to their parent molecules (Table S2). The largest changes occurred in series 4 (acetyl-PABA), which showed >10-fold lower cytotoxicity for blastimidine C4. Series 3 (methyl-PABA) and series 5 (α-methylserine–PABA) also showed marked reductions (3–10-fold). For the blastimidine P series, CC_50_ values exceeded the highest tested concentration (256 μg/mL). Given that the P-series parent compound already exhibited low cytotoxicity (CC_50_ = 235 μg/mL) and identical modifications in the other series further reduced toxicity, the P derivatives are likely substantially less toxic.

The selectivity index (SI), which integrates antibacterial potency (MIC, Table S1) and mammalian cytotoxicity (CC_50_, Table S2) into a single value (SI = CC_50_/MIC), is a key metric for therapeutic potential. SI values >50 generally indicate a favorable therapeutic window. While blastimidines M, A, and P showed broadly comparable antimicrobial activity (Table S1), the amide series (A1–A5) was significantly more cytotoxic than the others (Table S2), resulting in poor selectivity. As shown in Table 1, SI values for the A series remained in the single digits for Gram-positive pathogens—on par with the inactive C series—and are thus unsuitable for further development. Within the M series, M3 showed the highest selectivity (SI > 25 against most *S. aureus* strains) with M2 and M4 also outperforming M1. The M series also showed selectivity for *K. pneumoniae*, a high-priority ESKAPE pathogen.^20^

The standout compounds were the blastimidine P series. Derivatives P2 and P3 showed the greatest measurable enhancement in selectivity against susceptible *S. aureus* (up to an SI of >48 for P3 against the NorA efflux pump-deficient strain) and *E. faecalis*. The largest derivative, P5, showed consistent selectivity across all Gram-positive organisms, including MRSA and VRE. Notably, because their cytotoxicity is >256 μg/mL, the true SI values for the P series relative to the P1 parent are likely several-fold higher than the calculated values, if a similar trend is seen as in the other series. The high molecular weight of the PABA derivatives and the TFA counterions also inflates the reported MICs when expressed in μg/mL; potencies would appear more favorable if normalized to molarity or measured with lower-mass counterions.

To assess bacteriostatic or bactericidal effects, a time-kill assay was performed against MRSA USA-300 using blastimidine P3 (**38**) and P5 (**40**). As shown in Figure S1, P3 demonstrated bacteriostatic activity at 5× MIC and 10× MIC, with 2.5 and 2.7 log reductions, respectively, after 24 h. In contrast, P5 showed bactericidal activity and complete clearance at both 5× MIC and 10× MIC within the same time frame. The control linezolid displayed bacteriostatic activity, whereas vancomycin achieved bactericidal activity by 8 h. Multistep resistance selection via 15 days of serial passaging^21–23^ did not produce a resistant phenotype (Figure S2).

## Discussion

This study set out to test the hypothesis that installing an amicetin-inspired PABA arm onto blasticidin S could preserve productive binding in bacterial termination complexes while disfavoring binding during eukaryotic elongation. Across four C6′ substitution series, the antibacterial and cytotoxicity data support this premise, with the most pronounced selectivity gains observed in the phenethyl amide (blastimidine P) series and in midseries PABA or methyl-PABA derivatives at C4. Structural overlays and modeling suggest that these modifications engage a bacterial-specific pocket near the peptidyl transferase center (PTC) that is conformationally disfavored in eukaryotic elongation, providing a clear mechanistic rationale for the observed selectivity trends.

The observed enhancements likely arise from species-specific differences in how ribosome-bound small molecules affect elongation versus termination. In bacteria, blasticidin S primarily inhibits termination,^3,6^ whereas in eukaryotes, elongation inhibition predominates.^8^ Overlays of literature ribosome structures show that blasticidin S and amicetin share a common binding site (Figure 2). In termination-state bacterial structures, the nearby loop of L16 ^11^ is small (3–4 residues) and the terminus of L27 ^12^ appears conformationally flexible—capable of accommodating the PABA arm and consistent with retention of antibacterial activity of the blastimidines. In eukaryotic structures these nearby positions are occupied by the larger, flexible loop of protein L10.^13^

Molecular docking of P5 into the bacterial ribosome (PDB ID: 6czr) revealed poses consistent with the ribosome cocrystallized with blasticidin or amicetin (Figure 3), suggesting that blastimidines would maintain a similar mode of binding. Combined with AlphaFold3 *de novo* modeling^24^ to visualize the unresolved residues of the eukaryotic L10 protein (PDB ID: 7q0r), these studies also suggest that the PABA arm would clash sterically with this protein in eukaryotes, reducing productive binding and thereby diminishing the effect of elongation inhibition. This structural rationale aligns with the pronounced drop in mammalian cytotoxicity across the blastimidine series, most notably in the P-series derivatives where CC_50_ values were >256 μg/mL. The aromatic feature of the phenethylamide arm of the P series could engage in favorable contacts with the underlying ribosome nucleotides, enhancing its antimicrobial activity.^16^ Alternatively, its substitution for the negatively charged carboxylate and hydrophobic character may facilitate better uptake into bacteria, achieving the same end.

**Figure 3:**
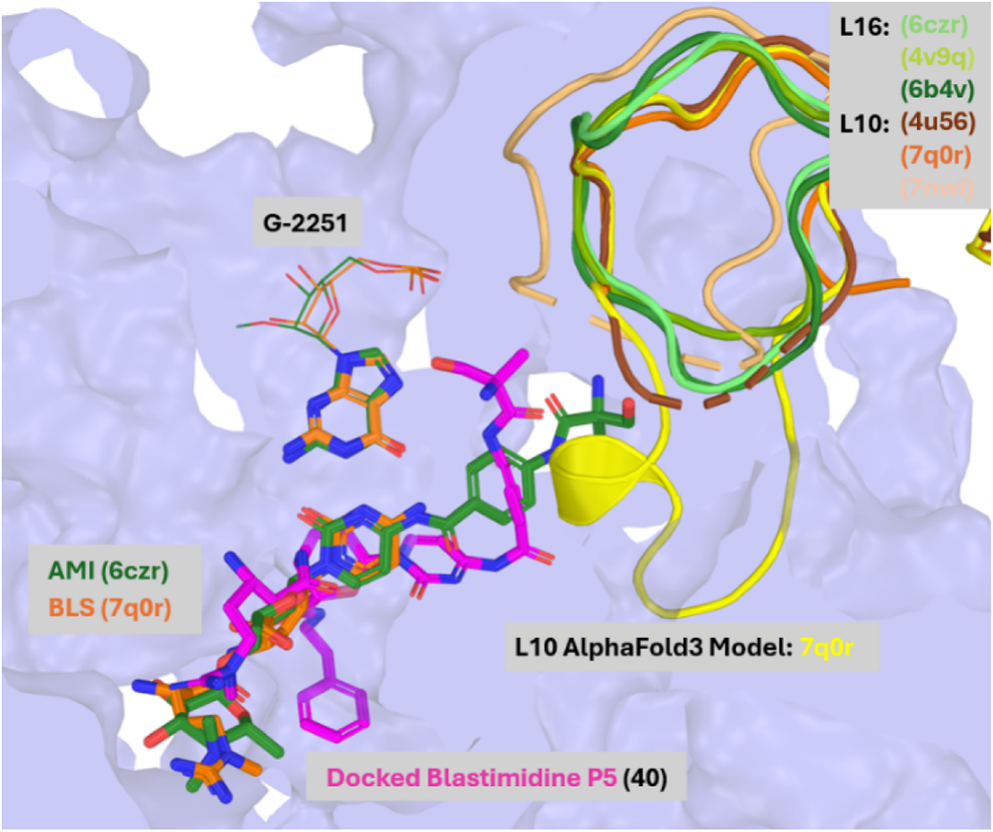
Docking of chimera blastimidine P5 (**40**, *magenta*) to the bacterial ribosome (PDB ID: 6czr). The new PABA arm is accommodated within the defined short loops of the bacterial protein L16, suggesting continued interference with termination. AlphaFold3 ^24^ modeling of the L10 protein from 7q0r to visualize the missing residues of L10 (*yellow cartoon*) shows a steric clash with the α-methylserine–PABA arm of amicetin (*green*) and P5, suggesting reduced interference with elongation in eukaryotes.

The semisynthetic platform developed here was critical for testing this hypothesis. By enabling orthogonal diversification at both the C6′ and C4 positions on a chemically complex scaffold, it allowed systematic structure–activity relationship mapping across both steric and electronic dimensions. Counterion exchange to remove reactive formate prior to peptide coupling proved essential for accessing high-yield C4 amidations, a tactic that should be broadly applicable to other polycationic natural products prone to counterion-mediated side reactions. The ability to carry out these transformations without extensive protecting-group manipulation or high-intensity purifications makes this approach adaptable to other scaffolds with multiple highly polar functional groups.

Taken together, these results demonstrate that a historically cytotoxic translation inhibitor can be converted into bacterial-selective derivatives with clear therapeutic potential using a tailored, structure-guided semisynthetic approach. More broadly, the work illustrates how combining precise synthetic access with structure-based design can reengineer the binding profiles of complex natural products, enabling efficient access to both mechanistic probes and lead compounds without the need for specialized biosynthetic precursors or lengthy total synthetic campaigns.

## Conclusions

This work combines synthetic methodology and chemical biology to convert a historically cytotoxic natural product into selective antibacterial leads. A practical semisynthetic route enabling sequential C6′ and C4 modification of blasticidin S delivered four classes of densely functionalized blasticidin–amicetin chimeras. Across the series, antibacterial potency was retained while mammalian cytotoxicity dropped sharply, with the most favorable derivatives approaching selectivity indices near the benchmark value of 50.

Mechanistic analysis using modeling on established ribosome structures supports the design premise: the PABA arm extension of the blastimidines likely engages a bacterial-specific pocket available during termination but binding is sterically disfavored in eukaryotic elongation, providing a structural rationale for the observed selectivity gains. More broadly, this work illustrates how hypothesis-driven semisynthesis can transform chemically complex scaffolds into selective, mechanism-guided probes and lead compounds, offering a generalizable strategy applicable to other natural products where inherent reactivity should be harnessed rather than avoided.

## Supplementary Material



## Notes

Complete experimental procedures, spectral data, biological assays, and computational details (PDF)

## ¶

Xiretsa Pharmaceuticals, Santa Barbara, CA 93106, USA

## Abbreviations

PABA: *para*-aminobenzoic acid
PTC: peptidyl transferase center
TFA: trifluoroacetic acid
NMR: nuclear magnetic resonance
MRSA: methicillin-resistant *Staphylococcus aureus*
VRE: vancomycin-resistant enterococci
MIC: minimum inhibitory concentration
CC_50_: 50% cytotoxic concentration
SI: selectivity index

## References

[1] Gould S. J. (1997). Blasticidin
S and related peptidyl nucleoside antibiotics. Drugs Pharm. Sci..

[2] Takeuchi S., Hirayama K., Ueda K., Sakai H., Yonehara H. (1958). Blasticidin
S, a new antibiotic. J. Antibiot., Ser. A.

[3] Svidritskiy E., Ling C., Ermolenko D. N., Korostelev A. A. (2013). Blasticidin
S inhibits translation by trapping deformed tRNA on the ribosome. Proc. Natl. Acad. Sci. U.S.A..

[4] Haskell T. H. (1958). Amicetin,
Bamicetin and Plicacetin. Chemical studies. J. Am. Chem. Soc..

[5] Nelli M. R., Heitmeier K. N., Looper R. E. (2021). Dissecting the nucleoside antibiotics
as universal translation inhibitors. Acc. Chem.
Res..

[6] Svidritskiy E., Korostelev A. A. (2018). Mechanism of inhibition of translation
termination
by blasticidin S. J. Mol. Biol..

[7] Garreau
de Loubresse N., Prokhorova I., Holtkamp W., Rodnina M. V., Yusupova G., Yusupov M. (2014). Structural basis for the inhibition
of the eukaryotic ribosome. Nature.

[8] Powers K. T., Stevenson-Jones F., Yadav S. K. N., Amthor B., Bufton J. C., Borucu U., Shen D., Becker J. P., Lavysh D., Hentze M. W., Kulozik A. E., Neu-Yilik G., Schaffitzel C. (2021). Blasticidin
S inhibits mammalian translation and enhances
production of protein encoded by nonsense mRNA. Nucleic Acids Res..

[9] Zgadzay Y., Kolosova O., Stetsenko A., Wu C., Bruchlen D., Usachev K., Validov S., Jenner L., Rogachev A., Yusupova G., Sachs M. S., Guskov A., Yusupov M. (2022). E-site drug
specificity of the human pathogen Candida albicans ribosome. Sci. Adv..

[10] Serrano C. M., Kanna-Reddy H. R., Eiler D., Koch M., Tresco B. I. C., Barrows L. R., VanderLinden R. T., Testa C. A., Sebahar P. R., Looper R. E. (2020). Unifying the aminohexopyranose- and peptidyl-nucleoside
antibiotics: Implications for antibiotic design. Angew. Chem., Int. Ed..

[11] Maimets T., Remme J., Villems R. (1984). Ribosomal
protein L16 binds to the
3′-end of transfer RNA. FEBS Lett..

[12] Maguire B. A., Beniaminov A. D., Ramu H., Mankin A. S., Zimmermann R. A. (2005). A protein
component at the heart of an RNA machine: The importance of protein
L27 for the function of the bacterial ribosome. Mol. Cell.

[13] Pollutri D., Penzo M. (2020). Ribosomal protein L10: From function
to dysfunction. Cells.

[14] Otake N., Takeuchi S., Endo T., Yonehara H. (1965). Structure of blasticidin
S. Tetrahedron Lett..

[15] Gannett C., Banks P., Chuong C., Weger-Lucarelli J., Mevers E., Lowell A. N. (2023). Semisynthetic blasticidin
S ester
derivatives show enhanced antibiotic activity. RSC Med. Chem..

[16] Gannett C., Tiller K., Briganti A. J., Brown A. M., Weger-Lucarelli J., Lowell A. N. (2024). Forgotten natural products: Semisynthetic development
of blasticidin S as an antibiotic lead. ACS
Med. Chem. Lett..

[17] El-Faham A., Albericio F. (2011). Peptide coupling
reagents, more than a letter soup. Chem. Rev..

[18] Fu J., Xu P., Yu B. (2021). Total synthesis
of nucleoside antibiotics amicetin,
plicacetin, and cytosaminomycin A—D. Chin. J. Chem..

[19] Ichikawa Y., Hirata K., Ohbayashi M., Isobe M. (2004). Total synthesis of
(+)-blasticidin S. Chem. —Eur. J..

[20] Wyres K. L., Holt K. E. (2018). Klebsiella pneumoniae
as a key trafficker of drug resistance
genes from environmental to clinically important bacteria. Curr. Opin. Microbiol..

[21] Mohammad H., Abutaleb N. S., Seleem M. N. (2020). Auranofin
Rapidly Eradicates Methicillin-resistant
Staphylococcus aureus (MRSA) in an Infected Pressure Ulcer Mouse Model. Sci. Rep..

[22] Mohammad H., Kyei-Baffour K., Abutaleb N. S., Dai M., Seleem M. N. (2019). An aryl
isonitrile compound with an improved physicochemical profile that
is effective in two mouse models of multidrug-resistant Staphylococcus
aureus infection. J. Global Antimicrob. Resist..

[23] Hagras M., Hegazy Y. A., Elkabbany A. H., Mohammad H., Ghiaty A., Abdelghany T. M., Seleem M. N., Mayhoub A. S. (2018). Biphenylthiazole
antibiotics with an oxadiazole linker: An approach to improve physicochemical
properties and oral bioavailability. Eur. J.
Med. Chem..

[24] Abramson J., Adler J., Dunger J., Evans R., Green T., Pritzel A., Ronneberger O., Willmore L., Ballard A. J., Bambrick J., Bodenstein S. W., Evans D. A., Hung C.-C., O’Neill M., Reiman D., Tunyasuvunakool K., Wu Z., Žemgulytė A., Arvaniti E., Beattie C., Bertolli O., Bridgland A., Cherepanov A., Congreve M., Cowen-Rivers A. I., Cowie A., Figurnov M., Fuchs F. B., Gladman H., Jain R., Khan Y. A., Low C. M. R., Perlin K., Potapenko A., Savy P., Singh S., Stecula A., Thillaisundaram A., Tong C., Yakneen S., Zhong E. D., Zielinski M., Žídek A., Bapst V., Kohli P., Jaderberg M., Hassabis D., Jumper J. M. (2024). Accurate structure prediction of
biomolecular interactions with AlphaFold 3. Nature.

