# Supplementary material for: Structure-Guided Semisynthesis of Blasticidin S–Amicetin Chimeras as Selective Ribosome Inhibitors
Source: J Am Chem Soc. 2026 Jan 12;148(3):2963–71. doi: 10.1021/jacs.5c13979 (PMC12856912; doi:10.1021/jacs.5c13979)
Supplement: Supplementary file 1 [file ja5c13979_si_001.pdf]

Structure-Guided Semisynthesis of Blasticidin S–Amicetin Chimeras as Selective Ribosome Inhibitors

Cole Gannett,[a,b,c] Kateland Tiller,[b,c,d] Somaia Abdelmegeed,[d] Micah Hoernig,[c,e] Ahmed A. Abouelkhair,[b,f] Mohamed N. Seleem,[b,c,d,f] James Weger-Lucarelli,[b,c,d,f], Anne M. Brown,[b,c,e,g] Andrew N. Lowell[a,b,c,h,]*

[a] Department of Chemistry, Virginia Polytechnic Institute and State University (Virginia Tech), Blacksburg, VA 24061, USA

[b] Center for Emerging, Zoonotic, and Arthropod-borne Pathogens, Virginia Tech, Blacksburg, VA 24061, USA

[c] Virginia Tech Center for Drug Discovery, Virginia Tech, Blacksburg, VA 24061, USA

[d] Department of Biomedical Sciences and Pathobiology, Virginia Tech, VA-MD Regional College of Veterinary Medicine, Blacksburg, VA 24061, United States

[e] Department of Biochemistry, Virginia Tech, Blacksburg, VA 24061, USA

[f] Center for One Health Research, Virginia Tech, Blacksburg, VA 24061, USA

[g] Research and Informatics, University Libraries, Virginia Tech, Blacksburg, VA 24061, USA

[h] Faculty of Health Sciences, Virginia Polytechnic Institute and State University (Virginia Tech), Blacksburg, VA 24061, United States

Table of Contents

Title Page S1

Table of Contents S2

General Experimental S3–S4

Synthetic Experimental S4–S29

Biological Assays S30–S34

Table S1: MIC Values S32

Table S2: CC_{50} Values S33

Figure S1: Timekill Results S34

Figure S2: Multi-step resistance selection S35

Structural Comparisons and Computational Modeling S35

NMR Spectra S36–S59

References S60

General Experimental

Blasticidin S hydrochloride was purchased from Diagnocine. Unless otherwise specified, all reagents, solvents, and media components were purchased commercially and used as received from Sigma Aldrich, Fisher Scientific, or Oakwood Chemical. Deionized water was obtained from the house deionized water system. All synthetic reactions were stirred with a magnetic stir bar under a nitrogen atmosphere unless otherwise stated.

Specific rotations were obtained on a Jasco P-2000 polarimeter. ^{1}H NMR spectra were recorded on a Bruker Avance II 500 MHz spectrometer, Agilent U4-DD2 400 MHz spectrometer, or Bruker Avance III 600 MHz spectrometer. Chemical shifts are reported in parts per million (ppm) using the solvent resonance as an internal standard (CD_3OD 3.31 ppm, D_2O 4.79 ppm). Data are reported as follows: chemical shift, multiplicity (s=singlet, d=doublet, t=triplet, q=quartet, quint=quintet, sext=sextet, m=multiplet), coupling constants (Hz), and number of protons. Proton decoupled ^{13}C NMR were recorded on a Bruker Avance II 500 MHz (^{13}C 125 MHz) spectrometer or an Agilent U4-DD2 400 MHz (^{13}C 100 MHz) spectrometer. Chemical shifts are reported in ppm using the solvent resonance as an internal standard (CD_3OD 49.0 ppm). ^{19}F NMR spectra were recorded on a JEOL 400 MHz spectrometer. NMR signals attributed to the counteranions (formate, trifluoroacetate) for salts of **19–22**, **29–32**, **37–40**, and **43–46** are not tabulated but included in yield calculations. High resolution mass spectra were obtained on an Agilent Technologies 6220 TOF LC/MS, a Waters Synapt Q-TOF G2, or Thermo Exploris 120 HESI Orbitrap MS in the Department of Chemistry or the VT-Mass Spectrometry Incubator at the Virginia Polytechnic Institute and State University. Automated flash chromatography was performed using a Biotage Selekt system using unmodified water or water modified with one of the following: 0.1% (v/v) formic acid, 0.1% (v/v) TFA, or 0.05 % (v/v) TFA as solvent A and unmodified acetonitrile

(CH_3CN) or CH_3CN modified with one of the following: 0.1% (v/v) formic acid, 0.1% (v/v) TFA, or 0.05 % (v/v) TFA as solvent B. Commercial C18 cartridges were purchased from Biotage.

Synthetic Experimental

Methyl (2*S*,3*S*,6*R*)-6-(4-amino-2-oxopyrimidin-1(2*H*)-yl)-3-((*S*)-3-((*tert*-butoxycarbonyl)amino)-5-(1-methylguanidino)pentanamido)-3,6-dihydro-2*H*-pyran-2-carboxylate (8) Dihydrochloride.

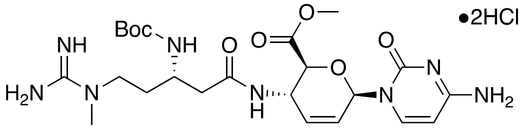

Blasticidin S **1** (1.02 g, 2.21 mmol) was suspended in anhydrous CH_3OH (131 mL) and cooled in an ice bath. Thionyl chloride (4.81 mL, 66.3 mmol) was added slowly while the cold suspension was stirring, resulting in a solution. The solution was warmed to rt and after stirring for 20 h, was partially concentrated under a stream of dry nitrogen and then fully concentrated under vacuum. The resulting solids were dissolved in methanol and concentrated. Dissolution in methanol and reconcentration was repeated before drying under vacuum to yield **7** as the trihydrochloride salt which was used directly in the next reaction.

Compound **7** (1.21 g, 2.21 mmol) was dissolved in anhydrous methanol (24.3 mL) and cooled (ice bath). Triethylamine (0.72 mL, 5.2 mmol) was added to the stirring mixture, followed by Boc anhydride (0.67 mL, 2.9 mmol) and the mixture was warmed to rt. After 18 h, the mixture was concentrated under vacuum to yield a white solid. This residue was dissolved in deionized water (3 mL) and acidified with 1 M HCl (aq.) to pH 1. This solution was purified using automated flash chromatography (C18, 30 g, 0-60% B unmodified) to yield the dihydrochloride salt of **8** (1.20 g, 85%) as a white amorphous solid. Spectral data were in accord with those previously reported.[1]

Methyl (2*S*,3*S*,6*R*)-3-((*S*)-3-((*tert*-butoxycarbonyl)amino)-5-(1-methylguanidino)pentanamido)-6-(4-(4-((*tert*-butoxycarbonyl)amino)benzamido)-2-oxopyrimidin-1(2*H*)-yl)-3,6-dihydro-2*H*-pyran-2-carboxylate (13).

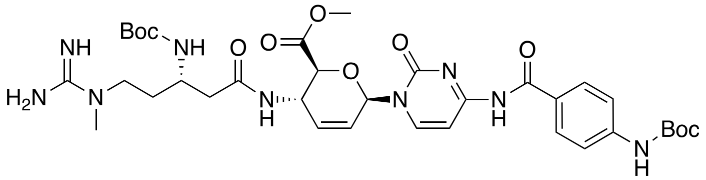

N-Boc-PABA **9** (61.6 mg, 0.257 mmol), HATU (98.9 mg, 0.260 mmol), and DIPEA (66.7 µL, 0.383 mmol) were dissolved in anhydrous DMF (0.75 mL) and stirred at rt for 10 min. Compound **8** (77.8 mg, 0.128 mmol) was added as a solution in anhydrous DMF (0.85 mL) and the mixture was heated (oil bath, 70 °C). After 4 h, the reaction was quenched by the addition of CH_3OH (2 mL) and immediately concentrated to a yellow residue. The residue was dissolved in CH_3OH and concentrated onto Celite for solid-loaded automated flash chromatography (C18, 30g, 0-60% B, solvents modified w/ 0.1% formic acid), which yielded **13** (81.1 mg, 84%) as a fluffy white solid: $[\alpha]_D^{24}$ = +52° (*c* 0.85, CH_3OH); ^{1}H NMR (500 MHz, CD_3OD) δ 7.98 (d, *J* = 7.6 Hz, 1H), 7.92 (d, *J* = 8.9 Hz, 2H), 7.61–7.56 (m, 3H), 6.65 (q, *J* = 2.1 Hz, 1H), 6.18 (dt, *J* = 10.3, 2.3 Hz, 1H), 5.97 (dt, *J* = 10.2, 2.1 Hz, 1H), 4.87–4.81 (m, 1H), 4.39 (d, *J* = 8.5 Hz, 1H), 3.91–3.84 (m, 1H), 3.72 (s, 3H), 3.50–3.32 (m, 2H), 3.04 (s, 3H), 2.47 (dd, *J* = 14.4, 5.8 Hz, 1H), 2.39 (dd, *J* = 14.4, 7.4 Hz, 1H), 1.95–1.86 (m, 1H), 1.75 (dtd, *J* = 14.1, 9.3, 5.0 Hz, 1H), 1.53 (s, 9H), 1.45 (s, 9H); ^{13}C NMR (125 MHz, CD_3OD) δ 172.9, 170.4, 168.4, 165.4, 158.3, 157.9, 157.8, 154.6, 147.0, 145.7, 134.2, 130.5, 127.6, 127.0, 118.7, 99.3, 81.5, 81.2, 80.5, 77.3, 53.4, 49.0, 47.1, 46.0, 42.3, 36.6, 32.5, 28.7, 28.6; HRMS (ESI) calcd for $C_{35}H_{50}N_9O_{10}$ $[M+H]^+$ 756.3675, found 756.3690.

Methyl (2*S*,3*S*,6*R*)-6-(4-(4-((*tert*-butoxycarbonyl)(methyl)amino)benzamido)-2-oxopyrimidin-1(2*H*)-yl)-3-((*S*)-3-((*tert*-butoxycarbonyl)amino)-5-(1-methylguanidino)pentanamido)-3,6-dihydro-2*H*-pyran-2-carboxylate (14)

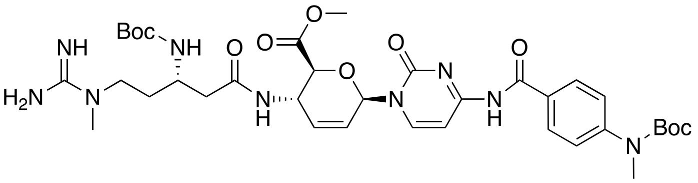

N-Boc-*N*-methyl-PABA **10** (68.7 mg, 0.273 mmol), HATU (101.4 mg, 0.267 mmol), and DIPEA (70.0 μL, 0.402 mmol) were dissolved in anhydrous DMF (0.75 mL) and stirred at rt for 10 min. Compound **8** (80.9 mg, 0.133 mmol) was added as a solution in anhydrous DMF (0.85 mL) and the mixture was heated (oil bath, 70 °C). After 4 h, the reaction was cooled to rt and quenched by the addition of anhydrous CH_3OH (1.0 mL) and immediately concentrated to a yellow-orange residue. The residue was dissolved in CH_3OH and concentrated onto Celite for solid-loaded automated flash chromatography (C18, 30g, 0-60% B, solvents modified w/ 0.1% formic acid) which yielded **14** (86.9 mg, 87%) as a fluffy white solid: $[\alpha]_D^{22}$ = +45° (*c* 2.1, CH_3OH); ^{1}H NMR (500 MHz, CD_3OD) δ 8.01 (d, *J* = 7.5 Hz, 1H), 7.98 (d, *J* = 8.8 Hz, 2H), 7.61 (d, *J* = 7.5 Hz, 1H), 7.47 (d, *J* = 8.7 Hz, 2H), 6.65 (q, *J* = 2.1 Hz, 1H), 6.18 (dt, *J* = 10.2, 2.3 Hz, 1H), 5.98 (dt, *J* = 10.1, 2.1 Hz, 1H), 4.87–4.84 (m, 1H), 4.39 (d, *J* = 8.5 Hz, 1H), 3.91–3.83 (m, 1H), 3.73 (s, 3H), 3.51–3.33 (m, 3H), 3.31 (s, 3H), 3.05 (s, 3H), 2.47 (dd, *J* = 14.4, 5.9 Hz, 1H), 2.39 (dd, *J* = 14.4, 7.4 Hz, 1H), 1.95–1.86 (m, 1H), 1.75 (dtd, *J* = 14.1, 9.3, 5.0 Hz, 1H), 1.48 (s, 9H), 1.46 (s, 9H); ^{13}C NMR (125 MHz, CD_3OD) δ 172.9, 170.3, 168.5, 165.4, 158.3, 157.8, 155.9, 149.3, 147.2, 134.2, 131.1, 129.9, 127.0, 126.2, 99.3, 82.5, 81.2, 80.5, 77.3, 53.4, 49.0 (2C), 47.1, 46.0, 42.3, 37.4, 36.7, 32.5, 28.7, 28.5; HRMS (ESI) calcd for $C_{36}H_{52}N_9O_{10}$ $[M+H]^+$ 770.3832, found 770.3824.

***tert*-Butyl (2*R*,4*S*)-4-((4-((1-((2*R*,5*S*,6*S*)-5-((*S*)-3-((*tert*-butoxycarbonyl)amino)-5-(1-methylguanidino)pentanamido)-6-(methoxycarbonyl)-5,6-dihydro-2*H*-pyran-2-yl)-2-oxo-1,2-dihydropyrimidin-4-yl)carbamoyl)phenyl)carbamoyl)-2-(*tert*-butyl)-4-methyloxazolidine-3-carboxylate (16)**

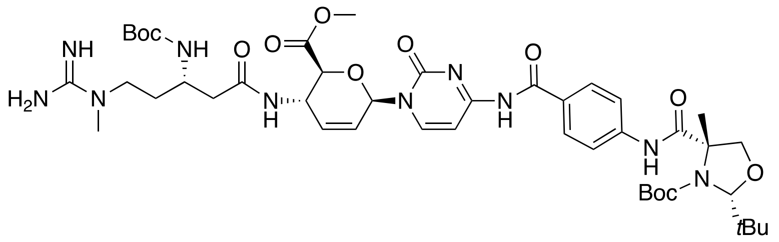

PABA-oxazolidine **12**[2] (121 mg, 0.298 mmol), HATU (112 mg, 0.296 mmol), and DIPEA (69.3 μL, 0.398 mmol) were dissolved in anhydrous DMF (0.75 mL) and stirred for 5 min at rt. Compound **8** (80.8 mg, 0.133 mmol), was added as a solution in anhydrous DMF (0.85 mL) and the reaction mixture was heated (oil bath, 75 °C) for 1.5 h. The solution was concentrated to yield a yellow residue which was purified using reverse-phase automated flash chromatography (C18, 30g, 0-100% B, solvents modified w/ 0.1% formic acid). The fractions containing **16** were concentrated to remove volatiles and then the remaining aqueous solution was diluted with saturated aqueous Na_2CO_3 (10 mL) and extracted with ethyl acetate (3 x 15 mL). The combined organic layers were dried over Na_2SO_4 and concentrated to yield **16** (89.3 mg, 73%) as a white amorphous solid: $[\alpha]_D^{23}$ = +82° (*c* 4.5, CH_3CN); ^1H NMR (400 MHz, CD_3OD) δ 8.04–7.98 (m, 3H), 7.75–7.68 (m, 2H), 7.61 (d, *J* = 7.5 Hz, 1H), 6.65 (q, *J* = 2.0 Hz, 1H), 6.18 (dt, *J* = 10.3, 2.3 Hz, 1H), 5.98 (ddd, *J* = 10.3, 2.5, 1.6 Hz, 1H), 5.19 (s, 1H), 4.90–4.85 (m, 1H), 4.70 (d, *J* = 8.9 Hz, 1H), 4.39 (d, *J* = 8.5 Hz, 1H), 3.92–3.83 (m, 1H), 3.80 (d, *J* = 8.9 Hz, 1H), 3.73 (s, 3H), 3.52–3.32 (m, 2H), 3.05 (s, 3H), 2.47 (dd, *J* = 14.4, 5.7 Hz, 1H), 2.39 (dd, *J* = 14.4, 7.4 Hz, 1H), 1.97–1.85(m, 1H), 1.81–1.71 (m, 1H), 1.71 (s, 3H), 1.55 (s, 9H), 1.46 (s, 9H), 0.94 (s, 9H); ^{13}C NMR

(125 MHz, CD_3OD) δ 173.2, 172.9, 170.4, 168.2, 165.3, 158.2, 157.8, 157.08, 157.06, 147.2, 143.7, 134.3, 130.7, 129.8, 127.0, 120.3, 99.3, 99.0, 84.1, 81.2, 80.5, 77.2, 76.8, 69.4, 53.4, 49.0, 47.1, 46.0, 42.3, 39.1, 36.6, 32.5, 28.7, 28.4, 26.6, 22.0; HRMS (ESI) calcd for $C_{44}H_{65}N_{10}O_{12}$ $[M+H]^+$ 925.4778, found 925.4788.

Methyl (2*S*,3*S*,6*R*)-3-((*S*)-3-amino-5-(1-methylguanidino)pentanamido)-6-(4-(4-aminobenzamido)-2-oxopyrimidin-1(2*H*)-yl)-3,6-dihydro-2*H*-pyran-2-carboxylate (19) tri-TFA salt

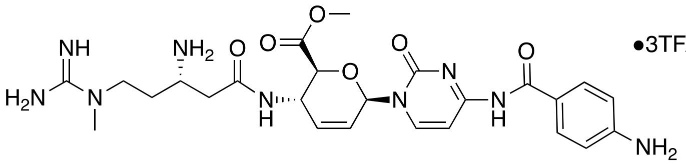

Compound **13** (81.1 mg, 0.101 mmol) was dissolved in 50% (v/v) TFA/CH_2Cl_2 (5 mL) while cooling (ice bath). After 2 h, the solution was concentrated under vacuum and the resulting residue was diluted with benzene (4 mL) and concentrated. The benzene treatment was repeated two more times and the resulting residue dried under vacuum overnight. The residue was purified using automated flash chromatography (C18, 30 g, 0-50% B, solvents modified w/ 0.1% TFA) to yield the tri-TFA salt of **19** (31.1 mg, 35%) as a glassy yellow solid: $[\alpha]_D^{23}$ = +54° (*c* 1.3, CH_3OH); ^1H NMR (500 MHz, CD_3OD) δ 7.98 (d, *J* = 7.6 Hz, 1H), 7.82–7.77 (m, 2H), 7.54 (d, *J* = 7.5 Hz, 1H), 6.76–6.71 (m, 2H), 6.65 (q, *J* = 2.2 Hz, 1H), 6.18 (dt, *J* = 10.2, 2.3 Hz, 1H), 6.00 (ddd, *J* = 10.3, 2.5, 1.7 Hz, 1H), 4.96–4.92 (m, 1H), 4.42 (d, *J* = 8.4 Hz, 1H), 3.73 (s, 3H), 3.60 (p, *J* = 6.4 Hz, 1H), 3.49 (td, *J* = 7.4, 3.1 Hz, 2H), 3.09 (s, 3H), 2.73 (dd, *J* = 16.3, 5.2 Hz, 1H), 2.61 (dd, *J* = 16.4, 6.6 Hz, 1H), 2.02 (dt, *J* = 9.5, 6.9 Hz, 2H); ^{13}C NMR (125 MHz, CD_3OD) δ 171.7, 170.3, 168.7, 164.8, 158.3, 156.0, 153.8, 147.7, 134.1, 131.7, 127.0, 121.7, 115.4, 98.9, 81.2, 77.2, 53.4, 48.1,

47.8, 45.9, 37.4, 36.7, 30.8; HRMS (ESI) calcd for $C_{25}H_{34}N_9O_6$ $[M+H]^+$ 556.2627, found 556.2624.

Methyl (2*S*,3*S*,6*R*)-3-((*S*)-3-amino-5-(1-methylguanidino)pentanamido)-6-(4-(4-(methylamino)benzamido)-2-oxopyrimidin-1(2*H*)-yl)-3,6-dihydro-2*H*-pyran-2-carboxylate (20) tri-TFA salt

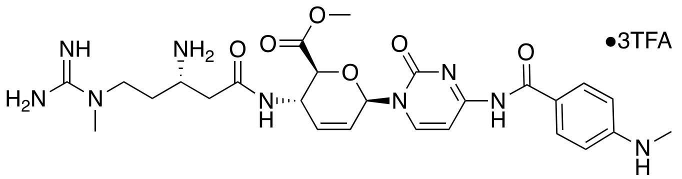

Compound **14** (86.9 mg, 0.113 mmol) was dissolved in 50% (v/v) TFA/CH_2Cl_2 (5 mL) while cooling (ice bath). After 2 h, the solution was concentrated under vacuum and the resulting residue was diluted with benzene (4 mL) and concentrated under vacuum. The benzene treatment was repeated two more times and the residue dried under vacuum overnight. The residue was purified using automated flash chromatography (C18, 30 g, 0-50% B, solvents modified w/ 0.1% TFA) to yield the tri-TFA salt of **20** (28.2 mg, 27%) as a glassy yellow/orange solid: $[\alpha]_D^{23}$ = +50° (*c* 1.2, CH_3OH); ^1H NMR (500 MHz, CD_3OD) δ 8.00 (d, *J* = 7.5 Hz, 1H), 7.86–7.82 (m, 2H), 7.55–7.45 (m, 1H), 6.67–6.63 (m, 3H), 6.19 (dt, *J* = 10.3, 2.3 Hz, 1H), 6.00 (ddd, *J* = 10.3, 2.5, 1.7 Hz, 1H), 4.95–4.92 (m, 1H), 4.43 (d, *J* = 8.4 Hz, 1H), 3.73 (s, 3H), 3.63–3.56 (m, 1H), 3.49 (td, *J* = 7.3, 2.2 Hz, 2H), 3.09 (s, 3H), 2.85 (s, 3H), 2.73 (dd, *J* = 16.3, 5.2 Hz, 1H), 2.61 (dd, *J* = 16.3, 6.6 Hz, 1H), 2.02 (dt, *J* = 9.6, 6.9 Hz, 2H); ^{13}C NMR (125 MHz, CD_3OD) δ 171.7, 170.3, 168.8, 164.1, 158.3, 156.0, 154.4, 148.4, 134.2, 131.8, 126.8, 119.3, 112.3, 98.5, 81.2, 77.1, 53.4, 48.0, 47.8, 45.9, 37.4, 36.7, 30.8, 29.9; HRMS (ESI) calcd for $C_{26}H_{36}N_9O_6$ $[M+H]^+$ 570.2783, found 570.2801.

Methyl (2*S*,3*S*,6*R*)-6-(4-(4-acetamidobenzamido)-2-oxopyrimidin-1(2*H*)-yl)-3-((*S*)-3-amino-5-(1-methylguanidino)pentanamido)-3,6-dihydro-2*H*-pyran-2-carboxylate (21) di-TFA salt

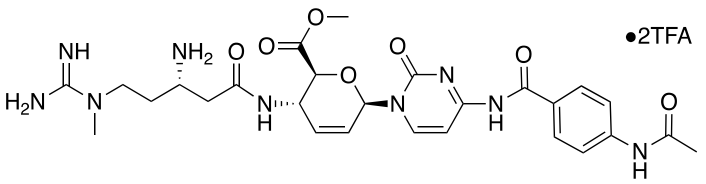

N-Acetyl-PABA **11** (54.5 mg, 0.304 mmol), HATU (112 mg, 0.295 mmol), and DIPEA (0.80 mL, 0.44 mmol) were dissolved in anhydrous DMF (0.85 mL) and stirred at rt for 10 min. Boc BLS methyl ester dihydrochloride **8** (89.1 mg, 0.146 mmol) was added as a solution in anhydrous DMF (0.85 mL) and the mixture was heated (oil bath, 70 °C). After 4 h, the reaction was cooled to rt and quenched with anhydrous CH_3OH (1.0 mL) and immediately concentrated by rotary evaporation and subsequently under vacuum to yield a clear and colorless residue, which was dissolved in CH_3OH and concentrated onto Celite. Solid-loaded automated flash chromatography (C18, 30 g, 0-60% B, solvents modified w/ 0.1% formic acid) yielded **15** as a mixture with **11** as an off-white solid. This mixture was used in the next reaction without additional purification.

The mixture of **15** and **11** was dissolved in 50% (v/v) TFA/CH_2Cl_2 (2.0 mL) while cooling (ice bath). After 2 h, the solution was concentrated and the resulting residue was diluted with benzene (4 mL) and concentrated under vacuum. The benzene treatment was repeated two more times and the resulting residue dried under vacuum overnight. The residue was purified using automated flash chromatography (C18, 30 g, 0-50% B, solvents modified w/ 0.1% TFA) to yield the di-TFA salt of **21** (54.5 mg, 45%, 2 steps) as a glassy off-white solid: $[\alpha]_D^{23}$ = +52° (*c* 2.4, CH_3OH); ^1H NMR (500 MHz, CD_3OD) δ 8.00 (d, *J* = 7.5 Hz, 1H), 7.99–7.95 (m, 2H), 7.78–7.74 (m, 2H), 7.60 (d, *J* = 7.5 Hz, 1H), 6.66 (q, *J* = 2.0 Hz, 1H), 6.18 (dt, *J* = 10.3, 2.3 Hz, 1H), 6.01 (ddd, *J* = 10.4, 2.5, 1.6 Hz, 1H), 4.95–4.93 (m, 1H), 4.43 (d, *J* = 8.4 Hz, 1H), 3.74 (s, 3H), 3.60

(td, J = 6.8, 5.4 Hz, 1H), 3.50 (td, J = 7.5, 4.0 Hz, 2H), 3.09 (s, 3H), 2.73 (dd, J = 16.3, 5.2 Hz, 1H), 2.61 (dd, J = 16.2, 6.5 Hz, 1H), 2.17 (s, 3H), 2.02 (dt, J = 9.4, 7.0 Hz, 2H); ^{13}C NMR (125 MHz, CD_3OD) δ 172.1, 171.7, 170.4, 168.4, 165.1, 158.3, 157.2, 147.5, 144.8, 134.0, 130.5, 129.0, 127.1, 120.2, 99.2, 81.2, 77.1, 53.4, 48.1, 47.8, 45.9, 37.4, 36.7, 30.8, 24.1; HRMS (ESI) calcd for $C_{27}H_{36}N_9O_7$ $[M+H]^+$ 598.2732, found 598.2723.

Methyl (2*S*,3*S*,6*R*)-6-(4-(4-((*S*)-2-amino-3-hydroxy-2-methylpropanamido)benzamido)-2-oxopyrimidin-1(2*H*)-yl)-3-((*S*)-3-amino-5-(1-methylguanidino)pentanamido)-3,6-dihydro-2*H*-pyran-2-carboxylate (22) mono-HCOOH, di-TFA mixed salt

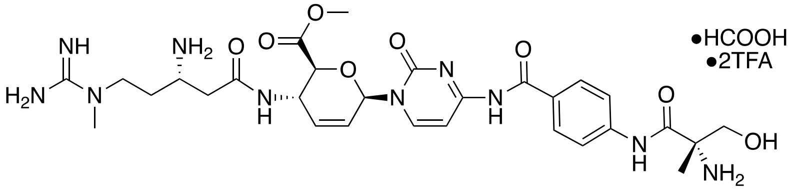

To a cold (ice bath), stirring solution of **16** in CH_2Cl_2 (2.0 mL) was added TFA (2.0 mL). After 1.5 h, the mixture was concentrated to yield a clear brown oil. The oil was diluted with benzene (2.0 mL) and concentrated. The benzene treatment was repeated two more times and the resulting residue was dried under vacuum to yield a foamy, off-white amorphous solid. Purification using automated flash chromatography (C18, 30g, 0-30% B, solvents modified w/ 0.1% formic acid) yielded a mixed salt, 1 HCOOH and 2 TFA, of **22** (47.3 mg, 57%) as an off-white, glassy solid: $[\alpha]_D^{22}$ = +55° (*c* 2.0, CH_3OH); ^{1}H NMR (400 MHz, CD_3OD) δ 8.00 (d, J = 8.0 Hz, 3H), 7.83 (d, J = 8.8 Hz, 2H), 7.60 (d, J = 7.5 Hz, 1H), 6.66 (q, J = 1.9 Hz, 1H), 6.18 (dt, J = 10.3, 2.2 Hz, 1H), 5.99 (dt, J = 10.2, 2.1 Hz, 1H), 4.95–4.90 (m, 1H), 4.43 (d, J = 8.6 Hz, 1H), 4.09 (d, J = 11.8 Hz, 1H), 3.82 (d, J = 11.8 Hz, 1H), 3.73 (s, 3H), 3.62–3.44 (m, 3H), 3.08 (s, 3H), 2.72 (dd, J = 16.2, 5.1 Hz, 1H), 2.62 (dd, J = 16.1, 6.6 Hz, 1H), 2.01 (q, J = 7.5 Hz, 2H), 1.64 (s, 3H); ^{13}C NMR (126 MHz, CD_3OD) δ 171.9, 170.6, 170.4, 168.3, 165.3, 158.4, 157.8, 147.3, 143.8, 134.0, 130.4, 130.1,

127.2, 121.2, 99.3, 81.2, 77.2, 65.7, 63.4, 53.4, 48.1, 47.7, 46.0, 37.9, 36.6, 30.9, 19.1; HRMS (ESI) calcd for $C_{29}H_{41}N_{10}O_8$ $[M+H]^+$ 657.3103, found 657.3089.

***tert*-Butyl ((*S*)-1-(((2*S*,3*S*,6*R*)-6-(4-amino-2-oxopyrimidin-1(2*H*)-yl)-2-carbamoyl-3,6-dihydro-2*H*-pyran-3-yl)amino)-5-(1-methylguanidino)-1-oxopentan-3-yl)carbamate (23) Dihydrochloride**

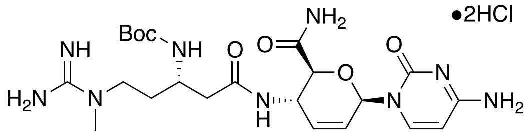

Compound **8** (203 mg, 0.348 mmol) was dissolved in 7 M ammonia in methanol (4.1 mL) and stirred at room temperature in a sealed vial. After 48 h, the solution was concentrated under a stream of nitrogen to yield a white solid, which was dissolved in deionized water (3 mL) and acidified with 1 M HCl (aq.) to pH 1. This solution was purified using automated flash chromatography (C18, 30 g, 0-60% B, unmodified) to yield the dihydrochloride salt of **23** (114 mg, 55%) as glassy white solid. Spectral data were in accord with those previously reported.[1]

***tert*-Butyl ((*S*)-1-(((2*S*,3*S*,6*R*)-6-(4-(4-((*tert*-butoxycarbonyl)amino)benzamido)-2-oxopyrimidin-1(2*H*)-yl)-2-carbamoyl-3,6-dihydro-2*H*-pyran-3-yl)amino)-5-(1-methylguanidino)-1-oxopentan-3-yl)carbamate (25)**

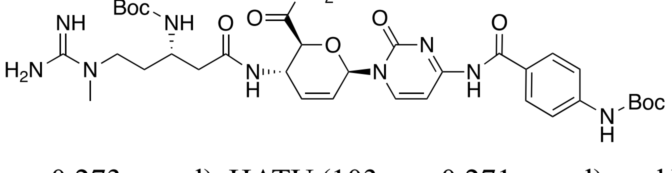

N-Boc-PABA **9** (64.7 mg, 0.273 mmol), HATU (103 mg, 0.271 mmol), and DIPEA (70.4 µL, 0.404 mmol) were dissolved in anhydrous DMF (0.8 mL) and stirred at rt for 10 min. Compound **23** (80.1 mg, 0.135 mmol) dissolved in anhydrous DMF (0.8 mL) was added and the solution was

heated (oil bath, 70 °C). After 3.5 h, the mixture was cooled and quenched by the addition of CH_3OH (3 mL) and immediately concentrated by rotary evaporation and subsequently under vacuum to yield a yellow/orange residue. The residue was dissolved in CH_3OH and concentrated onto Celite. Solid-loaded automated flash chromatography (C18, 30 g, 0-60% B, solvents modified w/ 0.1% formic acid) followed by concentrated to remove organics and then the lyophilized yielded **25** (69.0 mg, 69%) as a fluffy white solid: $[\alpha]_D^{23}$ = +75° (*c* 3.3, CH_3OH); ^1H NMR (400 MHz, CD_3OD) δ 8.06 (d, *J* = 7.5 Hz, 1H), 7.98–7.89 (m, 2H), 7.65 (d, *J* = 7.6 Hz, 1H), 7.62–7.56 (m, 2H), 6.69 (dt, *J* = 3.4, 1.8 Hz, 1H), 6.12 (dt, *J* = 10.2, 2.0 Hz, 1H), 5.92 (ddd, *J* = 10.3, 2.6, 1.5 Hz, 1H), 4.85–4.81 (m, 1H), 4.27 (d, *J* = 9.3 Hz, 1H), 3.84 (td, *J* = 8.7, 4.2 Hz, 1H), 3.48 (ddd, *J* = 15.6, 9.8, 6.4 Hz, 1H), 3.37–3.26 (m, 1H), 3.04 (s, 3H), 2.51 (dd, *J* = 13.7, 4.9 Hz, 1H), 2.31 (dd, *J* = 13.6, 8.6 Hz, 1H), 2.02–1.90 (m, 1H), 1.79–1.66 (m, 1H), 1.54 (s, 9H), 1.46 (s, 9H); ^{13}C NMR (125 MHz, CD_3OD) δ 172.9, 172.7, 168.3, 165.4, 158.1, 158.0, 157.9, 154.6, 147.1, 145.7, 135.2, 130.5, 127.5, 126.6, 118.6, 99.5, 81.6, 81.5, 80.6, 77.8, 49.0, 47.6, 46.5, 43.1, 36.6, 31.9, 28.7, 28.6; HRMS (ESI) calcd for $C_{34}H_{48}N_{10}O_9$ $[M+H]^+$ 741.3678, found 741.3668.

***tert*-Butyl (4-((1-((2*R*,5*S*,6*S*)-5-((*S*)-3-((*tert*-butoxycarbonyl)amino)-5-(1-methylguanidino)pentanamido)-6-carbamoyl-5,6-dihydro-2*H*-pyran-2-yl)-2-oxo-1,2-dihydropyrimidin-4-yl)carbamoyl)phenyl)(methyl)carbamate (26)**

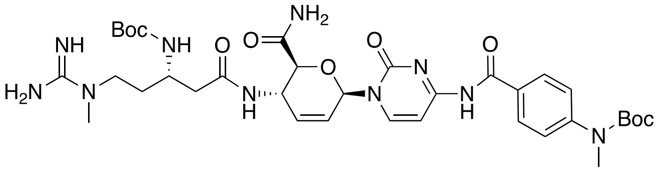

N-Boc-*N*-methyl-PABA **10** (70.3 mg, 0.280 mmol), HATU (105 mg, 0.277 mmol), and DIPEA (71.9 μL, 0.413 mmol) were dissolved in anhydrous DMF (0.84 mL) and stirred at rt for 10 min. Compound **23** (81.8 mg, 0.138 mmol) dissolved in anhydrous DMF (0.8 mL) was added and the

resulting mixture was heated in an oil bath (70 °C). After 4 h, the reaction was concentrated under vacuum to yield a yellow/orange residue, which was dissolved in CH_3OH and concentrated onto Celite. Solid-loaded automated flash chromatography (C18, 30 g, 0-70% B, solvents modified w/ 0.1% formic acid) followed by concentrated to remove organics and then lyophilization yielded **26** (38.6 mg, 38%) as a fluffy white solid: $[\alpha]_D^{23}$ = +70° (*c* 1.9, CH_3OH); ^1H NMR (500 MHz, CD_3OD) δ 8.08 (d, *J* = 7.6 Hz, 1H), 8.01–7.96 (m, 2H), 7.63 (d, *J* = 7.5 Hz, 1H), 7.50–7.45 (m, 2H), 6.69 (dt, *J* = 3.3, 1.8 Hz, 1H), 6.12 (dt, *J* = 10.2, 2.0 Hz, 1H), 5.93 (ddd, *J* = 10.3, 2.6, 1.5 Hz, 1H), 4.84–4.82 (m, 1H), 4.28 (d, *J* = 9.3 Hz, 1H), 3.90–3.79 (m, 1H), 3.48 (ddd, *J* = 15.9, 9.8, 6.5 Hz, 1H), 3.39–3.25 (m, 1H), 3.31 (s, 3H), 3.04 (s, 3H), 2.51 (dd, *J* = 13.7, 4.9 Hz, 1H), 2.31 (dd, *J* = 13.7, 8.6 Hz, 1H), 2.02–1.91 (m, 1H), 1.78–1.67 (m, 1H), 1.48 (s, 9H), 1.46 (s, 9H); ^{13}C NMR (125 MHz, CD_3OD) δ 172.9, 172.7, 168.4, 165.4, 158.2, 157.92, 157.87, 155.8, 149.3, 147.3, 135.2, 130.9, 129.9, 126.6, 126.2, 99.5, 82.5, 81.7, 80.6, 77.8, 49.0, 47.6, 46.5, 43.1, 37.4, 36.6, 31.9, 28.7, 28.6; HRMS (ESI) calcd for $C_{35}H_{51}N_{10}O_9$ $[M+H]^+$ 755.3835, found 755.3827.

***tert*-Butyl ((*S*)-1-(((2*S*,3*S*,6*R*)-6-(4-(4-acetamidobenzamido)-2-oxopyrimidin-1(2*H*)-yl)-2-carbamoyl-3,6-dihydro-2*H*-pyran-3-yl)amino)-5-(1-methylguanidino)-1-oxopentan-3-yl)carbamate (27)**

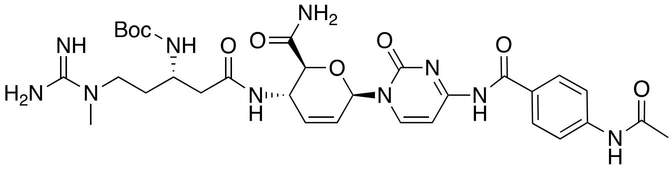

N-Acetyl-PABA **11** (53.7 mg, 0.300 mmol), HATU (109 mg, 0.286 mmol), and DIPEA (71.9 μL, 0.412 mmol) were dissolved in anhydrous DMF (0.84 mL) and stirred at rt for 10 min. Boc P10 dihydrochloride **23** (81.7 mg, 0.137 mmol) dissolved in anhydrous DMF (0.8 mL) was added and

the resulting mixture was heated in an oil bath (70 °C). After 4 h, the reaction was concentrated to yield a faintly yellow residue, which was dissolved in CH_3OH/CH_3CN and concentrated onto Celite. Solid-loaded automated flash chromatography (C18, 30 g, 0-70% B, solvents modified w/ 0.1% formic acid) yielded **27** (36.2 mg, 39%) as a glassy, white solid: $[\alpha]_D^{27}$ = +52° (*c* 1.9, 50% CH_3OH/H_2O); ^1H NMR (400 MHz, CD_3OD) δ 8.06 (d, *J* = 7.5 Hz, 1H), 8.00–7.93 (m, 2H), 7.78–7.72 (m, 2H), 7.63 (d, *J* = 7.6 Hz, 1H), 6.69 (dt, *J* = 3.4, 1.8 Hz, 1H), 6.12 (dt, *J* = 10.1, 2.1 Hz, 1H), 5.93 (ddd, *J* = 10.2, 2.6, 1.5 Hz, 1H), 4.84–4.79 (m, 1H), 4.27 (d, *J* = 9.3 Hz, 1H), 3.89–3.79 (m, 1H), 3.48 (ddd, *J* = 15.6, 9.8, 6.6 Hz, 1H), 3.37–3.27 (m, 1H), 3.04 (s, 3H), 2.51 (dd, *J* = 13.7, 4.8 Hz, 1H), 2.31 (dd, *J* = 13.0, 9.4 Hz, 1H), 2.17 (s, 3H), 2.02–1.90 (m, 1H), 1.79–1.66 (m, 1H), 1.46 (s, 9H); ^{13}C NMR (125 MHz, CD_3OD) δ 172.9, 172.7, 172.0, 168.3, 165.4, 158.2, 157.93, 157.90, 147.2, 144.6, 135.2, 130.4, 129.1, 126.6, 120.1, 99.5, 81.7, 80.6, 77.8, 49.0, 47.6, 46.5, 43.1, 36.6, 31.9, 28.7, 24.1; HRMS (ESI) calcd for $C_{31}H_{43}N_{10}O_8$ $[M+H]^+$ 683.3260, found 683.3281.

***tert*-Butyl (2*R*,4*S*)-4-((4-((1-((2*R*,5*S*,6*S*)-5-((*S*)-3-((*tert*-butoxycarbonyl)amino)-5-(1-methylguanidino)pentanamido)-6-carbamoyl-5,6-dihydro-2*H*-pyran-2-yl)-2-oxo-1,2-dihydropyrimidin-4-yl)carbamoyl)phenyl)carbamoyl)-2-(*tert*-butyl)-4-methyloxazolidine-3-carboxylate (28)**

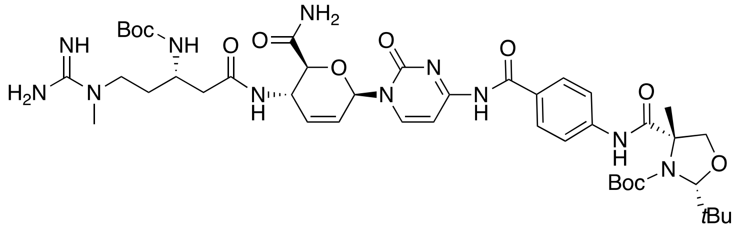

PABA-oxazolidine **12** (106 mg, 0.261 mmol), HATU (100.7 mg, 0.2648 mmol), and DIPEA (68.9 μL, 0.396 mmol) were dissolved in anhydrous DMF (0.7 mL) and stirred for 5 min at rt. Compound

23 (78.4 mg, 0.132 mmol) dissolved in anhydrous DMF (0.9 mL) was added and the mixture was heated (oil bath, 70 °C). After 2 h, the mixture was cooled and concentrated to yield a yellow residue. After adsorption onto Celite, purification using reverse-phase automated flash chromatography (C18, 30g, 0-100% B, solvents modified w/ 0.1% formic acid) yielded **28** (91.0 mg, 76%) as a white, amorphous solid: $[\alpha]_D^{24}$ = +145° (*c* 4.7, CH_3OH); ^1H NMR (500 MHz, CD_3OD) δ 8.07 (d, *J* = 7.6 Hz, 1H), 8.04–7.98 (m, 2H), 7.76–7.69 (m, 2H), 7.64 (s, 1H), 6.69 (q, *J* = 2.3 Hz, 1H), 6.12 (dt, *J* = 10.2, 2.1 Hz, 1H), 5.93 (ddd, *J* = 10.2, 2.6, 1.6 Hz, 1H), 5.19 (s, 1H), 4.84–4.79 (m, 1H), 4.70 (d, *J* = 8.9 Hz, 1H), 4.27 (d, *J* = 9.4 Hz, 1H), 3.89–3.77 (m, 1H), 3.81 (d, *J* = 8.9 Hz, 1H), 3.48 (ddd, *J* = 15.5, 9.8, 6.5 Hz, 1H), 3.37–3.25 (m, 1H), 3.04 (s, 3H), 2.51 (dd, *J* = 13.7, 4.8 Hz, 1H), 2.31 (dd, *J* = 13.8, 8.7 Hz, 1H), 1.96 (s, 1H), 1.79–1.66 (m, 1H), 1.71 (s, 3H), 1.55 (s, 9H), 1.46 (s, 9H), 0.94 (s, 9H); ^{13}C NMR (125 MHz, CD_3OD) δ 173.2, 172.8, 172.7, 168.3, 165.3, 158.2, 157.9, 157.7, 157.1, 147.2, 143.7, 135.2, 130.7, 129.8, 126.6, 120.3, 99.5, 99.0, 84.1, 81.7, 80.6, 77.7, 76.8, 69.4, 49.0, 47.6, 46.5, 43.0, 39.1, 36.6, 32.0, 28.7, 28.4, 26.6, 22.0; HRMS (ESI) calcd for $C_{43}H_{64}N_{11}O_{11}$ $[M+H]^+$ 910.4781, found 910.4757.

(2*S*,3*S*,6*R*)-3-((*S*)-3-Amino-5-(1-methylguanidino)pentanamido)-6-(4-(4-aminobenzamido)-2-oxopyrimidin-1(2*H*)-yl)-3,6-dihydro-2*H*-pyran-2-carboxamide (29) tri-TFA salt

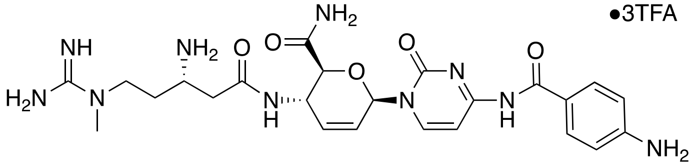

Compound **25** (62.7 mg, 0.0846 mmol) was dissolved in 50% (v/v) TFA/CH_2Cl_2 (2.5 mL) while cooling (ice bath). After 2 h, the solution was concentrated and the resulting residue was diluted with benzene (4 mL) and concentrated under vacuum. The benzene treatment was repeated two more times and the resulting residue dried under vacuum overnight. The residue was purified using

automated flash chromatography (C18, 30g, 0-50% B, solvents modified w/ 0.1% TFA) to yield the tri-TFA salt of **29** (45.6 mg, 61%) as a glassy yellow/orange solid: $[\alpha]_D^{25}$ = +69° (*c* 2.3, CH_3OH); ^1H NMR (500 MHz, CD_3OD) δ 8.06 (d, *J* = 7.6 Hz, 1H), 7.83–7.78 (m, 2H), 7.53 (d, *J* = 7.7 Hz, 1H), 6.79–6.73 (m, 2H), 6.70 (dt, *J* = 3.3, 1.8 Hz, 1H), 6.11 (dt, *J* = 10.2, 2.0 Hz, 1H), 5.95 (ddd, *J* = 10.3, 2.6, 1.5 Hz, 1H), 4.93 (dq, *J* = 9.5, 2.6 Hz, 1H), 4.25 (d, *J* = 9.5 Hz, 1H), 3.67–3.40 (m, 3H), 3.08 (s, 3H), 2.71 (dd, *J* = 15.8, 4.7 Hz, 1H), 2.58 (dd, *J* = 15.8, 7.2 Hz, 1H), 2.15–1.96 (m, 2H); ^{13}C NMR (125 MHz, CD_3OD) δ 173.1, 171.4, 168.7, 164.4, 158.3, 155.2, 153.0, 148.1, 135.2, 131.8, 126.7, 122.1, 115.9, 98.9, 81.8, 77.5, 48.3, 48.0, 46.6, 38.1, 36.6, 30.6; HRMS (ESI) calcd for $C_{24}H_{33}N_{10}O_5$ $[M+H]^+$ 541.2630, found 541.2635.

(2*S*,3*S*,6*R*)-3-((*S*)-3-Amino-5-(1-methylguanidino)pentanamido)-6-(4-(4-(methylamino)benzamido)-2-oxopyrimidin-1(2*H*)-yl)-3,6-dihydro-2*H*-pyran-2-carboxamide (30) tri-TFA salt

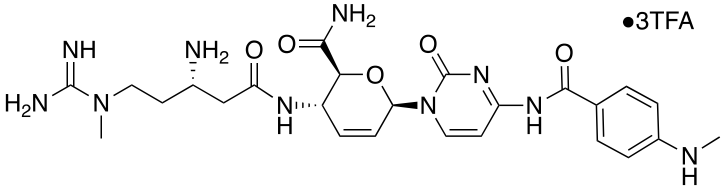

Compound **26** (38.6 mg, 0.0511 mmol) was dissolved in 50% (v/v) TFA/CH_2Cl_2 (1.5 mL) while cooling (ice bath). After 2 h, the solution was concentrated and the resulting residue was diluted with benzene (4 mL) and concentrated. The benzene treatment was repeated two more times and the residue dried under vacuum overnight. The residue was purified using automated flash chromatography (C18, 30 g, 0-50% B, solvents modified w/ 0.1% TFA) to yield the tri-TFA salt of **30** (28.2 mg, 62%) as a glassy, orange solid: $[\alpha]_D^{23}$ = +55° (*c* 1.4, CH_3OH); ^1H NMR (400 MHz, CD_3OD) δ 8.09 (d, *J* = 7.4 Hz, 1H), 7.88–7.81 (m, 2H), 7.48 (s, 1H), 6.70 (dt, *J* = 3.4, 1.8 Hz, 1H), 6.68–6.60 (m, 2H), 6.12 (d, *J* = 10.6 Hz, 1H), 5.95 (ddd, *J* = 10.3, 2.7, 1.5 Hz, 1H), 4.93 (dq, *J* =

9.5, 2.6 Hz, 1H), 4.25 (dd, *J* = 9.5, 1.0 Hz, 1H), 3.68–3.38 (m, 3H), 3.08 (s, 3H), 2.86 (s, 3H), 2.71 (dd, *J* = 15.8, 4.7 Hz, 1H), 2.58 (dd, *J* = 15.8, 7.1 Hz, 1H), 2.13–1.97 (m, 2H); ^{13}C NMR (125 MHz, CD_3OD) δ 173.1, 171.3, 168.8, 164.3, 158.3, 156.0, 154.8, 148.1, 135.2, 131.8, 126.7, 119.4, 112.3, 98.8, 81.8, 77.5, 48.3, 48.0, 46.6, 38.1, 36.6, 30.6, 29.9; HRMS (ESI) calcd for $C_{25}H_{35}N_{10}O_5$ $[M+H]^+$ 555.2786, found 555.2798.

(2*S*,3*S*,6*R*)-6-(4-(4-Acetamidobenzamido)-2-oxopyrimidin-1(2*H*)-yl)-3-((*S*)-3-amino-5-(1-methylguanidino)pentanamido)-3,6-dihydro-2*H*-pyran-2-carboxamide (31) di-TFA salt

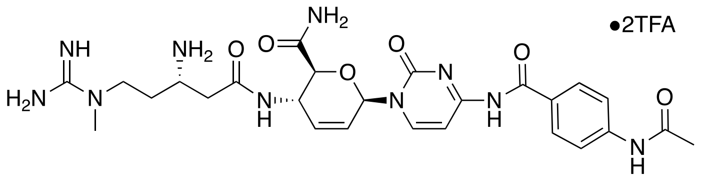

Compound **27** (36.2 mg, 0.0530 mmol) was dissolved in 50% (v/v) TFA/CH_2Cl_2 (1.5 mL) while cooling (ice bath). After 2 h, the mixture was concentrated and the resulting residue was diluted with benzene (4 mL) and concentrated. The benzene treatment was repeated two more times and the residue dried under vacuum overnight. The residue was purified using automated flash chromatography (C18, 30g, 0-50% B, solvents modified w/ 0.1% TFA) to yield the di-TFA salt of **31** (26.1 mg, 63%) as a glassy, off-white solid: $[\alpha]_D^{23}$ = +63° (*c* 1.3, CH_3OH); ^{1}H NMR (400 MHz, CD_3OD) δ 8.08 (dd, *J* = 7.6, 0.9 Hz, 1H), 8.01–7.94 (m, 2H), 7.79–7.72 (m, 2H), 7.59 (d, *J* = 7.5 Hz, 1H), 6.71 (dt, *J* = 3.4, 1.7 Hz, 1H), 6.12 (dt, *J* = 10.2, 2.0 Hz, 1H), 5.95 (ddd, *J* = 10.2, 2.6, 1.5 Hz, 1H), 4.96–4.93 (m, 1H), 4.26 (d, *J* = 9.4 Hz, 1H), 3.68–3.39 (m, 3H), 3.08 (s, 3H), 2.71 (dd, *J* = 15.8, 4.8 Hz, 1H), 2.59 (dd, *J* = 15.8, 7.2 Hz, 1H), 2.17 (s, 3H), 2.11–1.97 (m, 2H); ^{13}C NMR (125 MHz, CD_3OD) δ 173.1, 172.1, 171.3, 168.5, 165.0, 158.3, 156.9, 147.7, 144.8, 135.0, 130.5,

128.9, 126.9, 120.2, 99.4, 81.8, 77.6, 48.3, 48.0, 46.6, 38.1, 36.6, 30.6, 24.1; HRMS (ESI) calcd for $C_{26}H_{35}N_{10}O_6$ $[M+H]^+$ 583.2736, found 583.2748.

(2*S*,3*S*,6*R*)-6-(4-(4-((*S*)-2-Amino-3-hydroxy-2-methylpropanamido)benzamido)-2-oxopyrimidin-1(2*H*)-yl)-3-((*S*)-3-amino-5-(1-methylguanidino)pentanamido)-3,6-dihydro-2*H*-pyran-2-carboxamide (32) tri-TFA salt

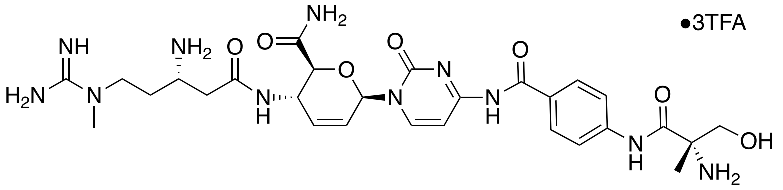

Compound **28** (93.9 mg, 0.103 mmol) was dissolved in 50% (v/v) TFA/CH_2Cl_2 (2.5 mL) while cooling (ice bath). After 2 h, the mixture was concentrated and the resulting residue was diluted with benzene (4 mL) and concentrated. The benzene treatment was repeated two more times and the resulting residue dried under vacuum overnight. The residue was purified using automated flash chromatography (C18, 30g, 0-30% B, solvents modified w/ 0.1% TFA) to yield the tri-TFA salt of **32** (81.9 mg, 81%) as a glassy, off-white solid: $[\alpha]_D^{23}$ = +63° (*c* 4.1, CH_3OH); ^1H NMR (400 MHz, CD_3OD) δ 8.08 (d, *J* = 7.5 Hz, 1H), 8.03–7.99 (m, 2H), 7.87–7.83 (m, 2H), 7.64 (d, *J* = 7.6 Hz, 1H), 6.71 (dt, *J* = 3.4, 1.8 Hz, 1H), 6.11 (dt, *J* = 10.2, 2.1 Hz, 1H), 5.95 (ddd, *J* = 10.2, 2.5, 1.6 Hz, 1H), 4.95–4.93 (m, 1H), 4.25 (d, *J* = 9.5 Hz, 1H), 4.09 (d, *J* = 11.8 Hz, 1H), 3.82 (d, *J* = 11.9 Hz, 1H), 3.69–3.50 (m, 2H), 3.49–3.39 (m, 1H), 3.08 (s, 3H), 2.71 (dd, *J* = 15.8, 4.7 Hz, 1H), 2.58 (dd, *J* = 15.4, 7.5 Hz, 1H), 2.14–1.96 (m, 2H), 1.64 (s, 3H); ^{13}C NMR (125 MHz, CD_3OD) δ 173.1, 171.4, 170.2, 168.3, 165.2, 158.3, 157.5, 147.4, 143.8, 134.9, 130.4, 130.1, 127.0, 121.3, 99.5, 81.8, 77.6, 65.5, 63.6, 48.3, 48.0, 46.6, 38.2, 36.6, 30.6, 18.8; HRMS (ESI) calcd for $C_{28}H_{40}N_{11}O_7$ $[M+H]^+$ 642.3107, found 642.3116.

***tert*-Butyl (2*R*,4*S*)-4-((4-((1-((2*R*,5*S*,6*S*)-5-((*S*)-3-((*tert*-butoxycarbonyl)amino)-5-(1-methylguanidino)pentanamido)-6-(phenethylcarbamoyl)-5,6-dihydro-2*H*-pyran-2-yl)-2-oxo-1,2-dihydropyrimidin-4-yl)carbamoyl)phenyl)carbamoyl)-2-(*tert*-butyl)-4-methyloxazolidine-3-carboxylate (36)**

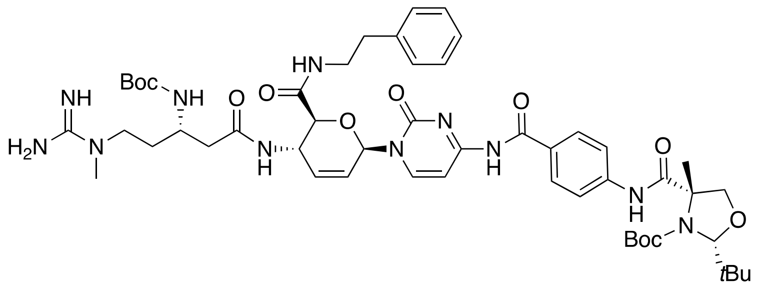

PABA-oxazolidine **12** (118 mg, 0.289 mmol), HATU (97.2 mg, 0.256 mmol), and DIPEA (66.7 µL, 0.383 mmol) were dissolved in anhydrous DMF (0.7 mL) and stirred for 5 min at rt. Compound **24** (75.9 mg, 0.109 mmol) dissolved in anhydrous DMF (0.82 mL) was added and the reaction mixture was heated (oil bath, 70 °C). After 2 h, the mixture was concentrated and the resulting residue was dissolved in methanol and adsorbed onto Celite. Solid-loaded automated flash chromatography (C18, 30 g, 0-100% B, solvents modified w/ 0.1% formic acid) yielded **36** (60.6 mg, 55%) as a glassy white solid: $[\alpha]_D^{24}$ = +91° (*c* 2.6, CH_3OH); ^1H NMR (500 MHz, CD_3OD) δ 8.05–7.97 (m, 3H), 7.76–7.69 (m, 2H), 7.62 (d, *J* = 6.7 Hz, 1H), 7.29–7.12 (m, 5H), 6.66 (dt, *J* = 3.4, 1.8 Hz, 1H), 6.13 (dt, *J* = 10.2, 2.1 Hz, 1H), 5.92 (ddd, *J* = 10.3, 2.6, 1.5 Hz, 1H), 5.19 (s, 1H), 4.79 (dq, *J* = 9.2, 2.8 Hz, 1H), 4.70 (d, *J* = 8.9 Hz, 1H), 4.25 (d, *J* = 9.2 Hz, 1H), 3.90–3.83 (m, 1H), 3.81 (d, *J* = 8.8 Hz, 1H), 3.51–3.31 (m, 4H), 3.04 (s, 3H), 2.79 (t, *J* = 7.4 Hz, 2H), 2.49 (dd, *J* = 14.0, 5.2 Hz, 1H), 2.36 (dd, *J* = 13.9, 7.9 Hz, 1H), 2.02–1.91 (m, 1H), 1.85–1.73 (m, 1H), 1.71 (s, 3H), 1.55 (s, 9H), 1.45 (s, 9H), 0.94 (s, 9H); ^{13}C NMR (125 MHz, CD_3OD) δ 173.2, 172.6, 170.0, 168.3, 165.3, 158.2, 157.84, 157.80, 157.1, 147.2, 143.8, 140.3, 135.2, 130.7, 129.85, 129.81, 129.5, 127.4, 126.5, 120.3, 99.5, 99.0, 84.1, 81.7, 80.5, 78.1, 76.8, 69.4, 49.0, 47.4, 46.7,

42.7, 41.8, 39.1, 36.5, 36.3, 32.1, 28.7, 28.4, 26.6, 22.0; HRMS (ESI) calcd for $C_{51}H_{72}N_{11}O_{11}$ $[M+H]^+$ 1014.5407, found 1014.5401.

(2*S*,3*S*,6*R*)-3-((*S*)-3-Amino-5-(1-methylguanidino)pentanamido)-6-(4-(4-aminobenzamido)-2-oxopyrimidin-1(2*H*)-yl)-*N*-phenethyl-3,6-dihydro-2*H*-pyran-2-carboxamide (37) tri-TFA salt

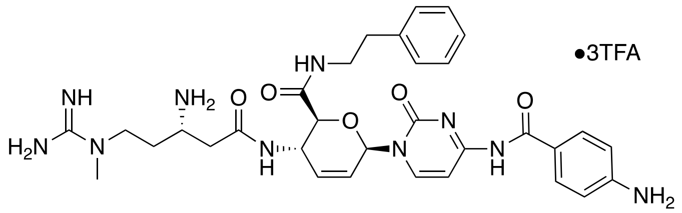

N-Boc-PABA **9** (52.4 mg, 0.221 mmol), HATU (84.8 mg, 0.223 mmol), and DIPEA (56.8 μL, 0.326 mmol) were dissolved in anhydrous DMF (0.72 mL) and stirred at rt for 10 min. Boc BLS phenethyl amide dihydrochloride **24** (76.0 mg, 0.109 mmol) dissolved in anhydrous DMF (0.8 mL) was added and the mixture was heated (oil bath, 70 °C). After 2.5 h, the mixture was concentrated to an orange residue, which was dissolved in CH_3OH and adsorbed onto Celite. Solid-loaded automated flash chromatography (C18, 30 g, 0-100% B, solvents modified w/ 0.1% formic acid) yielded a mixture of **33** and **9** as a yellow, amorphous solid, which was used in the next reaction without additional purification.

The mixture of compound **33** and **9** was dissolved in 50% (v/v) TFA/CH_2Cl_2 (2.4 mL) while cooling (ice bath). After 4 h, the mixture was concentrated the resulting residue was diluted with benzene (4 mL) and concentrated. The benzene treatment was repeated two more times and the resulting residue dried under vacuum overnight. The residue was purified using automated flash chromatography (C18, 30 g, 0-60% B, solvents modified w/ 0.1% TFA) to yield the tri-TFA salt of **37** (31.2 mg, 29%, 2 steps) as a glassy, yellow solid: $[\alpha]_D^{24}$ = +46° (*c* 1.2, CH_3OH); ^1H NMR

(400 MHz, CD_3OD) δ 7.96 (d, *J* = 7.6 Hz, 1H), 7.83–7.76 (m, 2H), 7.58 (d, *J* = 7.5 Hz, 1H), 7.30–7.13 (m, 5H), 6.77–6.69 (m, 2H), 6.69 (dt, *J* = 3.4, 1.8 Hz, 1H), 6.11 (dt, *J* = 10.2, 2.0 Hz, 1H), 5.94 (ddd, *J* = 10.3, 2.6, 1.5 Hz, 1H), 4.91–4.89 (m, 1H), 4.23 (d, *J* = 9.4 Hz, 1H), 3.68–3.44 (m, 3H), 3.39 (dd, *J* = 8.3, 6.5 Hz, 2H), 3.08 (s, 3H), 2.78 (t, *J* = 7.5 Hz, 2H), 2.72 (dd, *J* = 15.8, 4.5 Hz, 1H), 2.58 (dd, *J* = 15.8, 7.4 Hz, 1H), 2.15–1.98 (m, 2H); ^{13}C NMR (125 MHz, CD_3OD) δ 171.4, 170.3, 168.7, 165.2, 158.3, 157.0, 154.4, 147.2, 140.3, 134.9, 131.6, 129.8, 129.6, 127.5, 126.9, 121.4, 115.0, 99.3, 81.7, 77.7, 48.4, 48.0, 46.8, 41.8, 38.1, 36.6, 36.3, 30.7; HRMS (ESI) calcd for $C_{32}H_{41}N_{10}O_5$ $[M+H]^+$ 645.3256, found 645.3254.

(2*S*,3*S*,6*R*)-3-((*S*)-3-Amino-5-(1-methylguanidino)pentanamido)-6-(4-(4-(methylamino)benzamido)-2-oxopyrimidin-1(2*H*)-yl)-*N*-phenethyl-3,6-dihydro-2*H*-pyran-2-carboxamide (38) tri-TFA salt

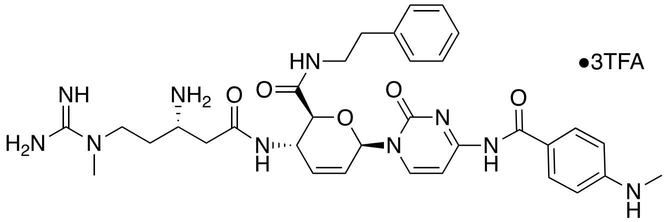

N-Boc-*N*-methyl-PABA **10** (59.7 mg, 0.238 mmol), HATU (84.8 mg, 0.223 mmol), and DIPEA (56.9 µL, 0.327 mmol) were dissolved in anhydrous DMF (0.72 mL) and stirred at rt for 20 min. Compound **24** (76.1 mg, 0.109 mmol) dissolved in anhydrous DMF (0.8 mL) was added and the mixture was heated (oil bath, 70 °C). After 3 h, the mixture was concentrated and the resulting residue was dissolved in CH_3OH and adsorbed onto Celite. Solid-loaded automated flash chromatography (C18, 30 g, 0-100% B, solvents modified w/ 0.1% formic acid) yielded a mixture of **34** and **10** as a yellow solid, which was used in the next reaction without additional purification.

The mixture of compound **34** and **10** was dissolved in 50% (v/v) TFA/CH_2Cl_2 (4.4 mL) while cooling (ice bath). After 4 h, the mixture was concentrated, and the resulting residue was diluted with benzene (4 mL) and concentrated. The benzene treatment was repeated two more times and the resulting residue dried under vacuum overnight. The residue was purified using automated flash chromatography (C18, 30 g, 0-50% B, solvents modified w/ 0.1% TFA) to yield the tri-TFA salt of **38** (41.9 mg, 38%, 2 steps) as a glassy, yellow solid: $[\alpha]_D^{23}$ = +61° (*c* 2.1, CH_3OH); ^1H NMR (400 MHz, CD_3OD) δ 7.94 (d, *J* = 7.6 Hz, 1H), 7.86–7.80 (m, 2H), 7.61 (d, *J* = 7.3 Hz, 1H), 7.28–7.13 (m, 5H), 6.69–6.67 (m, 1H), 6.67–6.61 (m, 2H), 6.11 (dt, *J* = 10.2, 2.0 Hz, 1H), 5.93 (ddd, *J* = 10.3, 2.7, 1.4 Hz, 1H), 4.91–4.88 (m, 1H), 4.24 (d, *J* = 9.4 Hz, 1H), 3.62–3.43 (m, 3H), 3.39 (dd, *J* = 8.3, 6.5 Hz, 2H), 3.07 (s, 3H), 2.85 (s, 3H), 2.78 (t, *J* = 7.4 Hz, 2H), 2.67 (dd, *J* = 15.4, 4.6 Hz, 1H), 2.55 (dd, *J* = 15.5, 7.5 Hz, 1H), 2.13–1.90 (m, 2H); ^{13}C NMR (125 MHz, CD_3OD) δ 171.7, 170.2, 168.6, 165.6, 158.5, 158.0, 155.8, 146.6, 140.3, 134.8, 131.4, 129.8, 129.6, 127.4, 127.0, 120.0, 112.0, 99.5, 81.7, 77.7, 48.2, 48.1, 46.7, 41.7, 39.5, 36.5, 36.3, 31.3, 29.8; HRMS (ESI) calcd for $C_{33}H_{43}N_{10}O_5$ $[M+H]^+$ 659.3412, found 659.3410.

(2*S*,3*S*,6*R*)-6-(4-(4-Acetamidobenzamido)-2-oxopyrimidin-1(2*H*)-yl)-3-((*S*)-3-amino-5-(1-methylguanidino)pentanamido)-*N*-phenethyl-3,6-dihydro-2*H*-pyran-2-carboxamide (39) di-TFA salt

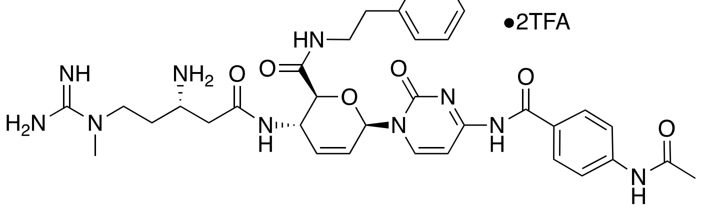

N-Acetyl-PABA **11** (40.9 mg, 0.228 mmol), HATU (85.4 mg, 0.225 mmol), and DIPEA (57.9 μL, 0.332 mmol) were dissolved in anhydrous DMF (0.75 mL) and stirred at rt for 5 min. Compound **24** (77.4 mg, 0.111 mmol) dissolved in anhydrous DMF (0.8 mL) was added and the mixture was heated in an oil bath (70 °C). After 3 h, the mixture was cooled and concentrated. The resulting residue was dissolved in CH_3OH and adsorbed onto Celite. Solid-loaded automated flash chromatography (C18, 30g, 0-100% B, solvents modified w/ 0.1% formic acid) yielded a mixture of **35** and **11** as an off-white solid, which was used in the next reaction without additional purification.

The mixture of **35** and **11** was dissolved in 50% (v/v) TFA/CH_2Cl_2 (4.5 mL) while cooling (ice bath). After 2 h, the solution was concentrated and the resulting residue was diluted with benzene (4 mL) and concentrated. The benzene treatment was repeated two more times and the resulting residue dried under vacuum overnight. The residue was purified using automated flash chromatography (C18, 30 g, 0-50% B, solvents modified w/ 0.1% TFA) to yield **39** (24.3 mg, 22%, 2 steps) as a glassy, off-white solid: $[\alpha]_D^{25}$ = +56° (*c* 1.0, CH_3OH); ^1H NMR (400 MHz, CD_3OD) δ 8.01 (d, *J* = 7.5 Hz, 1H), 7.99–7.96 (m, 2H), 7.76 (d, *J* = 8.8 Hz, 2H), 7.61 (d, *J* = 7.5 Hz, 1H), 7.30–7.12 (m, 5H), 6.70 (dt, *J* = 3.4, 1.8 Hz, 1H), 6.12 (dt, *J* = 10.2, 2.0 Hz, 1H), 5.95 (ddd, *J* = 10.3, 2.6, 1.4 Hz, 1H), 4.93–4.90 (m, 1H), 4.24 (d, *J* = 9.4 Hz, 1H), 3.70–3.43 (m, 3H), 3.39 (dd, *J* = 8.3, 6.5 Hz, 2H), 3.08 (s, 3H), 2.78 (t, *J* = 7.4 Hz, 2H), 2.72 (dd, *J* = 15.7, 4.6 Hz, 1H), 2.59 (dd, *J* = 15.8, 7.5 Hz, 1H), 2.17 (s, 3H), 2.13–1.97 (m, 2H); ^{13}C NMR (125 MHz, CD_3OD) δ 172.0, 171.4, 170.3, 168.4, 165.3, 158.3, 157.5, 147.2, 144.8, 140.3, 134.9, 130.5, 129.8, 129.6, 127.5, 127.0, 120.2, 99.6, 81.8, 77.7, 48.4, 48.0, 46.8, 41.8, 38.1, 36.6, 36.3, 30.7, 28.7, 24.1; HRMS (ESI) calcd for $C_{34}H_{43}N_{10}O_6$ $[M+H]^+$ 687.3362, found 687.3354.

(2*S*,3*S*,6*R*)-6-(4-(4-((*S*)-2-Amino-3-hydroxy-2-methylpropanamido)benzamido)-2-oxopyrimidin-1(2*H*)-yl)-3-((*S*)-3-amino-5-(1-methylguanidino)pentanamido)-*N*-phenethyl-3,6-dihydro-2*H*-pyran-2-carboxamide (40) Tri-TFA Salt

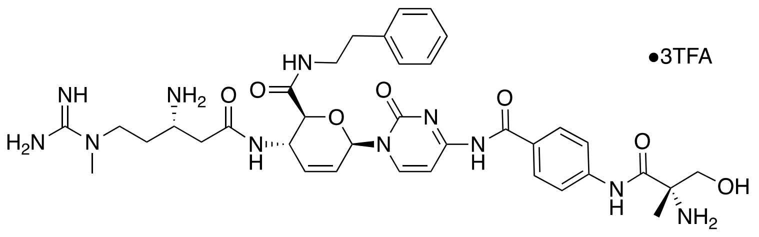

Compound **36** (60.6 mg, 0.0598 mmol) was dissolved in 50% (v/v) TFA/CH_2Cl_2 (1.2 mL) while cooling (ice bath). After 1.5 h, the mixture was concentrated and the resulting material dried under vacuum overnight. The residue was purified using reverse-phase automated flash chromatography (C18, 30 g, 0-50% B, solvents modified w/ 0.1% TFA) to yield the TFA salt of **36** (37.2 mg, 57%) as a glassy off-white solid: $[\alpha]_D^{24}$ = +49° (*c* 1.9, CH_3OH); ^{1}H NMR (400 MHz, CD_3OD) δ 8.05–7.98 (m, 3H), 7.89–7.81 (m, 2H), 7.61 (d, *J* = 7.6 Hz, 1H), 7.33–7.11 (m, 5H), 6.70 (dt, *J* = 3.4, 1.8 Hz, 1H), 6.12 (dt, *J* = 10.2, 2.0 Hz, 1H), 5.95 (ddd, *J* = 10.2, 2.6, 1.5 Hz, 1H), 4.93–4.89 (m, 1H), 4.25 (d, *J* = 9.4 Hz, 1H), 4.10 (d, *J* = 11.8 Hz, 1H), 3.83 (d, *J* = 11.8 Hz, 1H), 3.69–3.45 (m, 3H), 3.43–3.36 (m, 2H), 3.08 (s, 3H), 2.78 (t, *J* = 7.4 Hz, 2H), 2.72 (dd, *J* = 15.7, 4.6 Hz, 1H), 2.60 (dd, *J* = 15.7, 7.6 Hz, 1H), 2.15–1.96 (m, 2H), 1.65 (s, 3H); ^{13}C NMR (125 MHz, CD_3OD) δ 171.4, 170.25, 170.17, 168.4, 165.3, 158.3, 157.6, 147.3, 143.8, 140.3, 134.9, 130.4, 130.1, 129.8, 129.6, 127.5, 126.9, 121.3, 99.5, 81.8, 77.7, 65.4, 63.5, 48.4, 48.0, 46.7, 41.8, 38.2, 36.6, 36.3, 30.7, 18.8; HRMS (ESI) calcd for $C_{36}H_{48}N_{11}O_7$ $[M+H]^+$ 746.3733, found 746.3757.

(2*S*,3*S*,6*R*)-3-((*S*)-3-Amino-5-(1-methylguanidino)pentanamido)-6-(4-(4-aminobenzamido)-2-oxopyrimidin-1(2*H*)-yl)-3,6-dihydro-2*H*-pyran-2-carboxylic acid (43) di-HCl salt

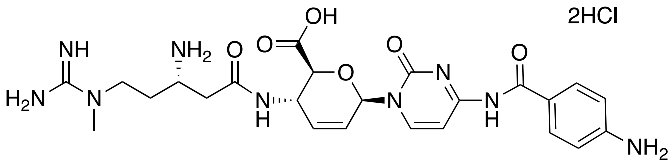

Compound **13** (86.6 mg, 0.115 mmol) was dissolved in 1:1 water/CH_3CN (3.7 mL) and triethylamine (0.160 mL, 1.15 mmol) was added. After 22 h, the mixture was concentrated to a fluffy white solid, which was suspended in 4 N HCl in 1,4-dioxane (1.85 mL). After stirring for 2.5 h, the suspension was diluted with ether (3 mL) and filtered, collecting an orange, gummy precipitate. The precipitate was dissolved in deionized water, acidified with 1 M HCl (aq.) (1 mL), and purified with automated flash chromatography (C18, 30 g, 0-30% B, no modifier) yielding after lyophilization the di-HCl salt of **43** (34.5 mg, 51%, two steps): $[\alpha]_D^{22}$ = +48° (*c* 0.3, CH_3OH); ^{1}H NMR (600 MHz, CD_3OD) δ 8.29 (d, *J* = 7.6 Hz, 1H), 8.01–7.95 (m, 2H), 7.09–7.00 (m, 3H), 6.68 (q, *J* = 2.1 Hz, 1H), 6.28 (ddd, *J* = 10.3, 2.9, 2.1 Hz, 1H), 6.03 (dt, *J* = 10.3, 2.1 Hz, 1H), 4.97 (dq, *J* = 7.8, 2.6 Hz, 1H), 4.48 (d, *J* = 8.0 Hz, 1H), 3.67–3.58 (m, 1H), 3.56–3.46 (m, 2H), 3.09 (s, 3H), 2.76 (dd, *J* = 16.3, 5.0 Hz, 1H), 2.64 (dd, *J* = 16.4, 6.9 Hz, 1H), 2.09–1.97 (m, 2H); ^{13}C NMR (125 MHz, CD_3OD) δ 171.6, 171.5, 169.0, 161.5, 158.2, 152.6, 152.4, 144.1, 135.0, 132.3, 128.0, 125.7, 121.4, 96.9, 81.3, 76.7, 48.1, 47.9, 45.5, 37.4, 36.8, 30.6.; HRMS (ESI) calcd for $C_{24}H_{32}N_9O_6$ $[M+H]^+$ 542.2470, found 542.2468.

(2*S*,3*S*,6*R*)-3-((*S*)-3-Amino-5-(1-methylguanidino)pentanamido)-6-(4-(4-(methylamino)benzamido)-2-oxopyrimidin-1(2*H*)-yl)-3,6-dihydro-2*H*-pyran-2-carboxylic acid (44) di-HCl salt

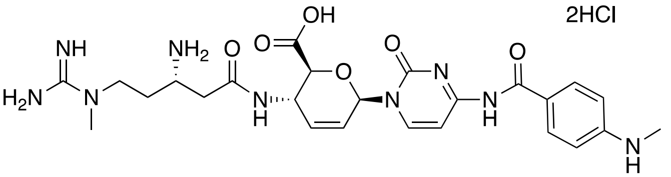

Compound **14** (145.1 mg, 0.1885 mmol) was dissolved in 1:1 water/CH_3CN (6.2 mL) and triethylamine (0.263 mL, 1.89 mmol) was added. After 22 h, the solution was concentrated to yield a fluffy white solid, which was suspended in 4 N HCl in 1,4-dioxane (3.2 mL). After stirring for 2.5 h, the suspension was diluted with ether (3 mL) filtered, collecting an orange, gummy precipitate. The precipitate was dissolved in deionized water, acidified with 1 M HCl (aq.) to pH 2, and purified with automated flash chromatography (C18, 30 g, 0-30% B, no modifier), yielding after lyophilization the di-HCl salt of **44** (57.5 mg, 48%, two steps): $[\alpha]_D^{23}$ = +75° (*c* 0.5 CH_3OH); ^{1}H NMR (600 MHz, D_2O) δ 8.07 (d, *J* = 7.4 Hz, 1H), 7.95–7.90 (m, 2H), 7.31 (d, *J* = 7.5 Hz, 1H), 7.25–7.16 (m, 2H), 6.60 (q, *J* = 2.2 Hz, 1H), 6.23 (dt, *J* = 10.3, 2.4 Hz, 1H), 5.98 (dt, *J* = 10.3, 1.8 Hz, 1H), 4.86 (dq, *J* = 8.2, 2.6 Hz, 1H), 4.42 (d, *J* = 8.4 Hz, 1H), 3.66 (p, *J* = 6.6 Hz, 1H), 3.54–3.39 (m, 2H), 3.03 (s, 3H), 2.97 (s, 3H), 2.78 (dd, *J* = 16.3, 5.1 Hz, 1H), 2.67 (dd, *J* = 16.3, 7.2 Hz, 1H), 2.10–1.98 (m, 2H); ^{13}C NMR (125 MHz, D_2O) δ 172.8, 170.9, 168.3, 165.7, 163.1, 156.4, 155.7, 148.0, 147.2, 132.7, 130.4, 125.1, 116.6, 98.5, 80.1, 75.8, 46.4, 46.3, 44.6, 36.4, 35.7, 32.4, 28.9; HRMS (ESI) calcd for $C_{25}H_{34}N_9O_6$ $[M+H]^+$ 556.2627, found 556.2626.

(2*S*,3*S*,6*R*)-6-(4-(4-Acetamidobenzamido)-2-oxopyrimidin-1(2*H*)-yl)-3-((*S*)-3-amino-5-(1-methylguanidino)pentanamido)-3,6-dihydro-2*H*-pyran-2-carboxylic acid (45) HCl salt

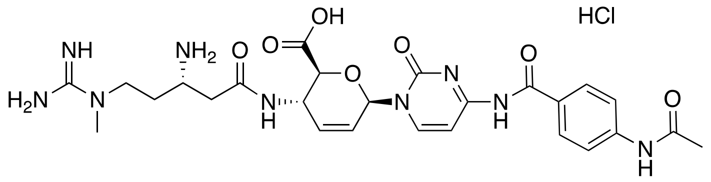

Compound **15** (99.5 mg, 0.143 mmol) was dissolved in 1:1 water/CH_3CN (4.4 mL) and triethylamine (0.200 mL, 1.43 mmol) was added. After 22 h, the solution was concentrated to yield a fluffy off-white solid, which was suspended in 4 N HCl in 1,4-dioxane (2.2 mL). After stirring for 3.5 h, the suspension was diluted with ether (3 mL) and filtered, collecting an off-white precipitate. The precipitate was dissolved in deionized water, acidified with 1 M HCl (aq.) to pH 2, and purified with automated flash chromatography (C18, 30 g, 0-35% B, no modifier), yielding after lyophilization the di-HCl salt of **45** (42.6 mg, 46%, two steps): $[\alpha]_D^{21}$ = +150° (*c* 0.4, 10 mM HCl [aq.]); ^{1}H NMR (600 MHz, D_2O) δ 8.20 (d, *J* = 7.7 Hz, 1H), 7.78–7.74 (m, 2H), 7.46–7.42 (m, 2H), 6.67 (d, *J* = 7.7 Hz, 1H), 6.49 (s, 1H), 6.15–6.09 (m, 1H), 5.82 (ddd, *J* = 10.3, 3.8, 1.9 Hz, 1H), 4.71–4.67 (m, 1H), 4.35 (d, *J* = 8.0 Hz, 1H), 3.52–3.43 (m, 1H), 3.35–3.19 (m, 2H), 2.82 (s, 3H), 2.59 (dd, *J* = 16.5, 4.9 Hz, 1H), 2.49 (dd, *J* = 16.4, 6.9 Hz, 1H), 1.96 (s, 3H), 1.90–1.77 (m, 2H); ^{13}C NMR (125 MHz, D_2O) δ 172.8, 172.6, 170.9, 168.1, 165.6, 163.3, 156.4, 156.3, 147.0, 142.1, 132.6, 129.3, 125.2, 119.9, 98.5, 80.1, 75.6, 46.4, 46.2, 44.6, 36.4, 35.7, 28.9, 23.1; HRMS (ESI) calcd for $C_{26}H_{34}N_9O_7$ $[M+H]^+$ 584.2576, found 584.2582.

(2*S*,3*S*,6*R*)-6-(4-(4-((*S*)-2-Amino-3-hydroxy-2-methylpropanamido)benzamido)-2-oxopyrimidin-1(2*H*)-yl)-3-((*S*)-3-amino-5-(1-methylguanidino)pentanamido)-3,6-dihydro-2*H*-pyran-2-carboxylic acid (46) di-HCl salt

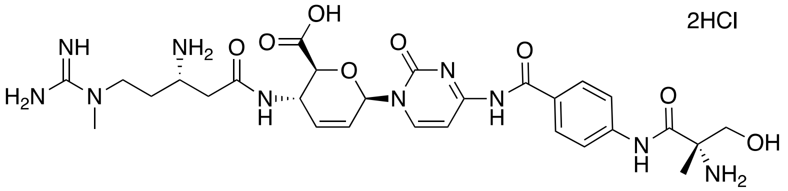

Compound **16** (66.4 mg, 0.0718 mmol) was dissolved in 1:1 water/CH_3CN (2.8 mL) and triethylamine (0.100 mL, 0.718 mmol) was added. After 22 h, the mixture was concentrated to yield a fluffy white solid, which was suspended in 4 N HCl in 1,4-dioxane (1.5 mL). After stirring for 2.5 h, the suspension was diluted with ether (3 mL) and filtered, collecting an off-white precipitate. The precipitate was dissolved in deionized water, acidified with 1 M HCl (aq.) to pH 2, and purified with automated flash chromatography (C18, 30 g, 0-35% B, no modifier), yielding after lyophilization the di-HCl salt of **46** (31.1 mg, 61% over two steps): $[\alpha]_D^{22}$ = +48° (*c* 0.8, 10 mM HCl [aq.]); ^{1}H NMR (600 MHz, D_2O) δ 8.07 (d, *J* = 7.5 Hz, 1H), 7.93–7.90 (m, 2H), 7.66–7.63 (m, 2H), 7.36 (d, *J* = 7.8 Hz, 1H), 6.60 (q, *J* = 2.3 Hz, 1H), 6.23 (dt, *J* = 10.4, 2.8 Hz, 1H), 5.99 (dt, *J* = 10.4, 2.1 Hz, 1H), 4.87 (dq, *J* = 7.7, 2.5 Hz, 1H), 4.44 (d, *J* = 8.3 Hz, 1H), 4.13 (d, *J* = 12.6 Hz, 1H), 3.90 (d, *J* = 12.7 Hz, 1H), 3.67 (p, *J* = 6.6 Hz, 1H), 3.55–3.38 (m, 2H), 3.03 (s, 3H), 2.78 (dd, *J* = 16.3, 5.1 Hz, 1H), 2.68 (dd, *J* = 16.2, 7.1 Hz, 1H), 2.12–1.95 (m, 2H), 1.67 (s, 3H); ^{13}C NMR (125 MHz, D_2O) δ 172.3, 171.1, 169.4, 168.7, 163.4, 156.5, 156.2, 147.4, 141.1, 132.6, 129.4, 129.0, 125.1, 121.6, 98.6, 80.2, 75.3, 64.1, 62.1, 46.5, 46.3, 44.6, 36.4, 35.8, 29.0, 17.8; HRMS (ESI) calcd for $C_{28}H_{39}N_{10}O_8$ $[M+H]^+$ 643.2947, found 643.2970.

Antibacterial Assays

Media and solutions were autoclaved or sterile filtered prior to use and manipulations were carried out in a laminar flow hood. Antibacterial testing was performed in polypropylene 96-well flat bottom plates. The minimal inhibitory concentration of blasticidin S (**1**) hydrochloride, methyl ester (**3**) trihydrochloride, **P10** (**2**) tri-TFA, phenethyl amide (**4**) tri-TFA, and blastimidines M **19**–**22**, A, **29**–**32**, P **37**–**40**, and C **43**–**46** (salt forms as described in the text) were assessed against *Staphylococcus aureus* ΔNorA, *S. aureus* ATCC 6538P, methicillin-resistant *S. aureus* (MRSA) ATCC 43300, *Enterococcus faecalis* ATCC 29212, vancomycin-resistant *Enterococcus*, *Klebsiella pneumoniae* ATCC 29665, *Pseudomonas aeruginosa*, and *Acinetobacter baumannii* ATCC 17978 using serial dilutions. Doxycycline (Gold Bio) as a positive control and the vehicle as a negative control. Stock solutions of each compound were prepared at 2.56 mg/mL in 50% DMSO/water and serially diluted in 50% DMSO/water to create master plates. From the master plates, 10 μL of each dilution was applied to test plates using a Bravo automated liquid transfer system (Agilent). Bacteria previously grown overnight (37 °C, 120–200 RPM), in LB broth were diluted to an OD600 of 0.05 in cation-adjusted Mueller-Hinton broth (CAMHB), grown to an OD600 of ~0.5, and diluted to an OD600 of 0.004 in CAMHB. These diluted cultures were applied to the test plates (190 μL per test well) resulting in final concentrations of the test compounds of 128–1 μg/mL. Growth controls (wells with inoculated media but no vehicle or test compounds) were included to assess any potential toxicity from the vehicle. The plates were incubated at 37 °C for 16–18 h, except for VRE and *E. faecalis*, which were incubated at 37 °C for 24 h. Inhibition was determined by measuring the optical density at 600 nm (OD600) with a Cytation 3 plate reader (BioTek). The optical density measurements were normalized to the positive and negative controls and the vehicle and growth controls were compared and found to be indistinguishable, indicating no toxicity from

the vehicle used. The MIC was the lowest concentration that inhibited >90% of growth. Two biological replicates were performed in duplicate. MIC values are reported as a range from biological replicates.

Table S1. MIC values[a] against reference strains for parent compounds (**Figure 1**) blasticidin S (C1, **1**), P10 (A1, **2**), methyl ester (M1, **3**), phenethyl amide (P1, **4**), and hybrid blastimidines M (**Scheme 2**), A (**Scheme 4**), P (**Scheme 5**) and C (**Scheme 6**).

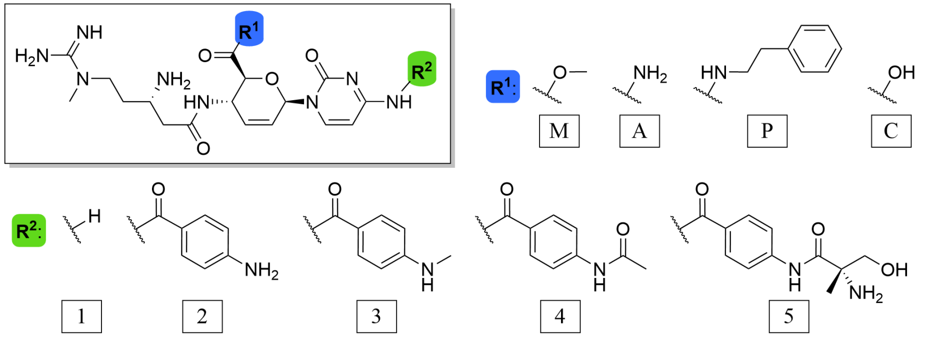

Blastimidine	*S. aureus* ΔNorA	*S. aureus* ATCC 6539P	Methicillin resistant *Staphylococcus epidermidis* (MRSE) ATCC 35984 NRS101	Methicillin resistant *S. aureus* (MRSA) USA300	MRSA ATCC 43300	*E. faecalis* ATCC 29112	Vancomycin resistant *E. faecalis* NR-31972 (VRE)	Vancomycin resistant *Enterococcus faecium* NR-31909 (VRE)	*K. pneumoniae* ATCC- 29665	*P. aeruginosa*	*Escherichia coli* BAA-2452	*A. baumannii* ATCC-17978
**M1 (3)**	64-128	16-32	16	8	16-32	16	32	64	64	64	8	128
**M2 (19)**	16-32	16	16	8	16	8	32	128	64	>128	32	>128
**M3 (20)**	8	8	8	8	16	16	16	64	32	>128	32	>128
**M4 (21)**	32	16-32	64	64	32	32	64	128	32	>128	64	>128
**M5 (22)**	64	64	64	64	64	64	64	16-32	32-64	>128	32	>128
**A1 (P10) (2)**	128	32	32	16	32	32	64	32	32	64	16	32
**A2 (29)**	32	16	32	16	16	16	32	16	16	>128	16	>128
**A3 (30)**	16	8-16	16	8	16	16	32	8-16	32	>128	32	>128
**A4 (31)**	64	64	64	64	64	32	64	32	16-32	>128	32	>128
**A5 (32)**	128	64	128	128	128	64	>128	32	32	>128	32	>128
**P1 (4)**	128	16	4	8-16	32	16	16	128	>128	64-128	>128	>128
**P2 (37)**	8	16-32	16	32	64	8	16	128	>128	128	128	>128
**P3 (38)**	4-8	16-32	8	32	64-128	8-16	16	128	>128	64-128	64	>128
**P4 (39)**	8-16	32	16	64	128	32	32	>128	>128	128	>128	>128
**P5 (40)**	16	16	16	16	32	32	32	16-32	64-128	>128	128	>128
**C1 (BLS) (1)**	>128	>128	>128	128	>128	32	128	128	128	>128	64	>128
**C2 (43)**	>128	>128	>128	128	>128	64	128	64	64	>128	64	>128
**C3 (44)**	>128	>128	>128	128	>128	64	128	64	64-128	>128	>128	>128
**C4 (45)**	>128	>128	>128	>128	>128	>128	>128	>128	128	>128	>128	>128
**C5 (46)**	>128	>128	>128	>128	>128	>128	>128	>128	>128	>128	>128	>128

[a]µg/mL, biological replicates are reported as a range.

Cytotoxicity Assay

The potential cytotoxic effect of the test compounds was investigated using Vero cells (monkey kidney epithelial cells) following established protocols.[3] Briefly, Vero cells were plated into 96-well plates supplemented with Dulbecco's Modified Eagle Medium (DMEM) containing 10% fetal bovine serum (FBS). The cells were placed in a humidified 37 °C environment for incubation with 5% CO_2 for 24 h to allow proper adherence and growth. Following the incubation period, the culture medium was replaced with fresh DMEM containing serial dilutions of the test compounds, while the vehicle alone was used as a negative control. The treated cells were incubated for an additional 24 h. To assess cell viability, the MTS/PMS reagent was added to each well and incubated for 4 h. A microplate reader (Synergy H1, BioTek, USA) was used to measure absorbance at 490 nm. All experiments were conducted using three biological replicates.

Table S2. CC_{50} values[a] (µg/mL) against Vero cells

Series ID	1	2	3	4	5
M	28	126	205	210	150
A	7.0	16	24	68	65
P	235	>256	>256	>256	>256
C	18	66	142	203	177

[a]Values at which the cell viability (normalized to vehicle control) was 50%. Average of three biological replicates.

Time Kill Assay

MRSA USA-300 was grown overnight in cation-adjusted Mueller-Hinton broth (CAMHB), then subcultured into fresh CAMHB and incubated aerobically at 37°C until reaching the logarithmic phase, corresponding to an optical density (OD_{600}) of 0.2. The bacterial cultures were further diluted to adjust to a final concentration of 5×10^5 CFU/mL in CAMHB. P3, P5, vancomycin and linezolid, were added at concentrations of 5× and 10× MIC. A DMSO-treated

culture served as an untreated control. The cultures were aerobically maintained at 37°C in shaking incubator. At specific sampling times, aliquots were collected, resuspended in phosphate-buffered saline (PBS) and distributed to three replicates on tryptic soy agar (TSA). After a 24-hour incubation period at 37°C, the number of colony-forming units (CFUs) was evaluated.

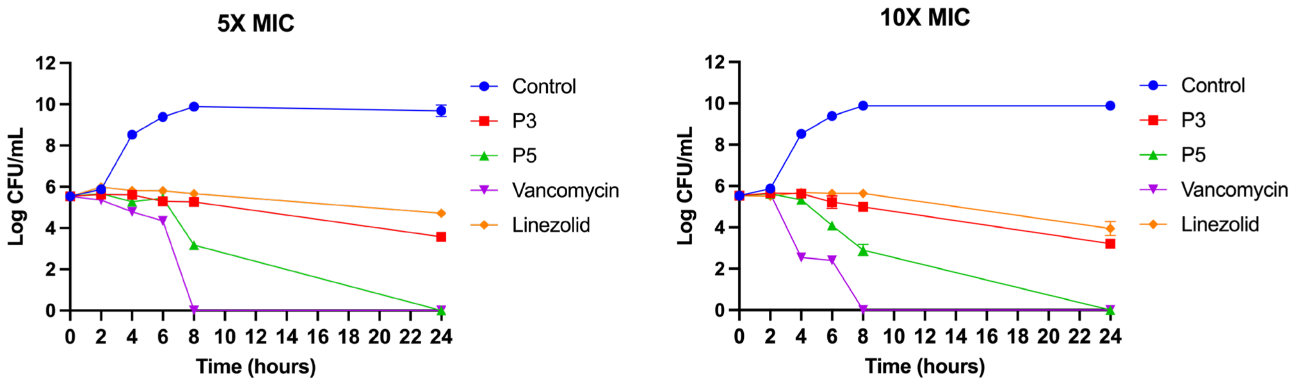

Figure S1. Time kill results against MRSA USA 300 at 5X and 10X MIC. Compound P3 (**38**) is shown as red squares, compound P5 (**40**) is shown as green triangles. Vancomycin (inverted purple triangle) is used as a bactericidal control and linezolid (orange diamond) as a bacteriostatic control with DMSO (blue circles) as a negative control.

Multi-step resistance selection

To assess the ability of MRSA NRS384 (USA300) to develop resistance to P-NM and P-AM, a multi-step serial passage experiment was used, as described previously.[4-6] MRSA strain was serially passaged with sub inhibitory concentrations of compounds and control drugs (Rifampicin and linezolid) in 15 passages over a period of two weeks. Resistance was defined as a 4-fold shift in the MIC.

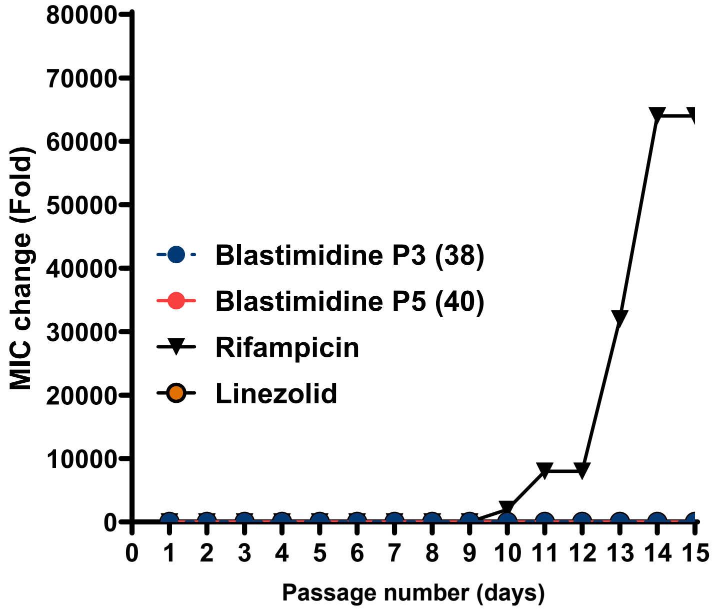

Figure S2. Multi-step resistance selection for blastimidine P3 (38) **and blastimidine P5** (40)**, rifampicin, and linezolid** evaluated against methicillin-resistant *S. aureus* (MRSA) NRS384 (USA300) for 15 days. The minimum inhibitory concentration (MIC) of each test agent was determined after each passage. Data are presented as fold-change in MIC relative to the previous passage.

Structural Comparisons and Computational Modeling

Crystal structures of 50S, 60S, 70S, and 80S ribosomes from *Oryctolagus cuniculus*, *Candida albicans*, *Saccharomyces cerevisiae*, and *Thermus thermophilus*, (PDB ID's: 7q0r,[7] 7nwi,[8] 4u56,[9] 4v9q,[10] 6b4v,[11] and 6czr[12] were aligned to 4v9q in PyMOL[13] for structural comparison. *De novo* models of ribosomal protein L10 from *C. albicans* (PDB ID: 7q0r) and *O. cuniculus* (PDB ID: 7nwi) were generated in AlphaFold3,[14] and the three-dimensional structures were validated using ProSA-web[15,16] and Verify3D.[17] The *de novo* structures were aligned to the crystallized L10 proteins from each respective ribosome in PyMOL to visualize the unresolved regions. Molecular docking of P5 (**40**) to the bacterial ribosome (PDB ID: 6czr) was performed in GNINA,[18] as done previously[1] with box coordinates of (-44X, 126Y, 155Z) and size (30X, 20Y, 30Z). Output poses were overlaid in PyMOL.

Figure S3. ^{1}H NMR of **13** (500 MHz, CD_3OD).

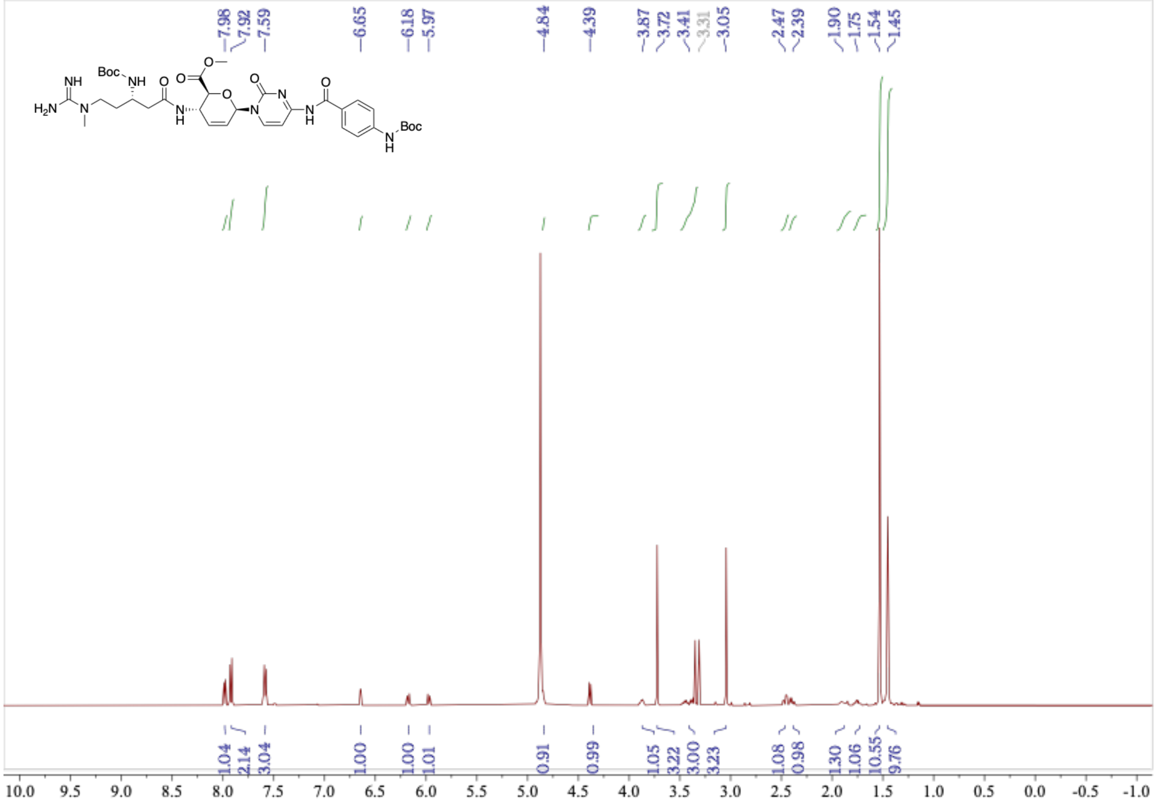

Figure S4. ^{13}C NMR of **13** (125 MHz, CD_3OD).

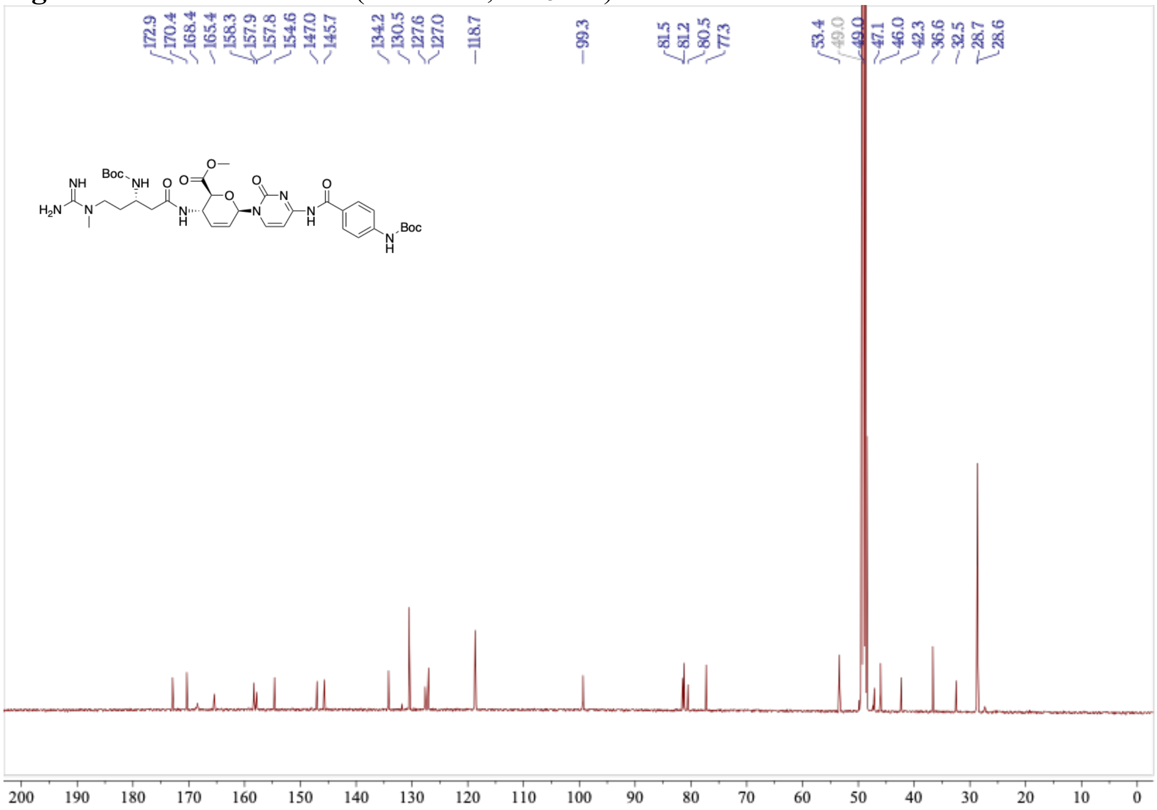

Figure S5. ^{1}H NMR of **14** (500 MHz, CD_3OD).

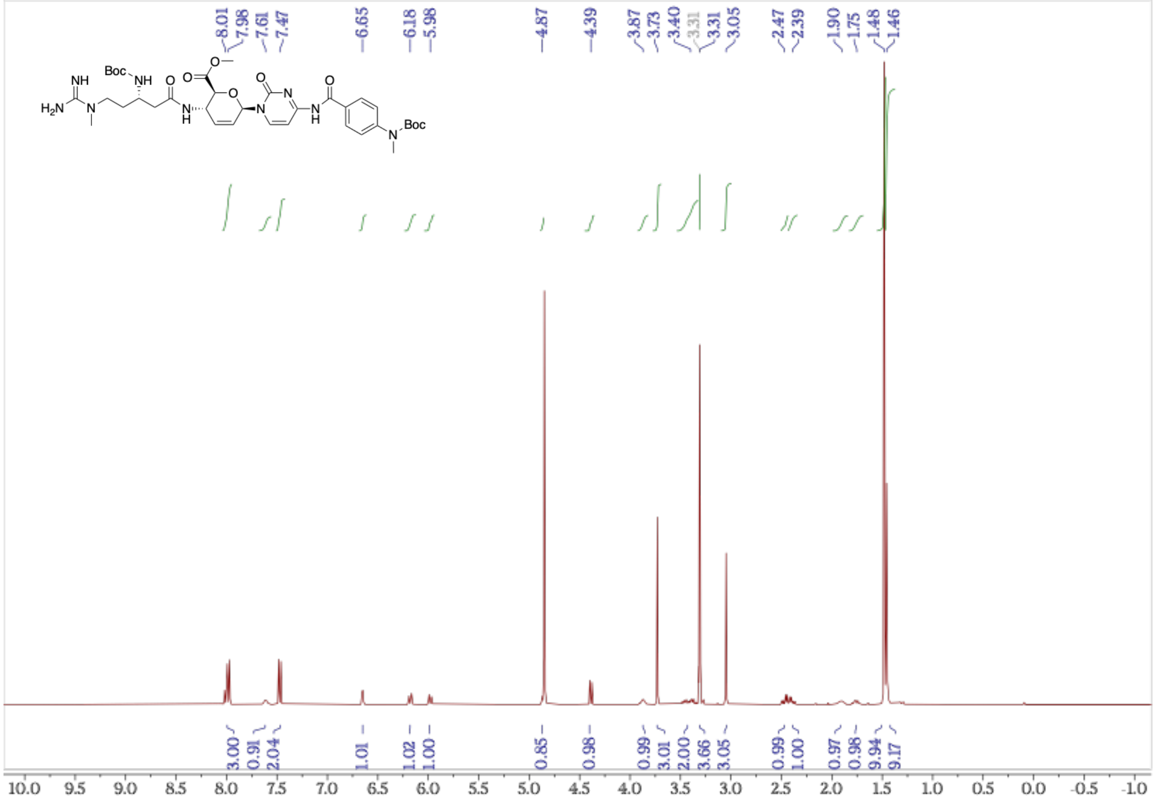

Figure S6. ^{13}C NMR of **14** (125 MHz, CD_3OD).

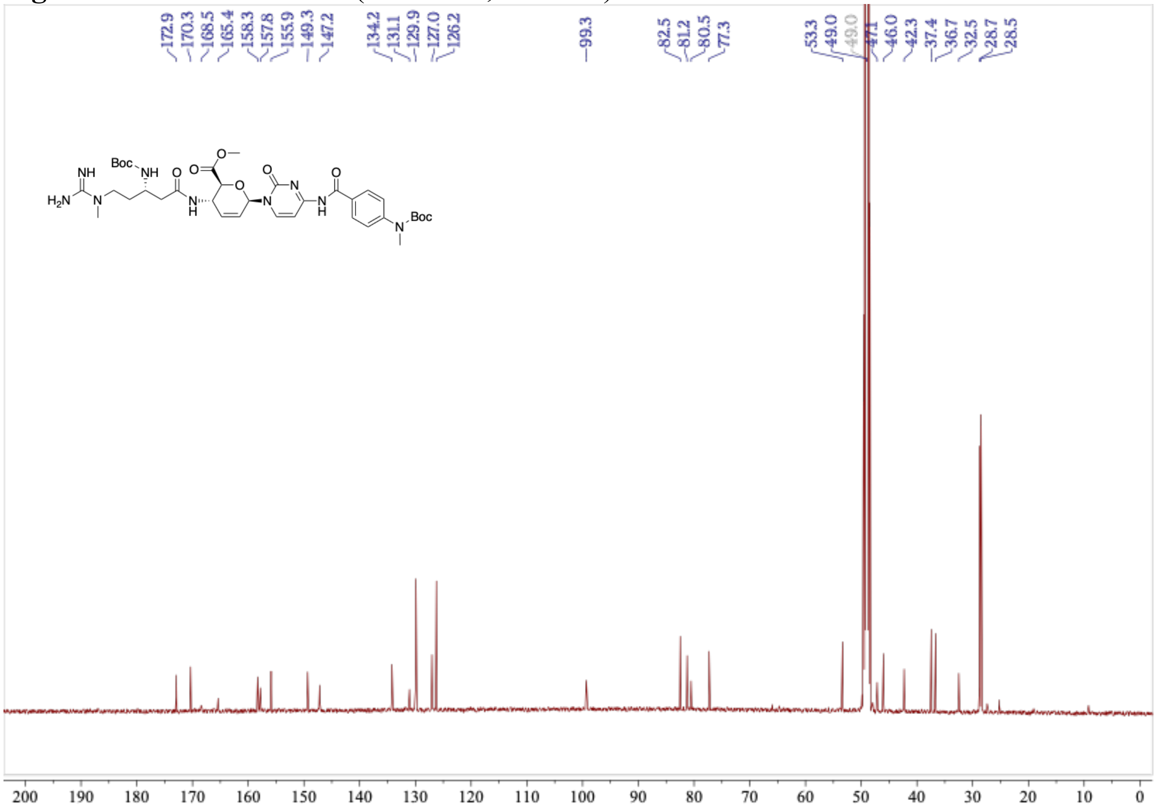

Figure S7. ^{1}H NMR of **16** (400 MHz, CD_3OD).

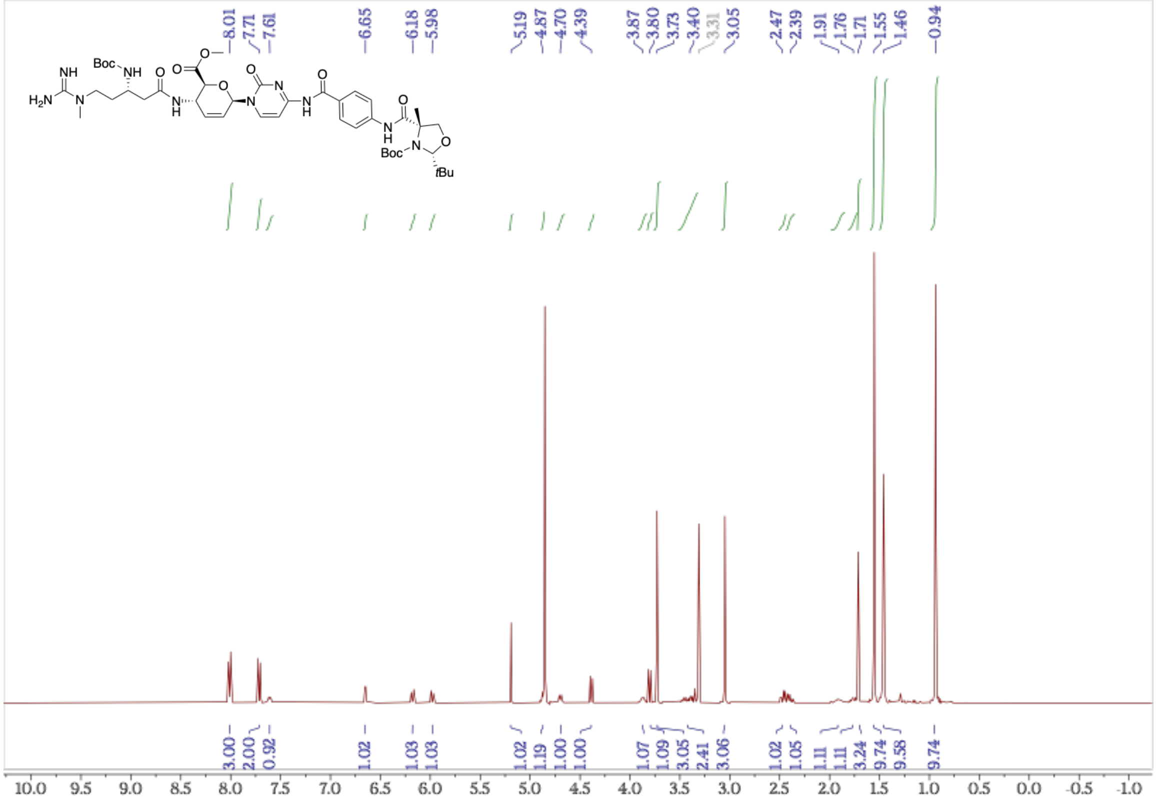

Figure S8. ^{13}C NMR of **16** (125 MHz, CD_3OD).

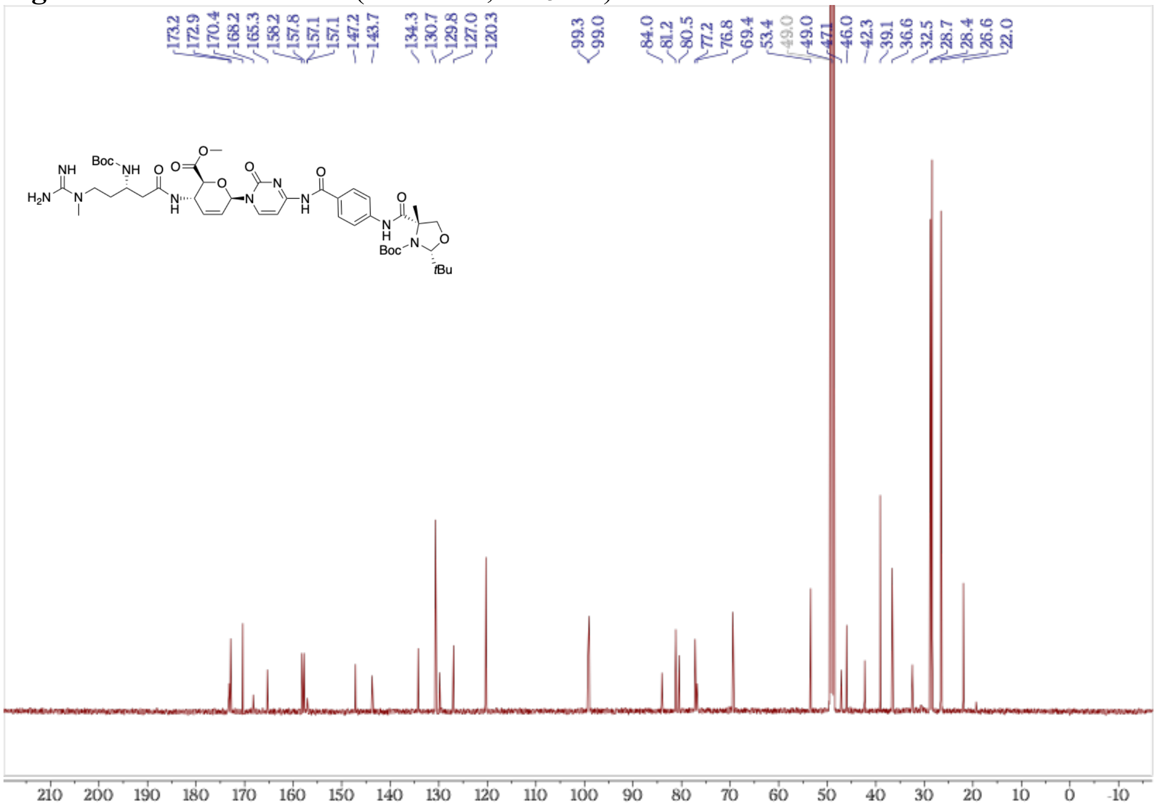

Figure S9. ^{1}H NMR of **19** (500 MHz, CD_3OD).

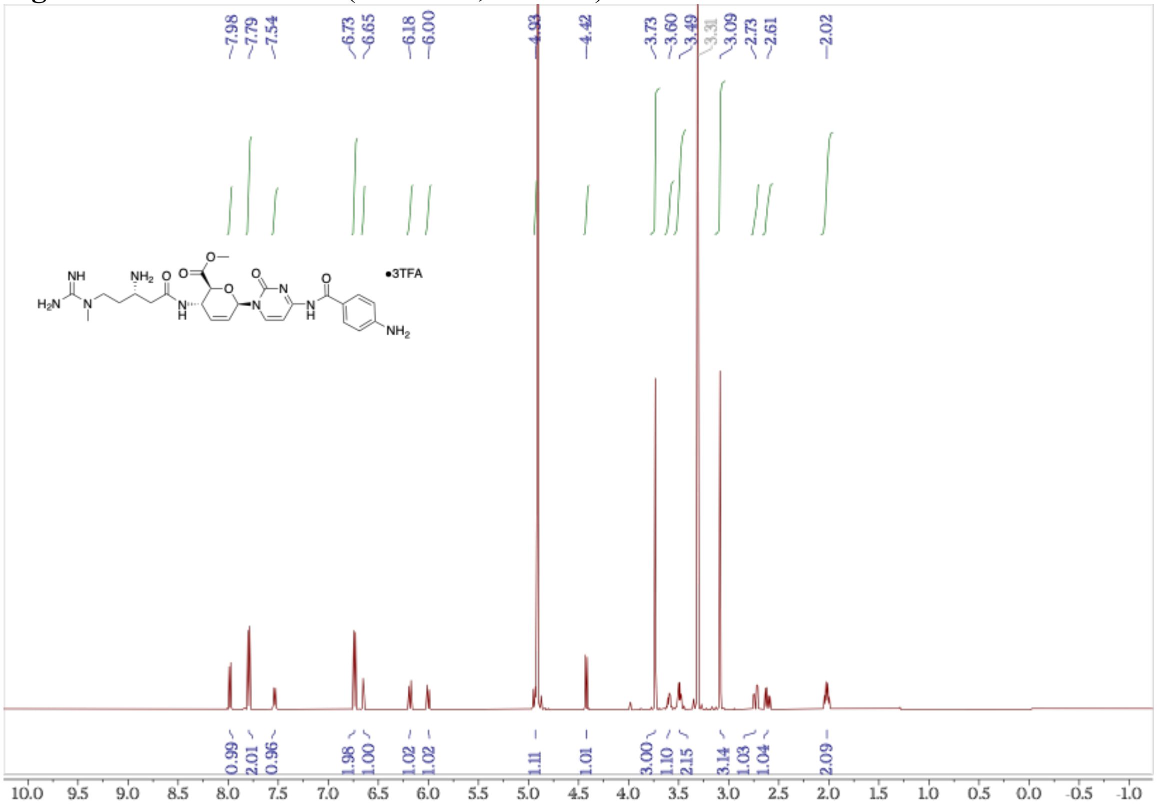

Figure S10. ^{13}C NMR of **19** (125 MHz, CD_3OD).

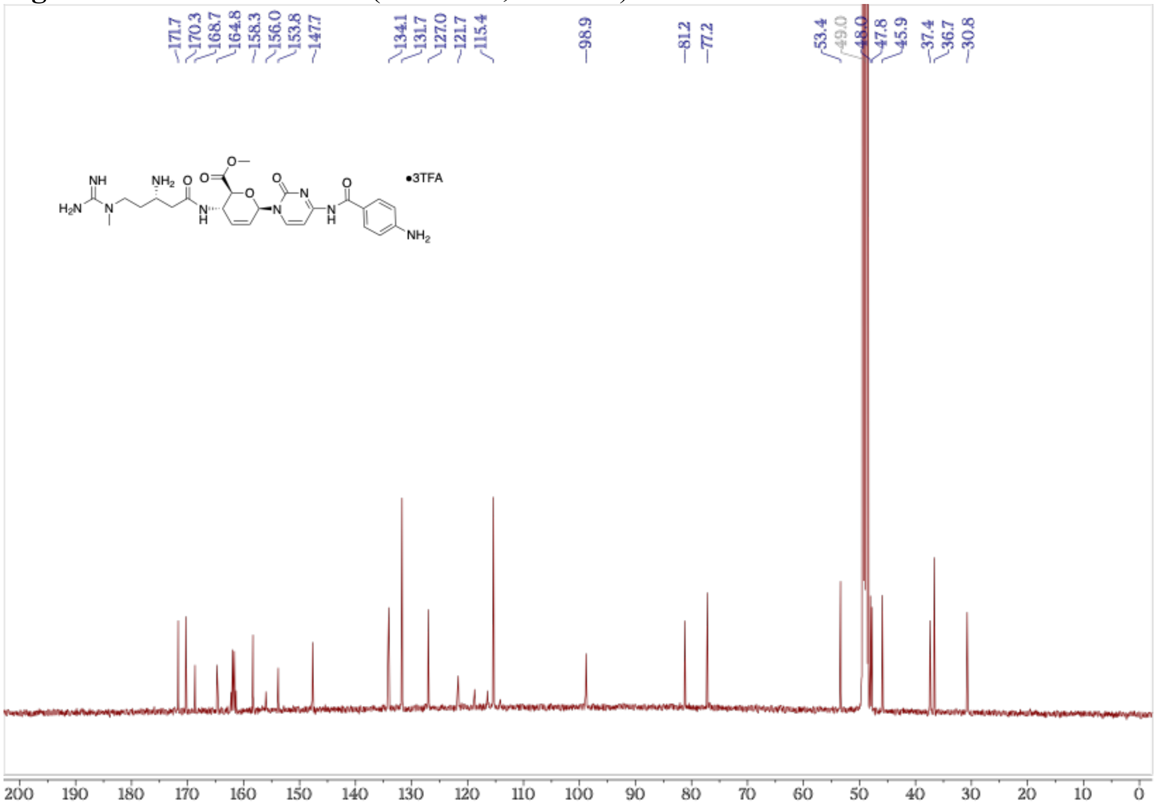

Figure S11. ^{1}H NMR of **20** (500 MHz, CD_3OD).

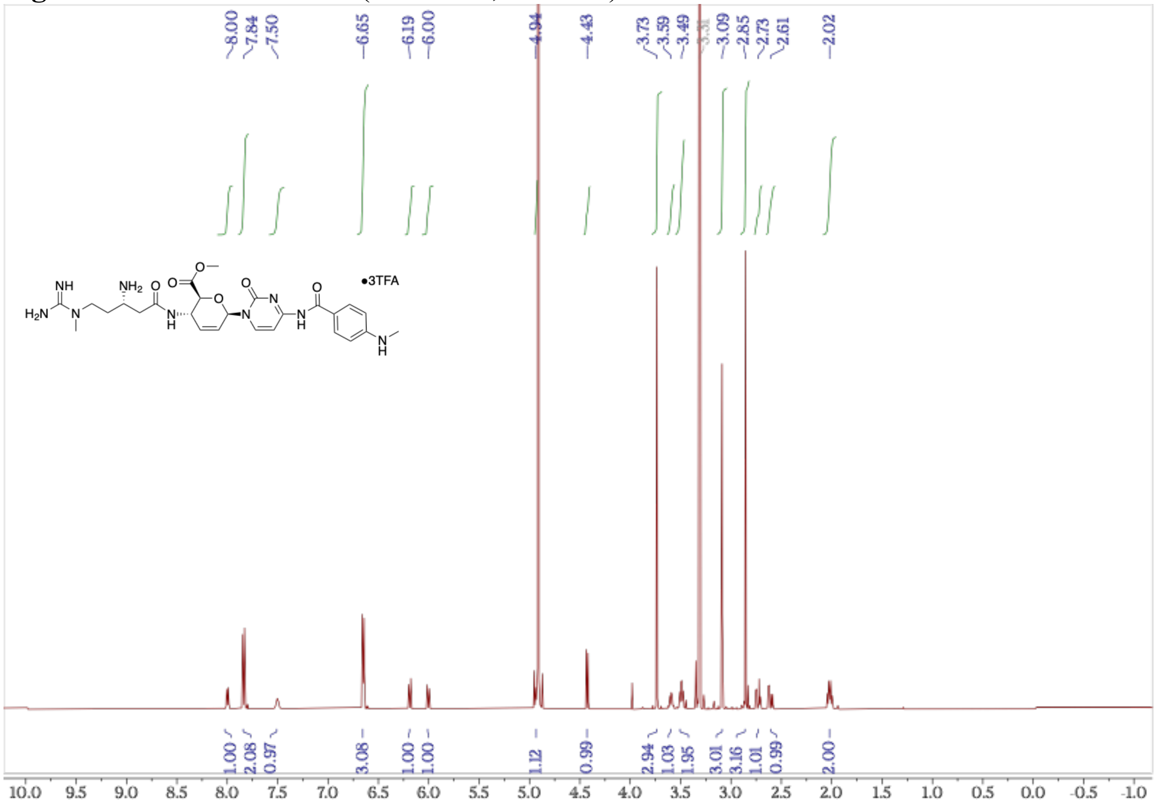

Figure S12. ^{13}C NMR of **20** (125 MHz, CD_3OD).

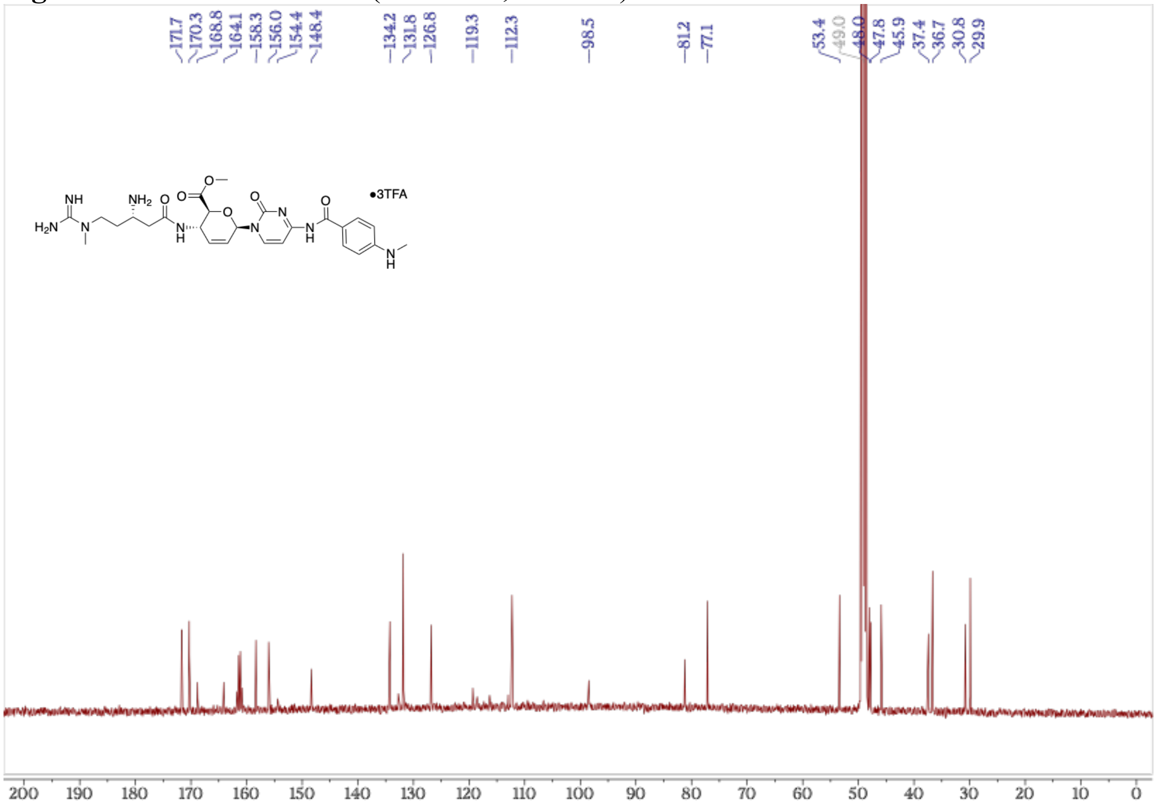

Figure S13. ^{1}H NMR of **21** (500 MHz, CD_3OD)

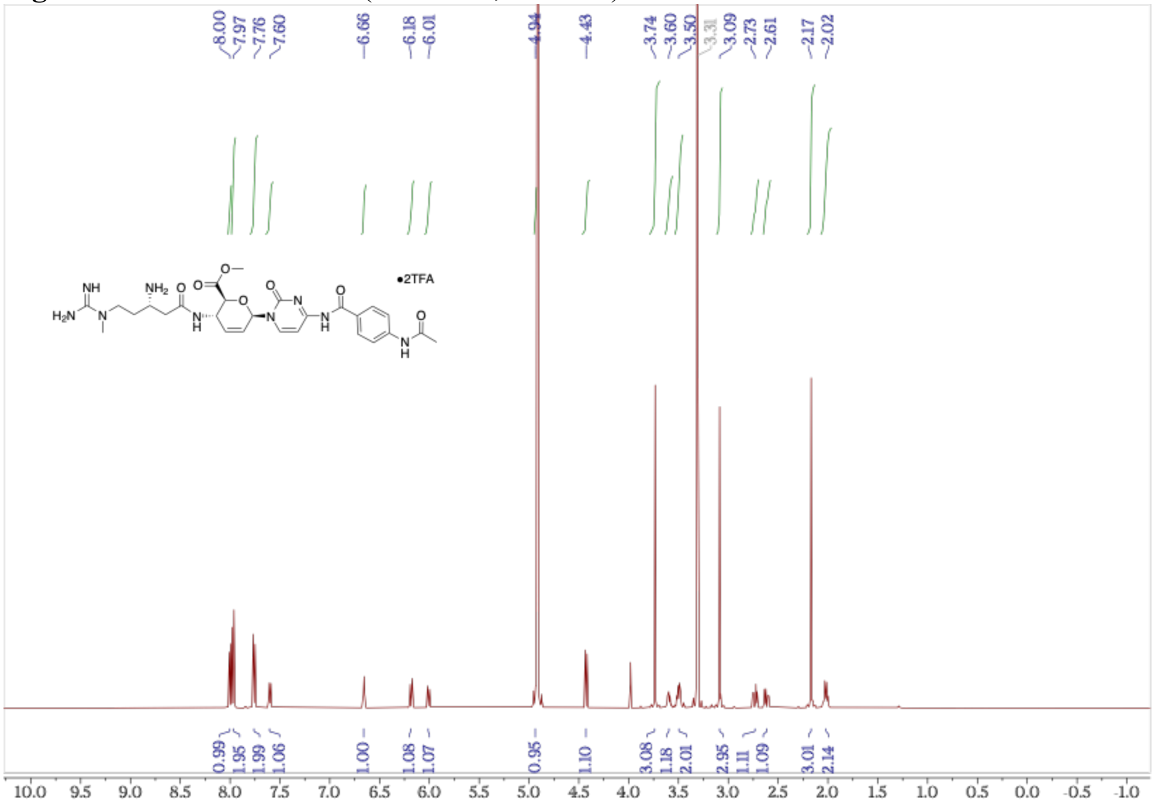

Figure S14. ^{13}C NMR of **21** (125 MHz, CD_3OD).

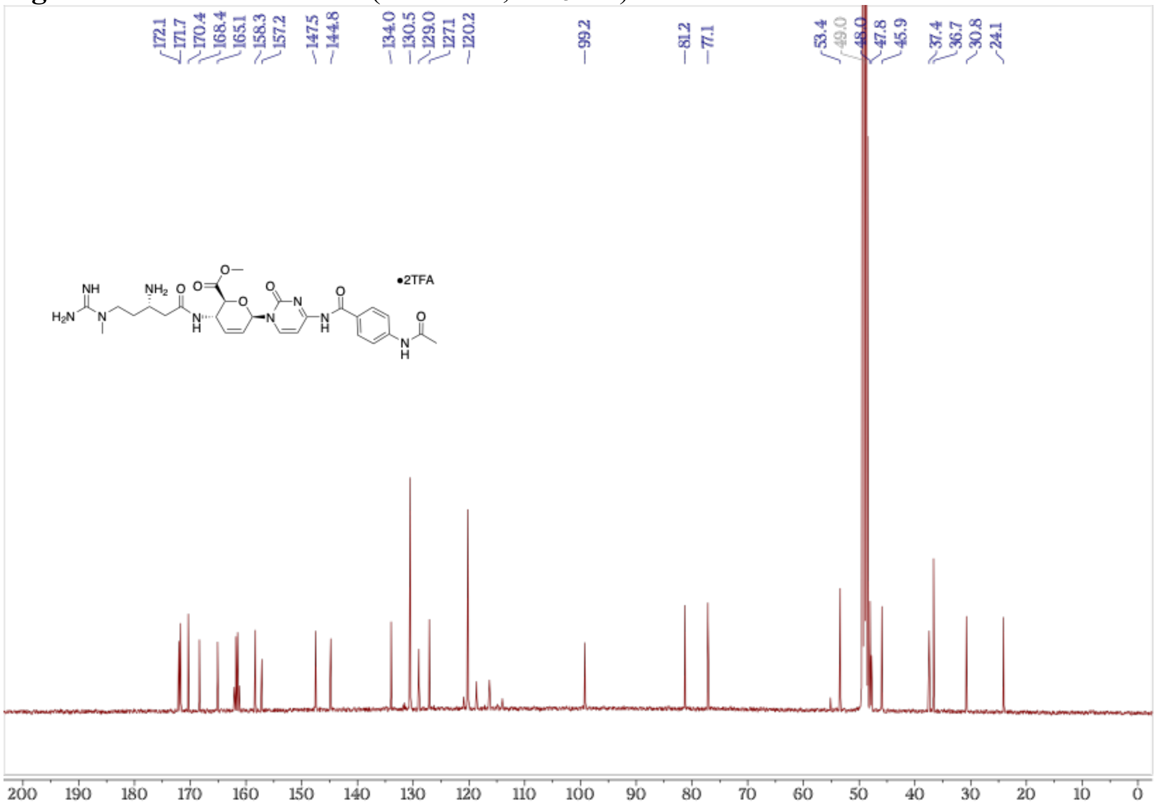

Figure S15. ^{1}H NMR of **22** (400 MHz, CD_3OD) plus CH_3OH.

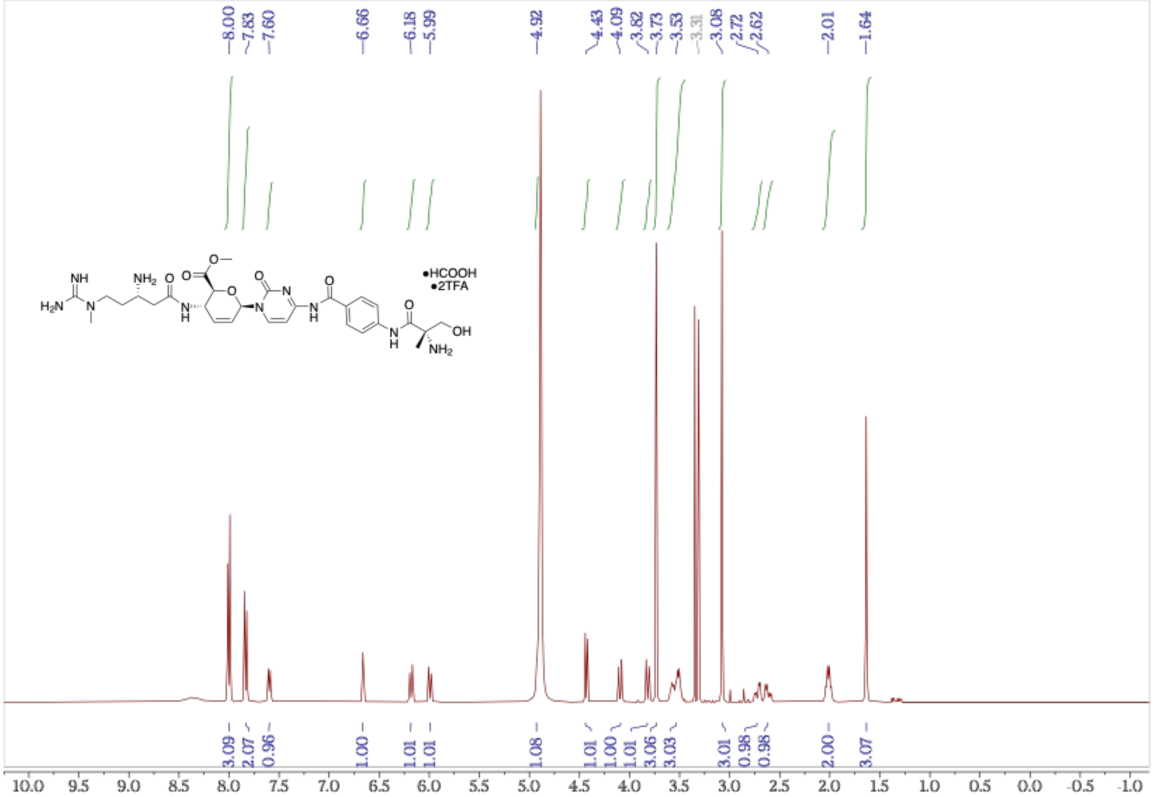

Figure S16. ^{13}C NMR of **22** (125 MHz, CD_3OD).

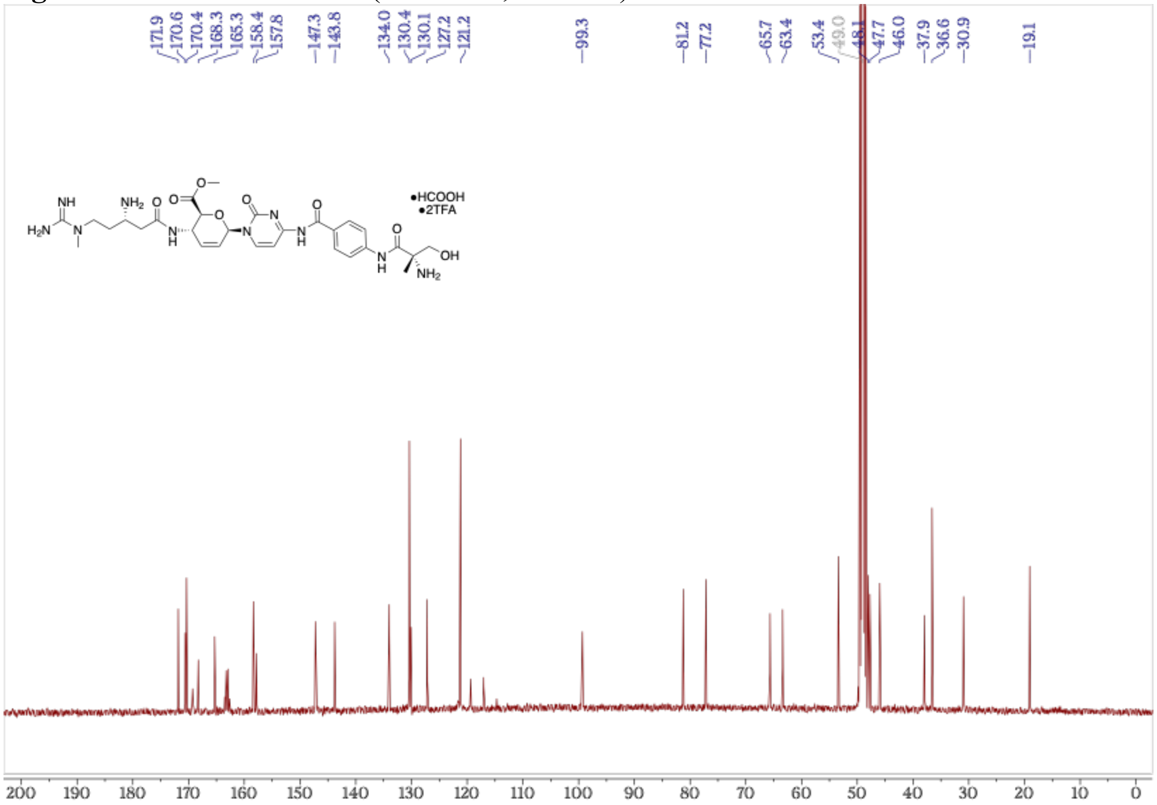

Figure S17. ^{1}H NMR of **25** (400 MHz, CD_3OD).

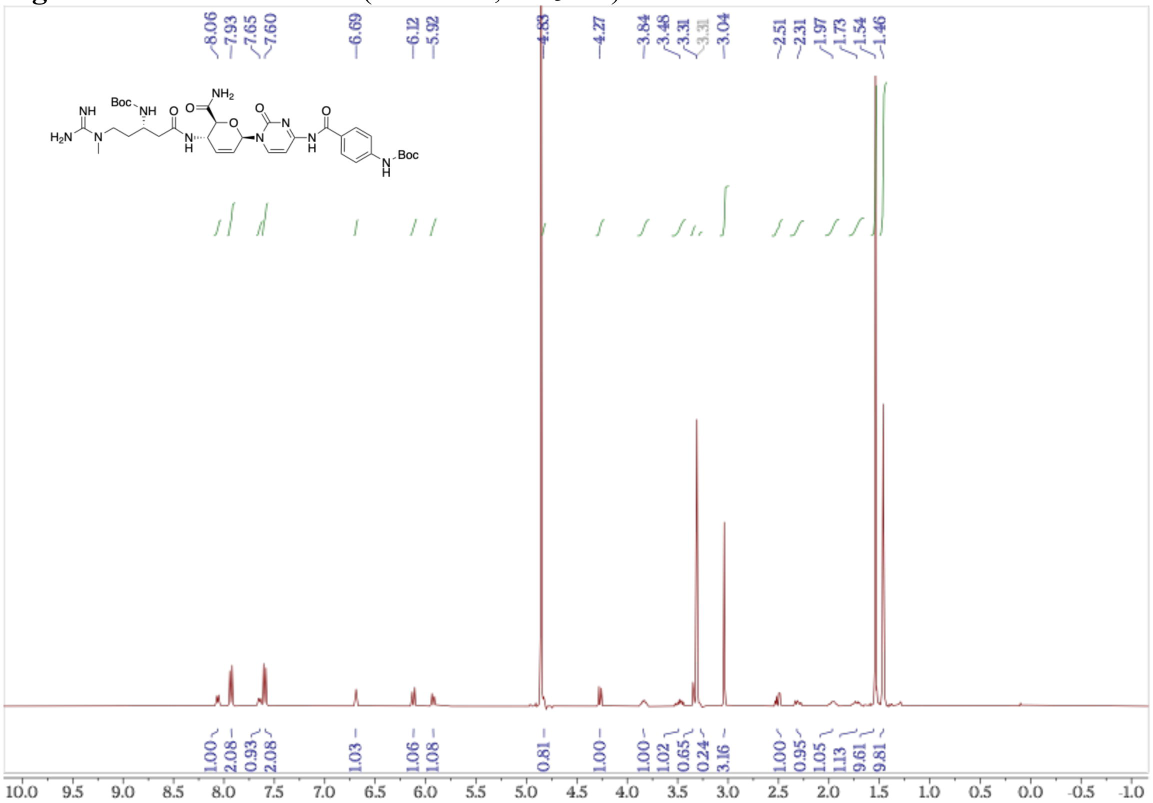

Figure S18. ^{13}C NMR of **25** (125 MHz, CD_3OD).

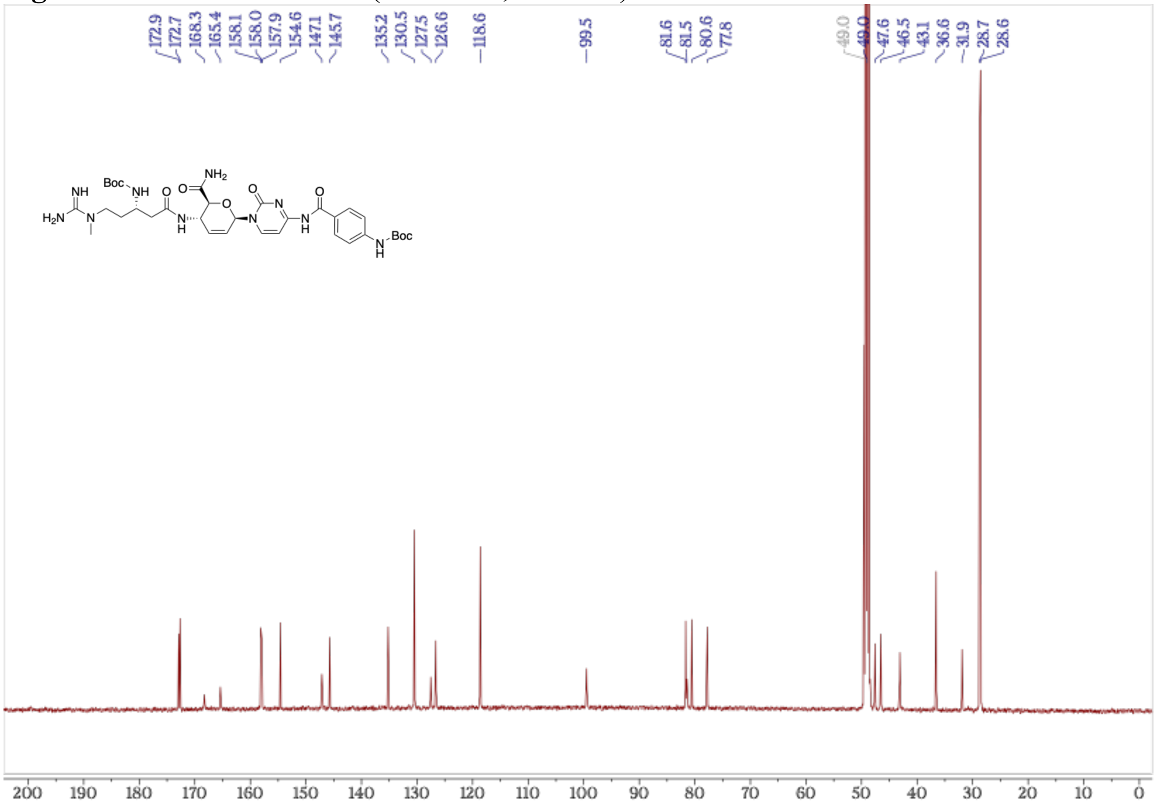

Figure S19. ^{1}H NMR of **26** (500 MHz, CD_3OD).

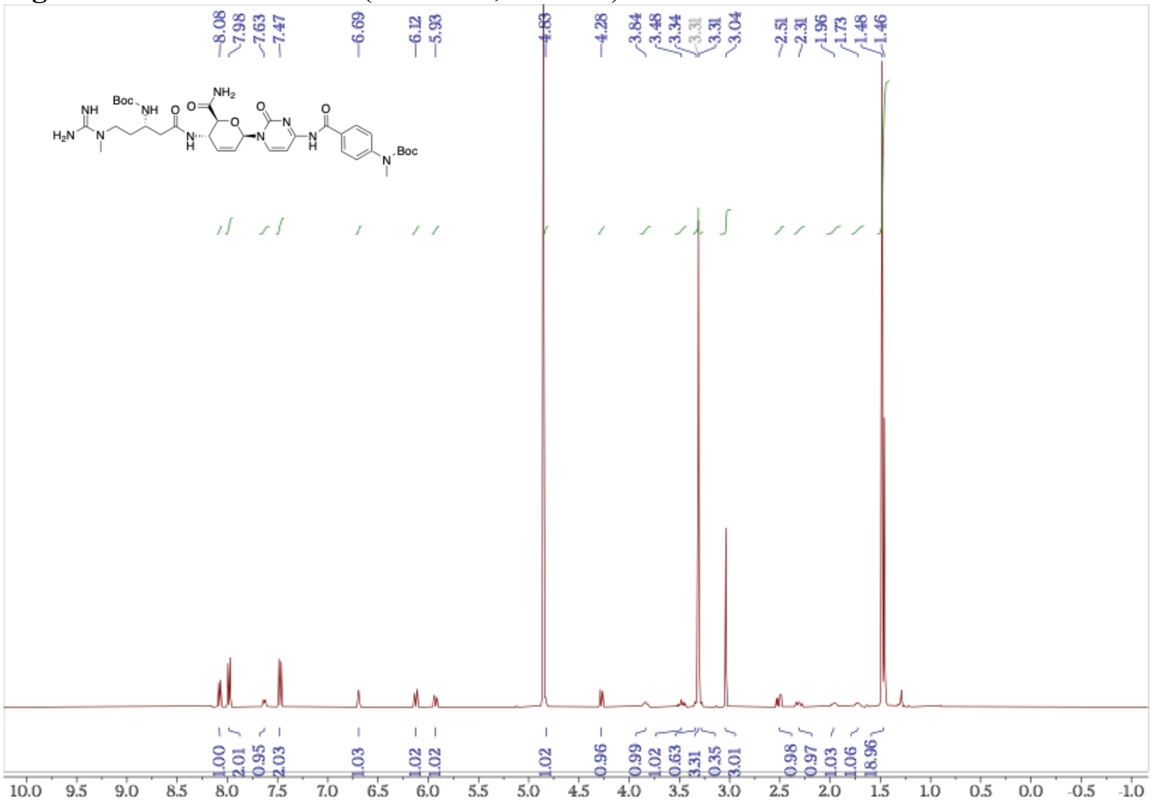

Figure S20. ^{13}C NMR of **26** (125 MHz, CD_3OD).

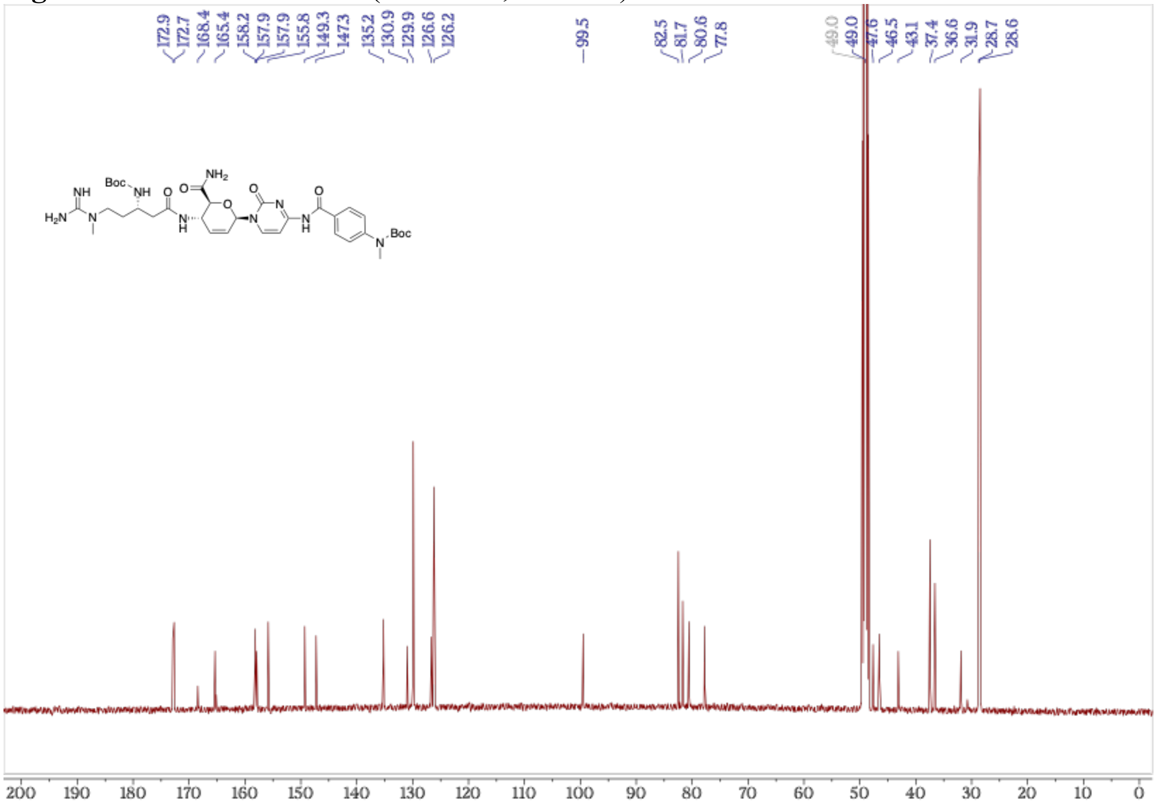

Figure S21. ^{1}H NMR of **27** (400 MHz, CD_3OD).

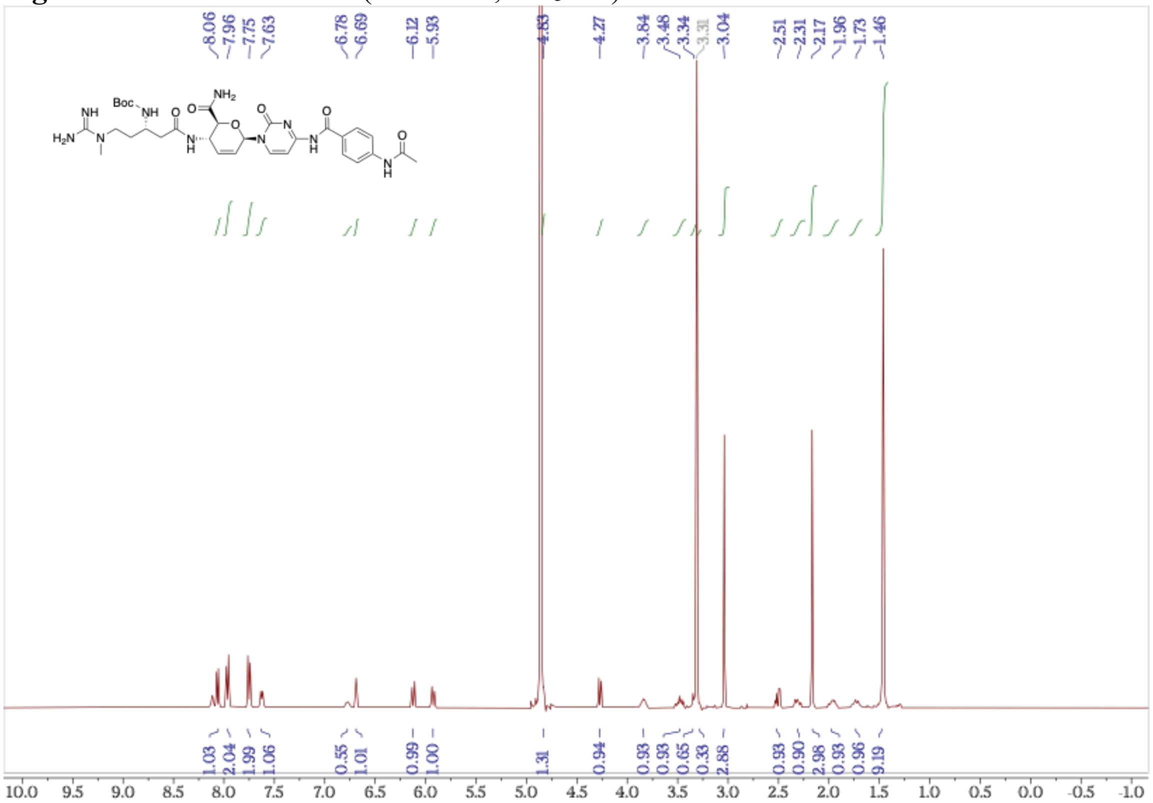

Figure S22. ^{13}C NMR of **27** (125 MHz, CD_3OD).

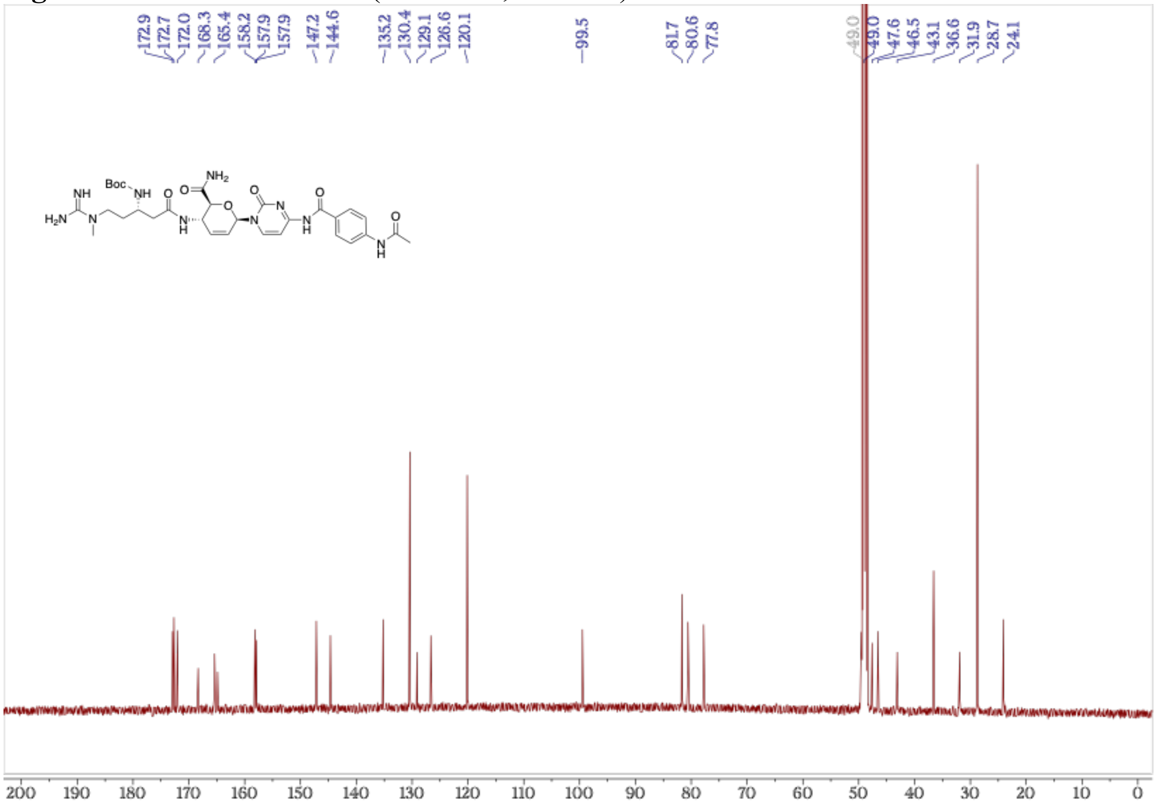

Figure S23. ^{1}H NMR of **28** (500 MHz, CD_3OD).

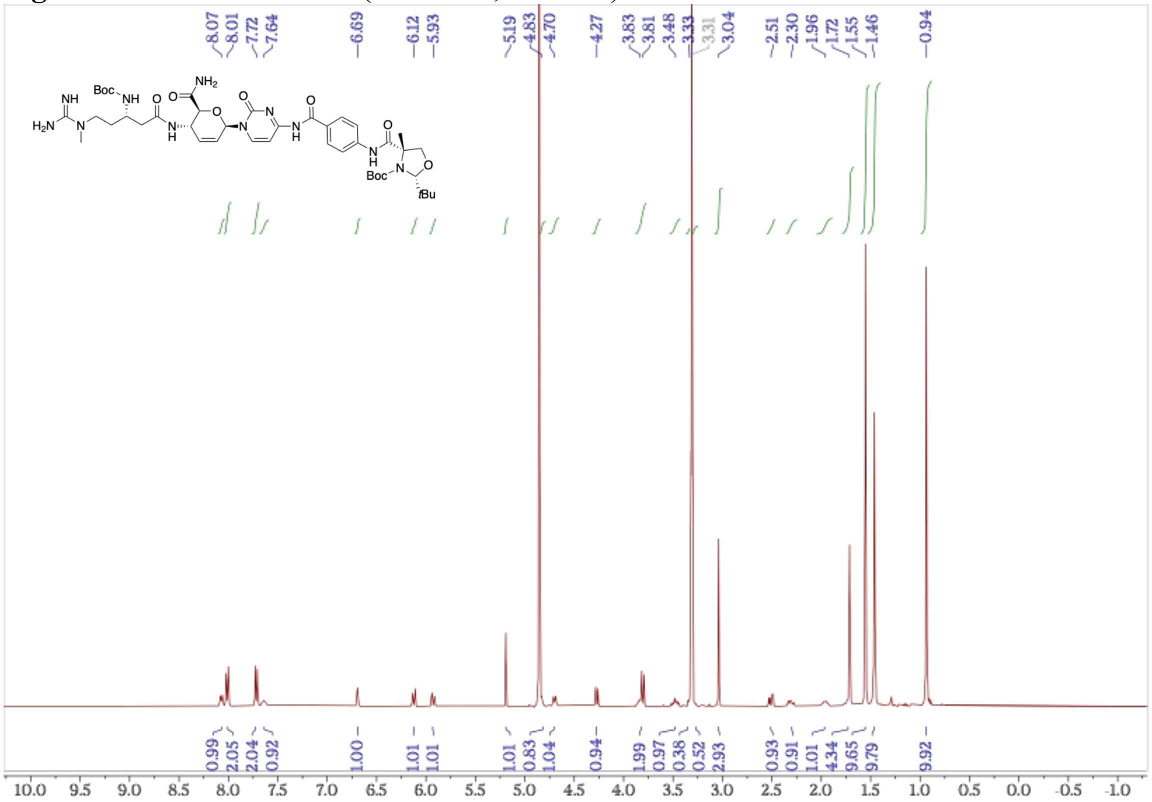

Figure S24. ^{13}C NMR of **28** (125 MHz, CD_3OD).

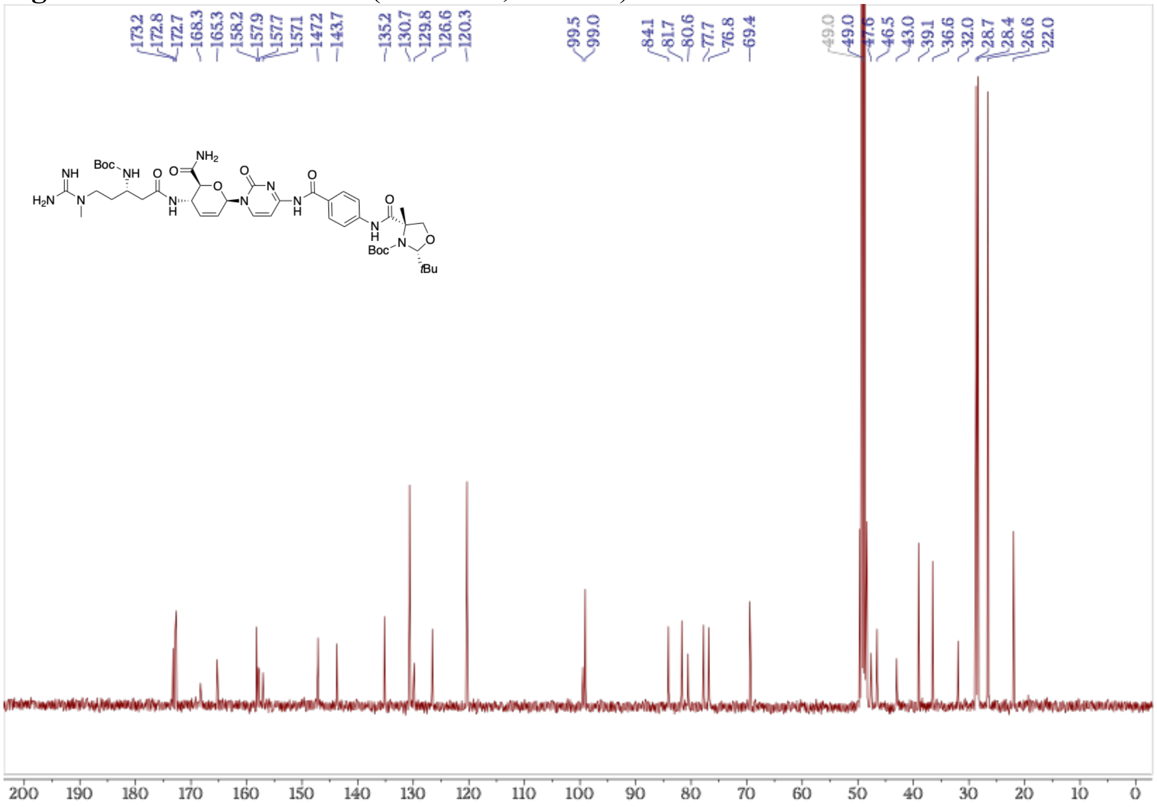

Figure S25. ^{1}H NMR of **29** (500 MHz, CD_3OD).

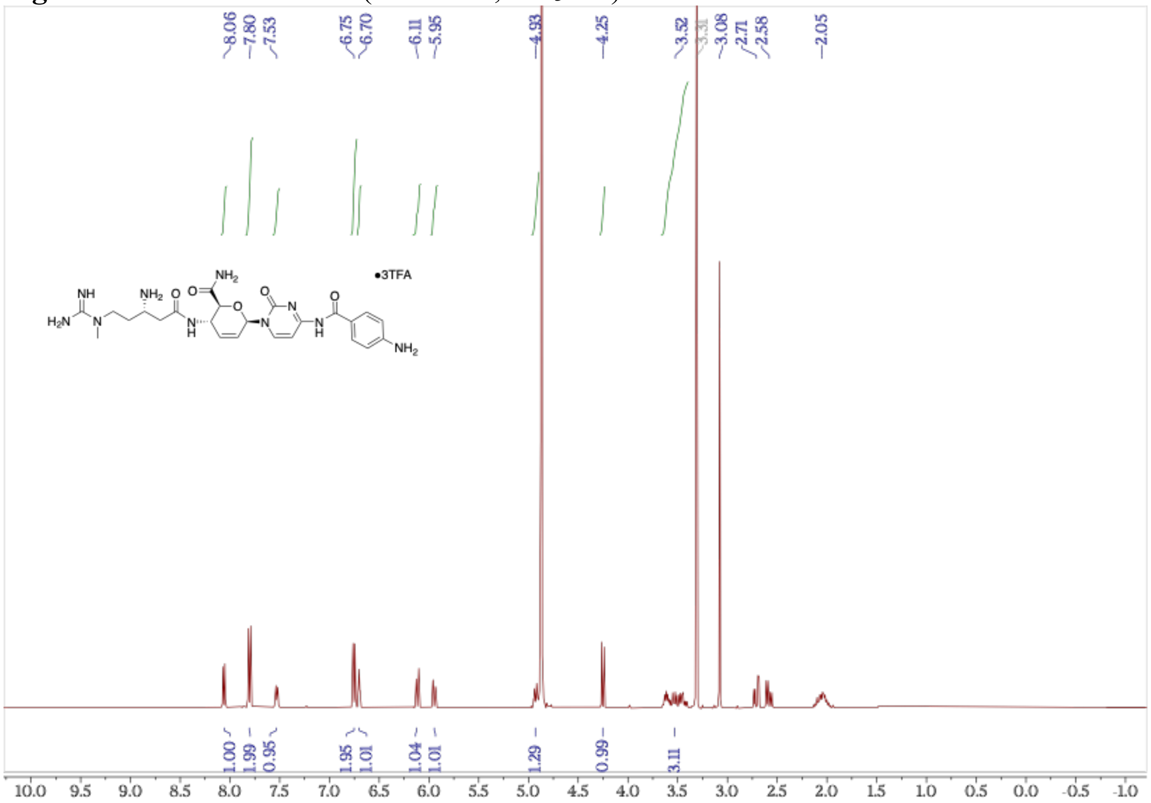

Figure S26. ^{13}C NMR of **29** (125 MHz, CD_3OD).

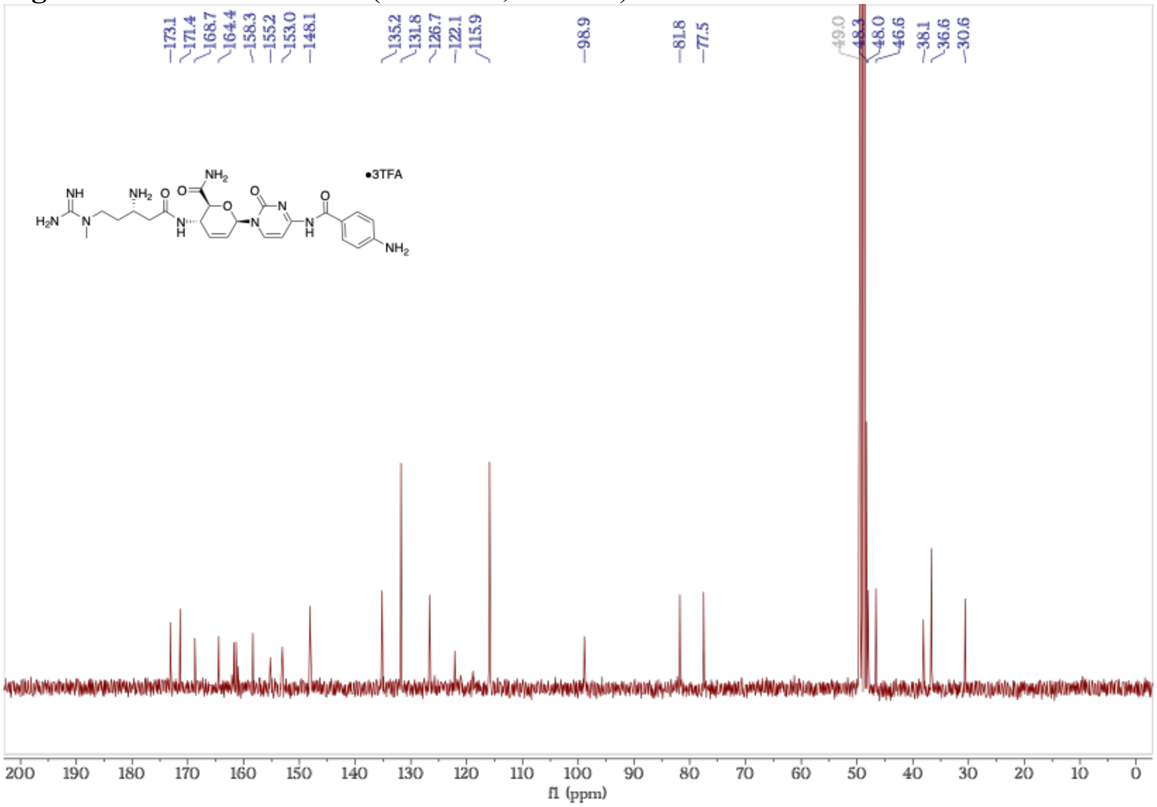

Figure S27. ^{1}H NMR of **30** (400 MHz, CD_3OD).

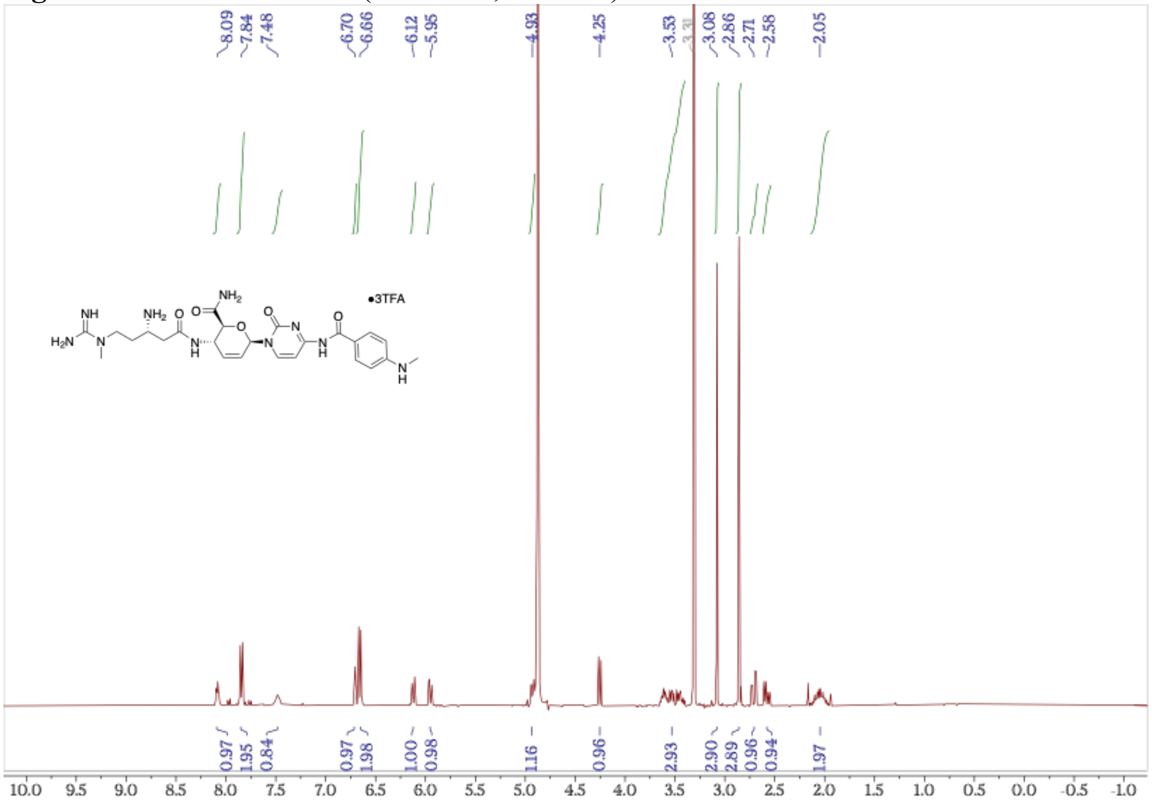

Figure S28. ^{13}C NMR of **30** (125 MHz, CD_3OD).

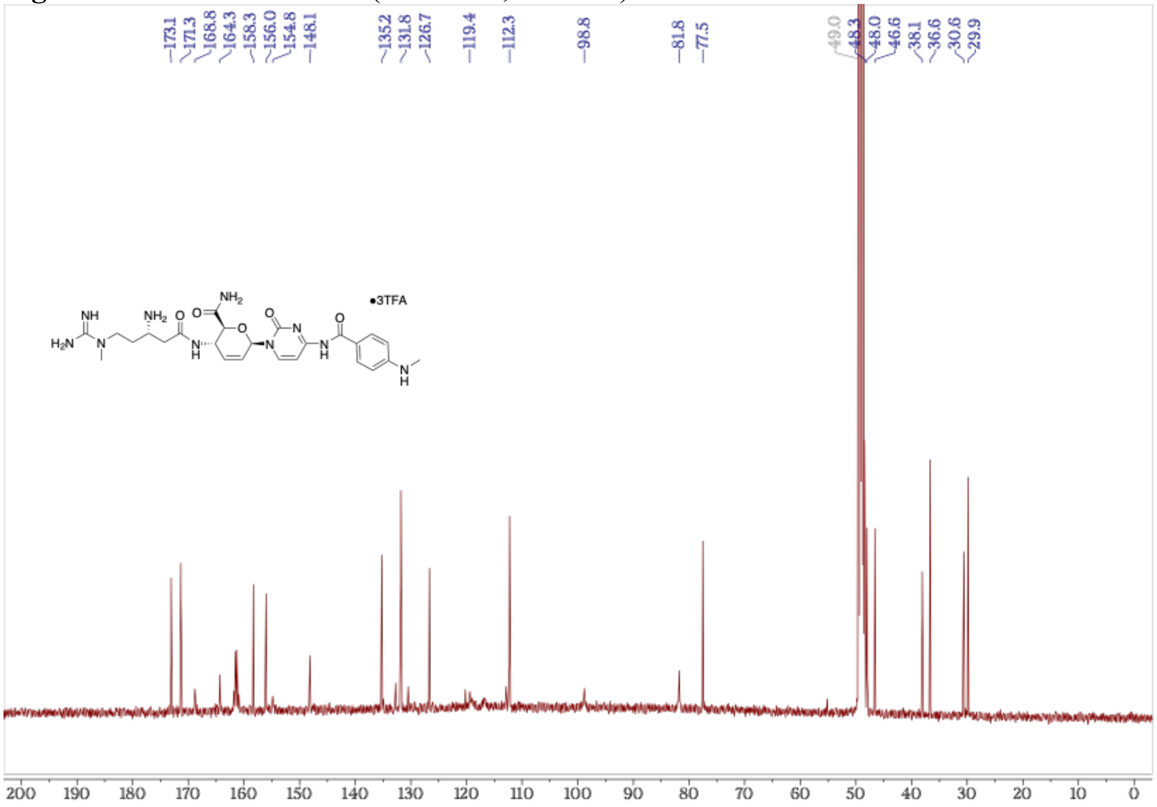

Figure S29. ^{1}H NMR of **31** (400 MHz, CD_3OD).

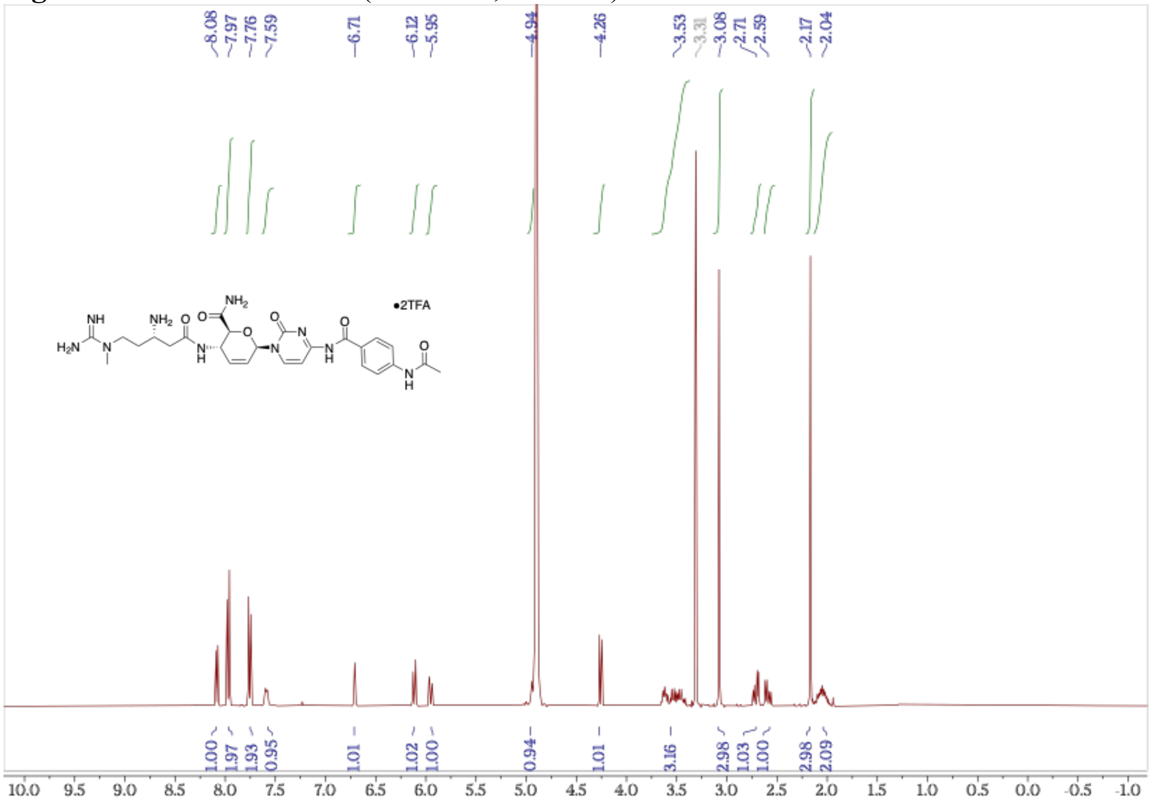

Figure S30. ^{13}C NMR of **31** (125 MHz, CD_3OD).

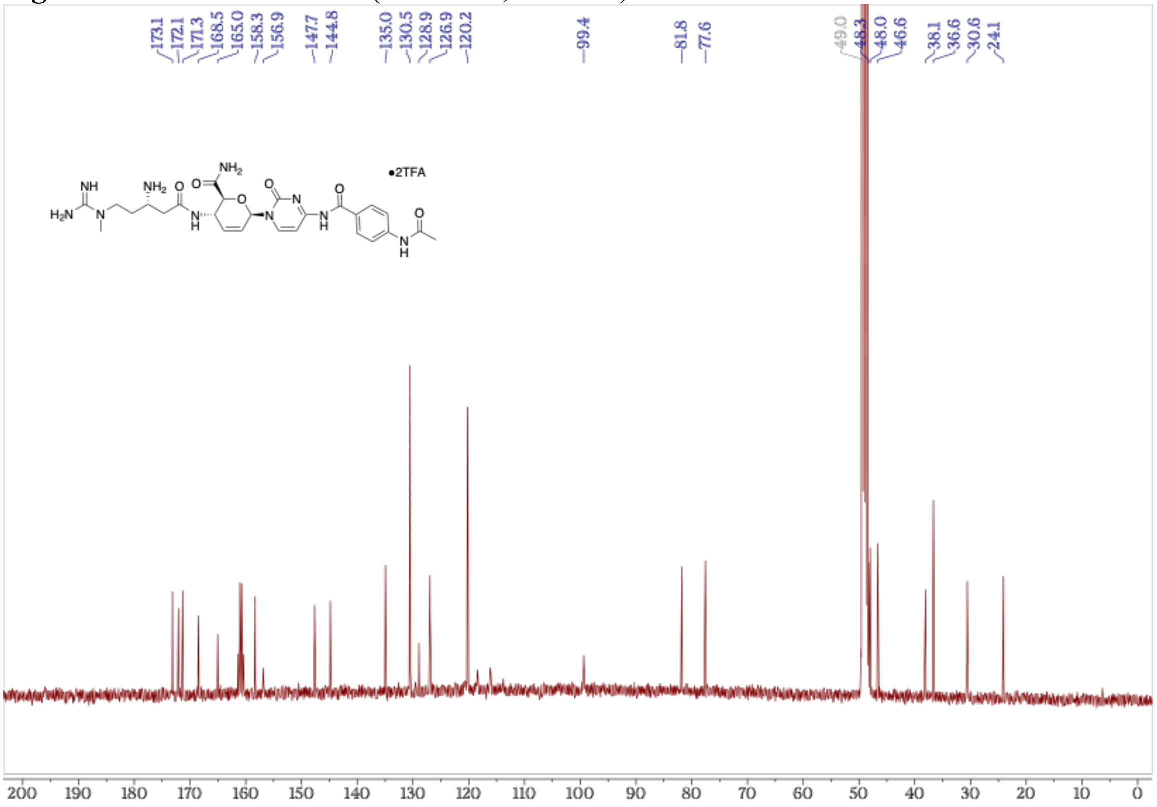

Figure S31. ^{1}H NMR of **32** (400 MHz, CD_3OD).

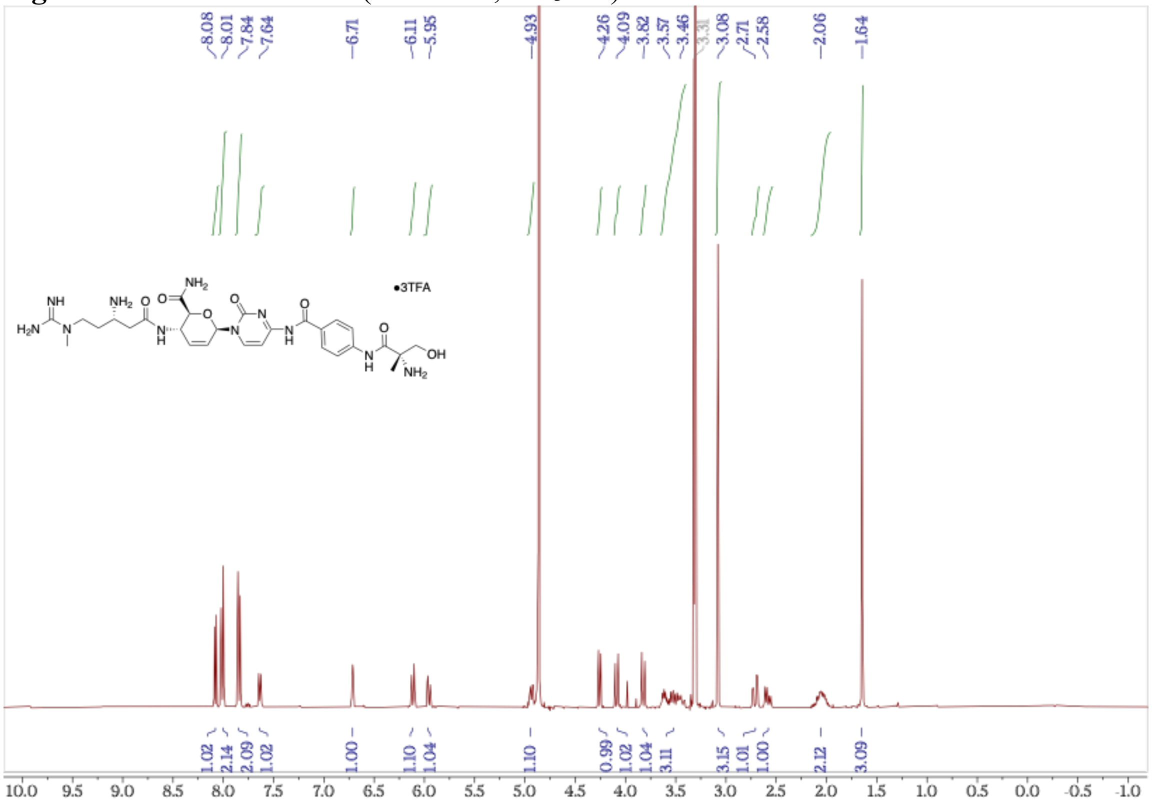

Figure S32. ^{13}C NMR of **32** (125 MHz, CD_3OD).

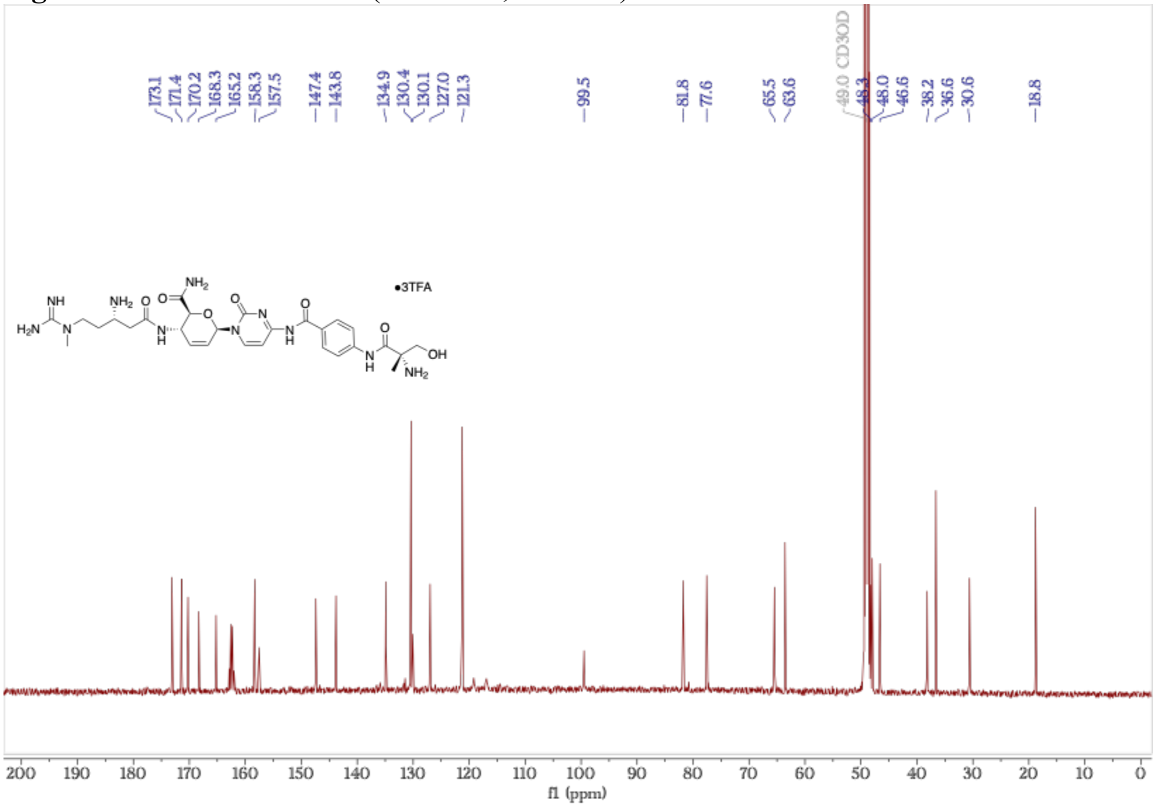

Figure S33. ^{1}H NMR of **36** (400 MHz, CD_3OD).

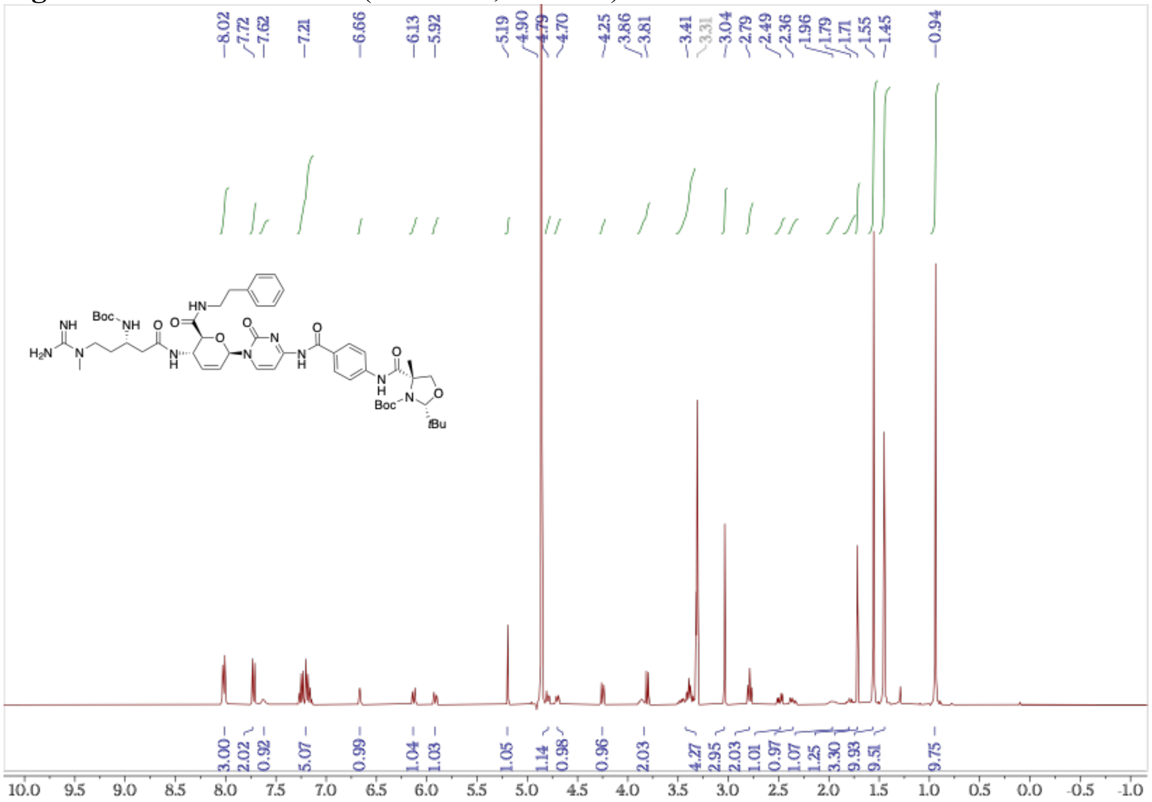

Figure S34. ^{13}C NMR of **36** (125 MHz, CD_3OD).

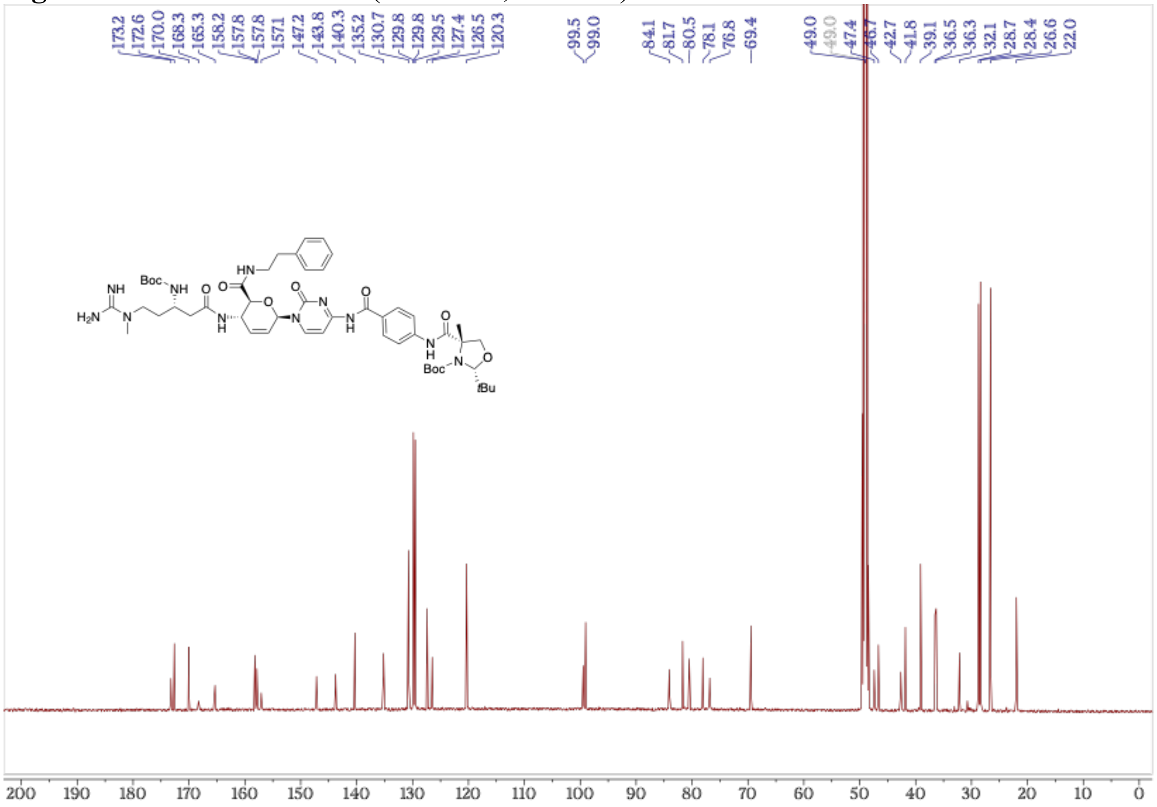

Figure S35. ^{1}H NMR of **37** (400 MHz, CD_3OD) plus trace grease.

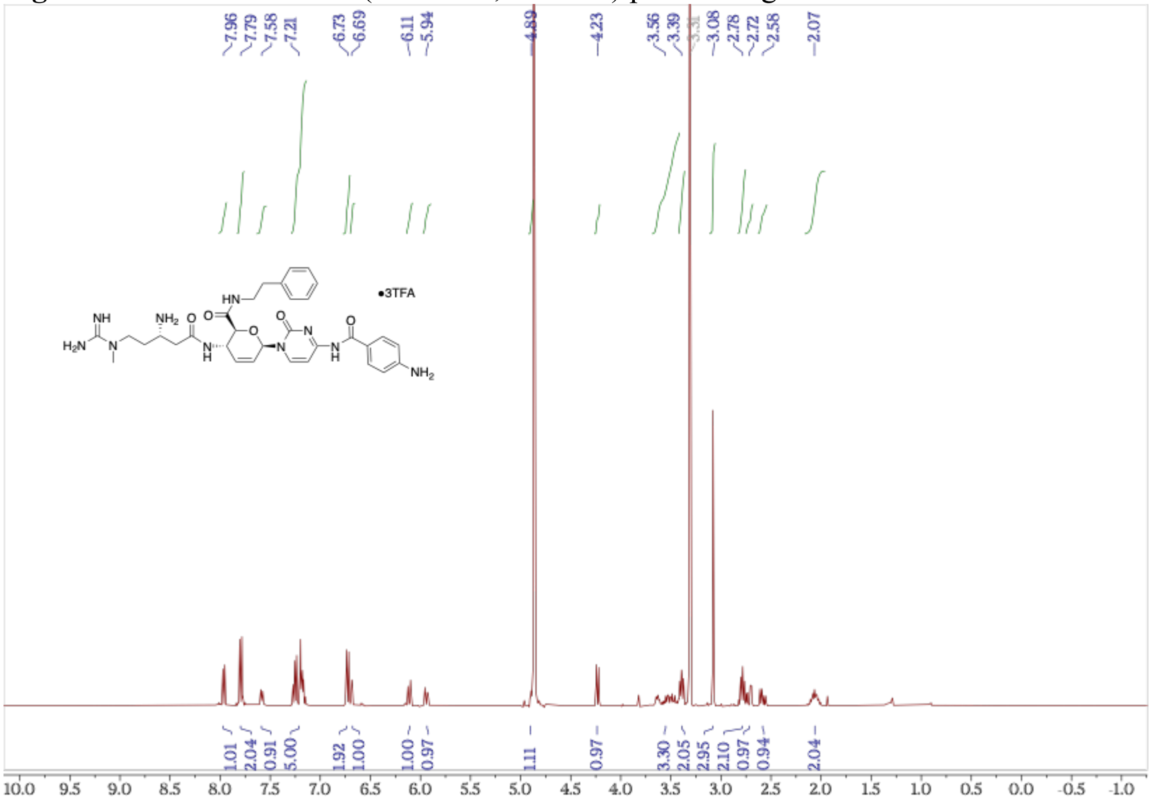

Figure S36. ^{13}C NMR of **37** (125 MHz, CD_3OD).

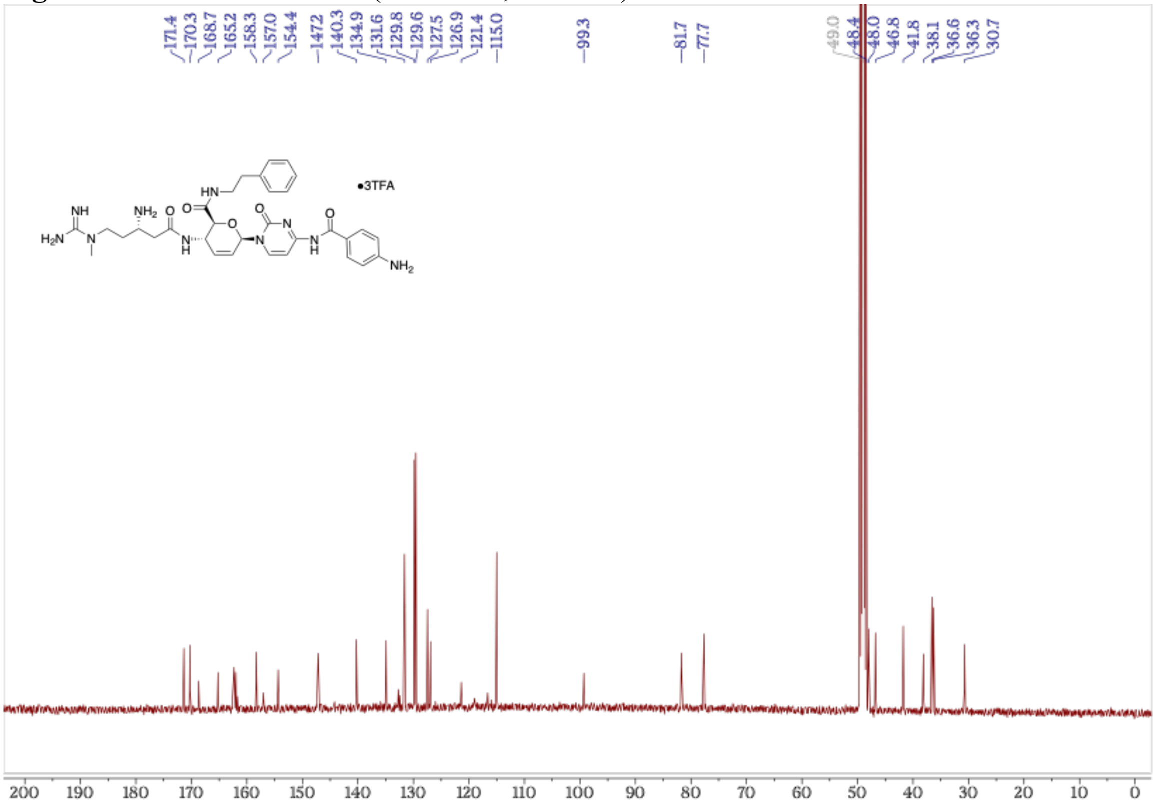

Figure S37. ^{1}H NMR of **38** (400 MHz, CD_3OD).

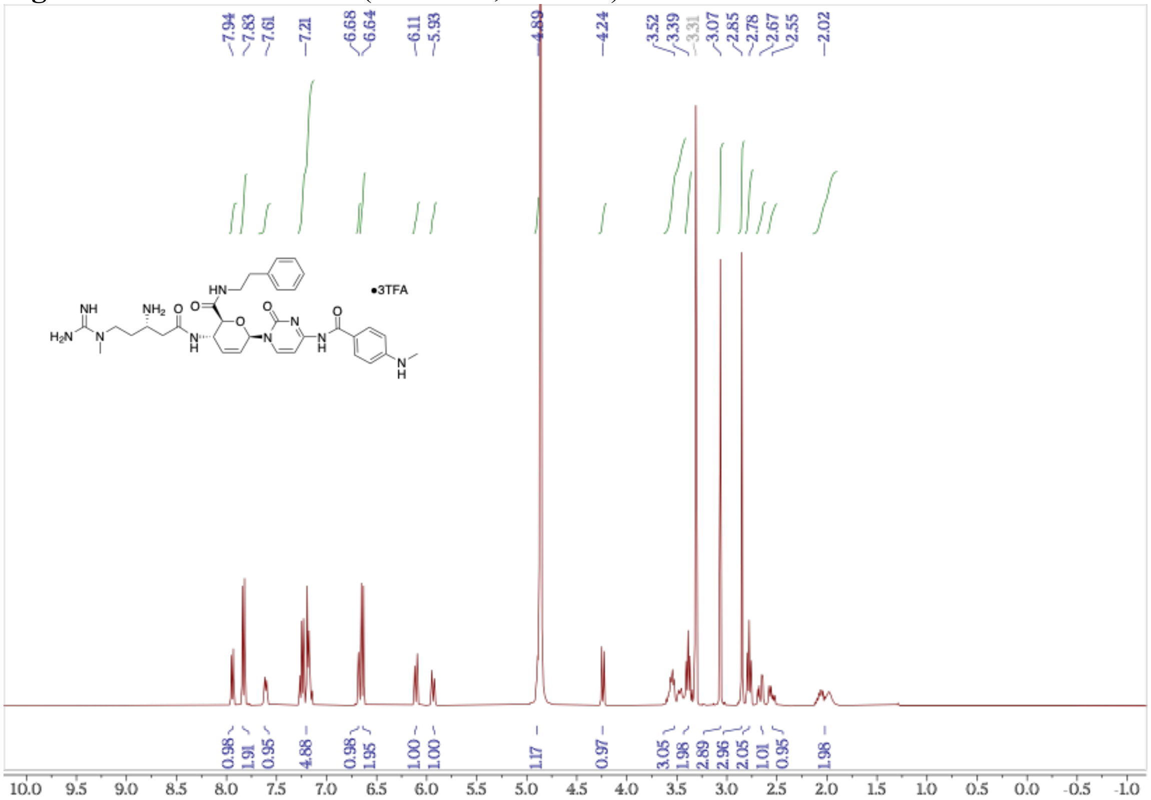

Figure S38. ^{13}C NMR of **38** (125 MHz, CD_3OD).

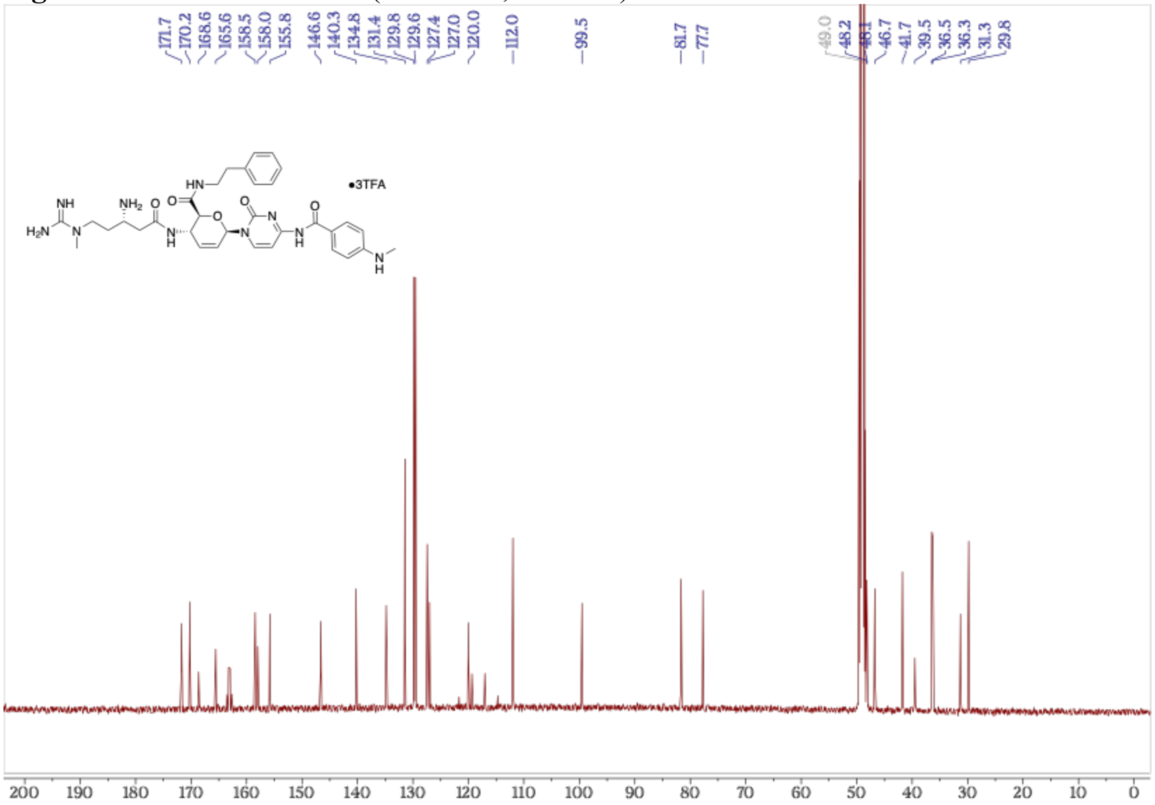

Figure S39. ^{1}H NMR of **39** (400 MHz, CD_3OD).

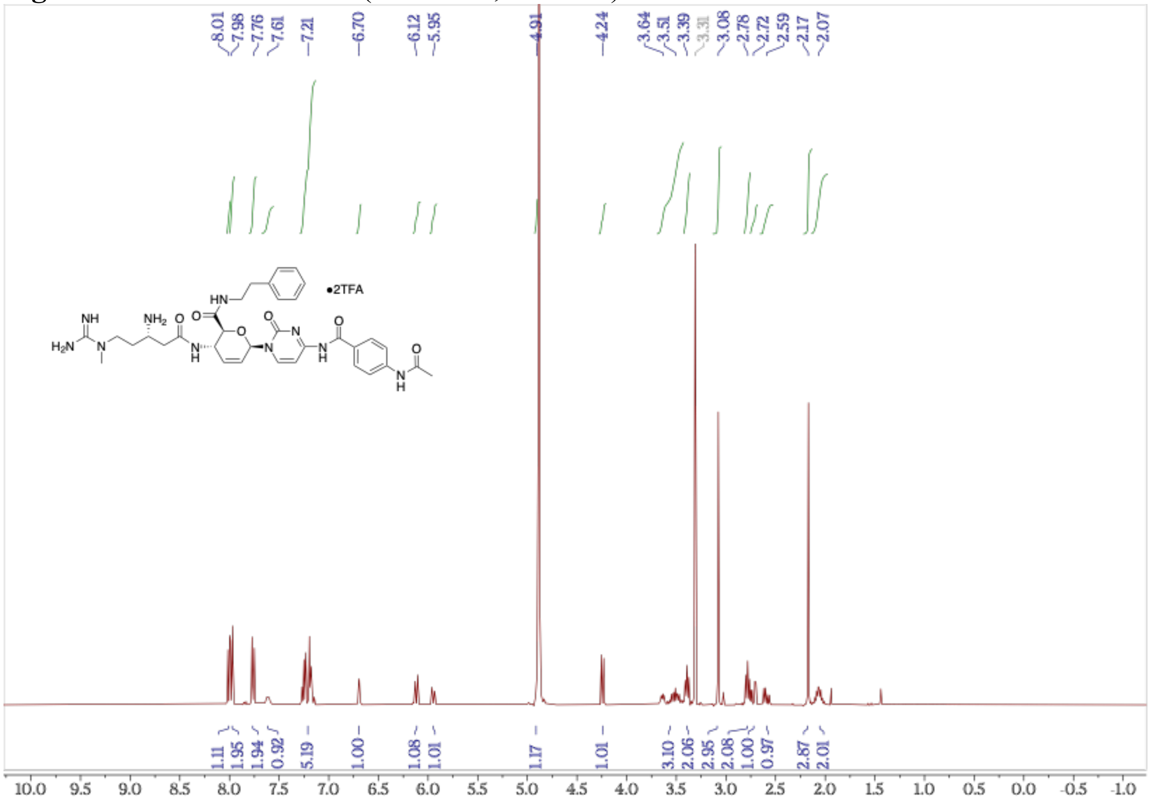

Figure S40. ^{13}C NMR of **39** (125 MHz, CD_3OD).

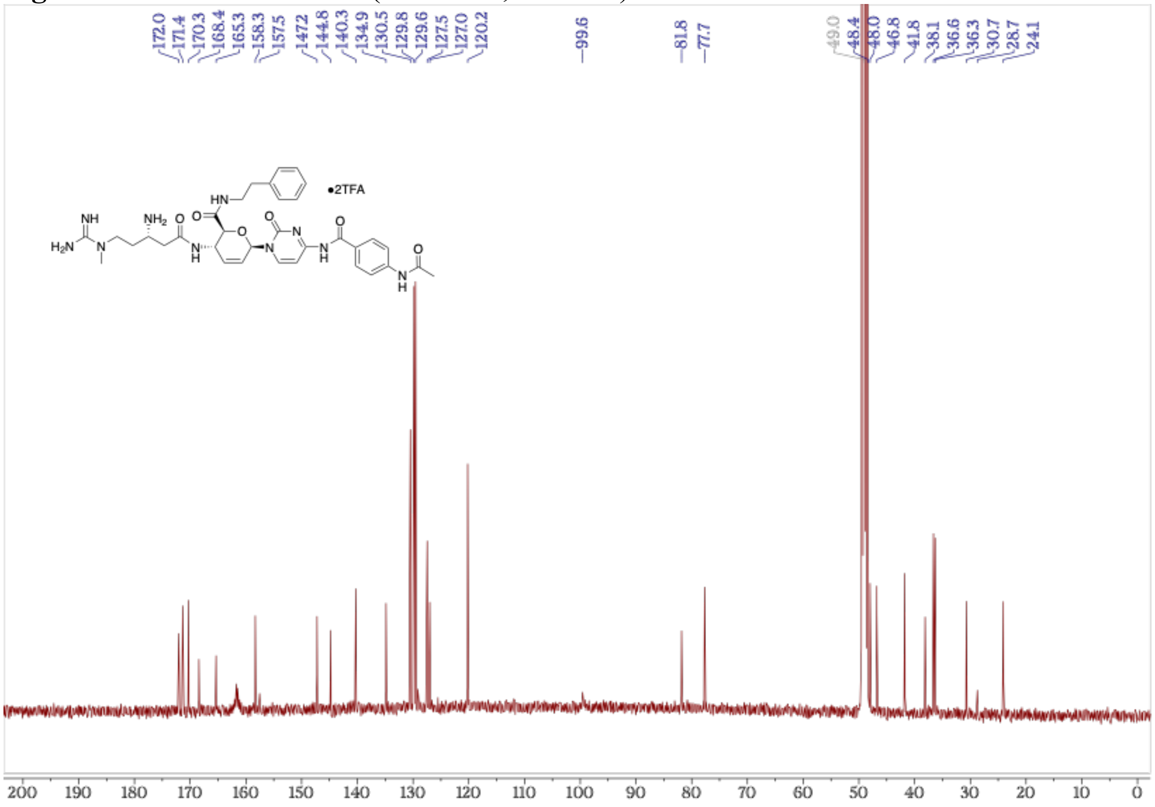

Figure S41. ^{1}H NMR of **40** (400 MHz, CD_3OD) plus trace methyl trifluoroacetate.

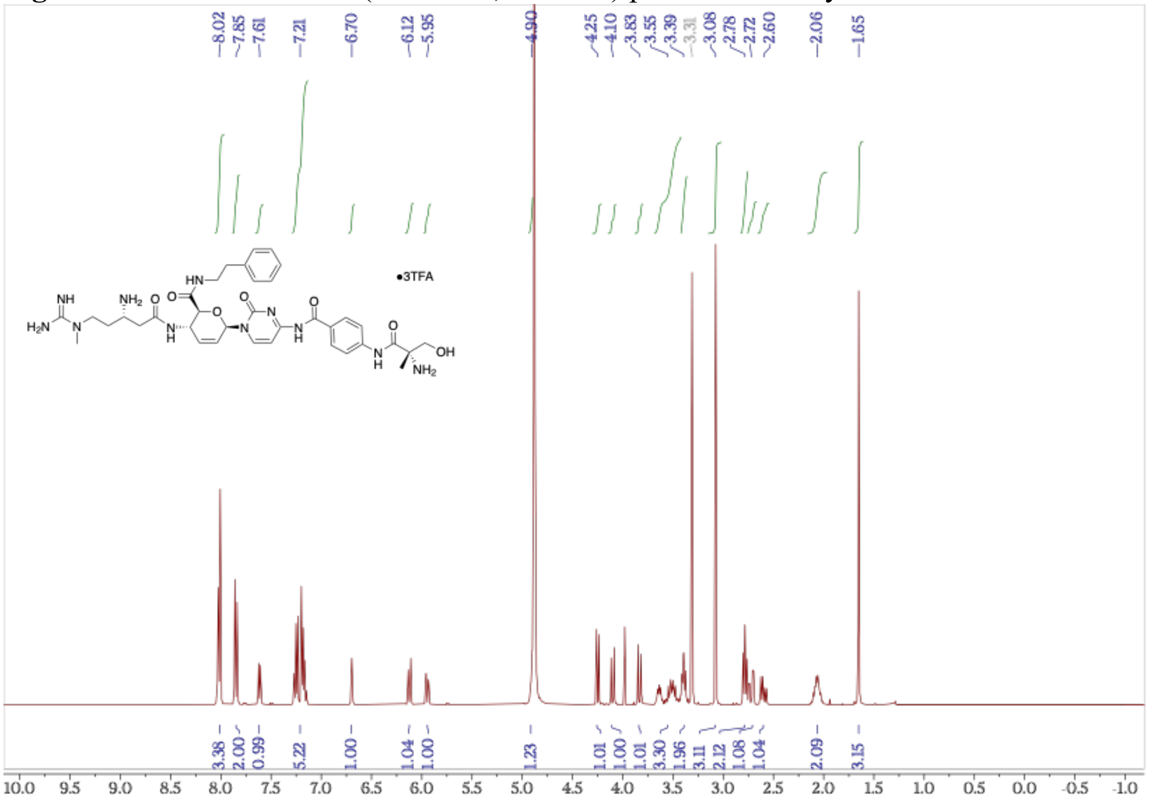

Figure S42. ^{13}C NMR of **40** (125 MHz, CD_3OD) plus trace methyl trifluoroacetate.

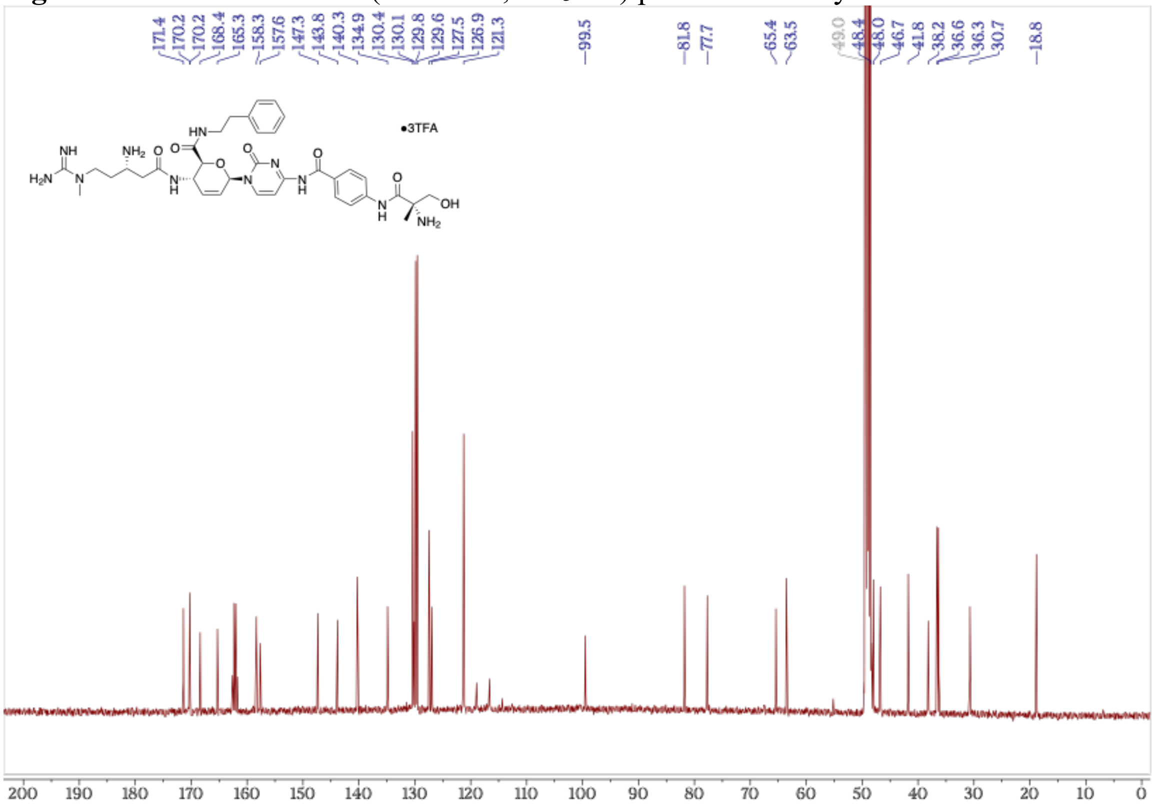

Figure S43. ^{1}H NMR of **43** (600 MHz, CD_3OD) plus trace Et_3N HCl.

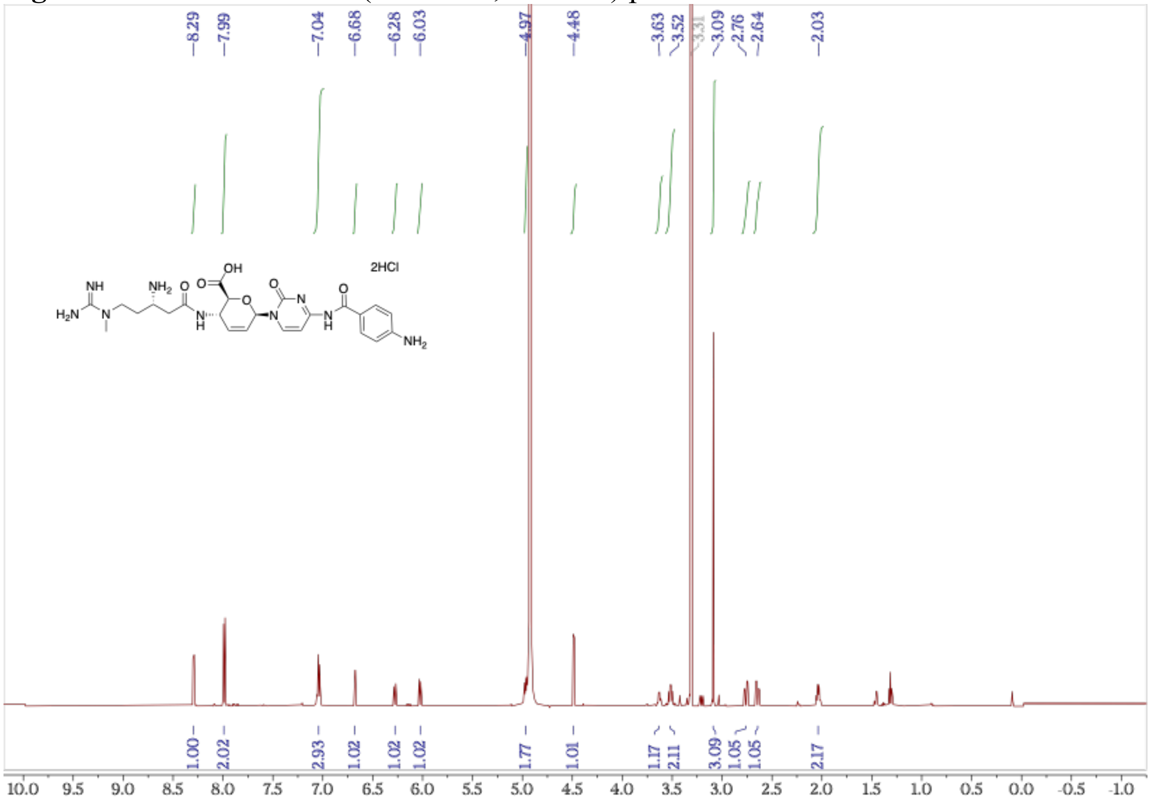

Figure S44. ^{13}C NMR of **43** (125 MHz, CD_3OD) plus trace Et_3N HCl.

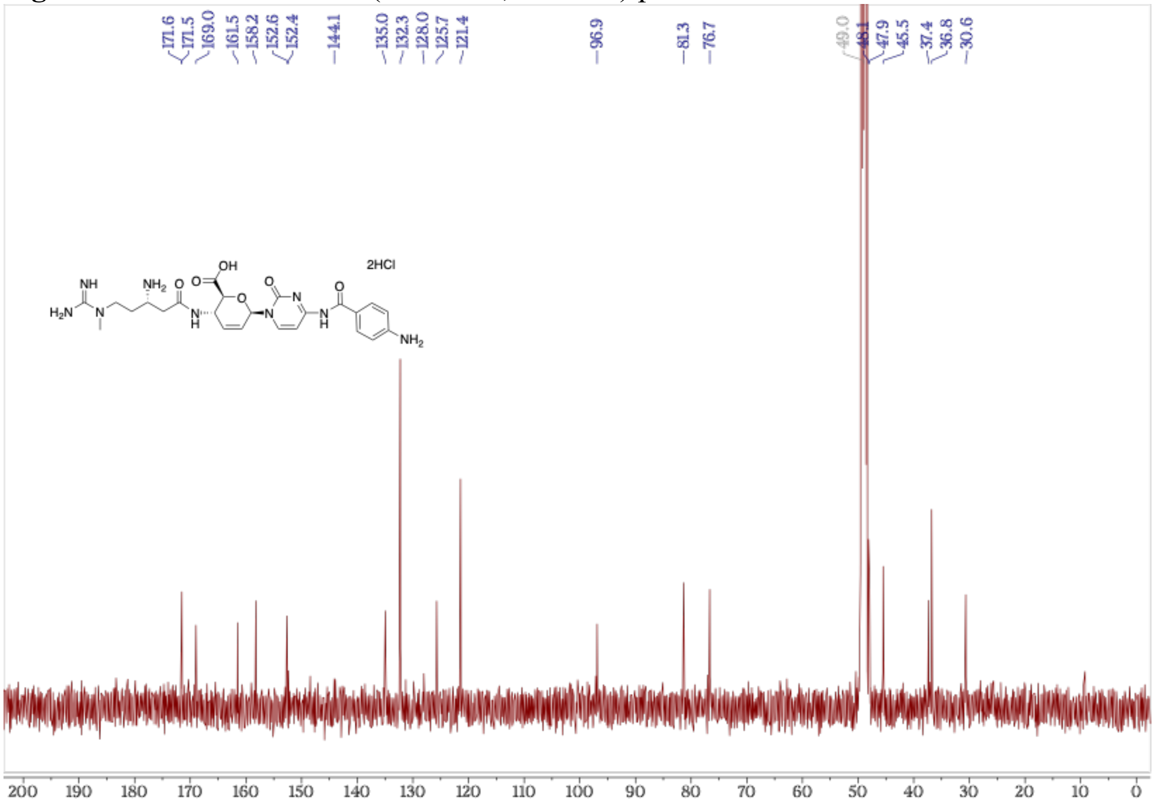

Figure S45. ^{1}H NMR of **44** (600 MHz, D_2O) plus trace formic acid and *N*-methyl PABA.

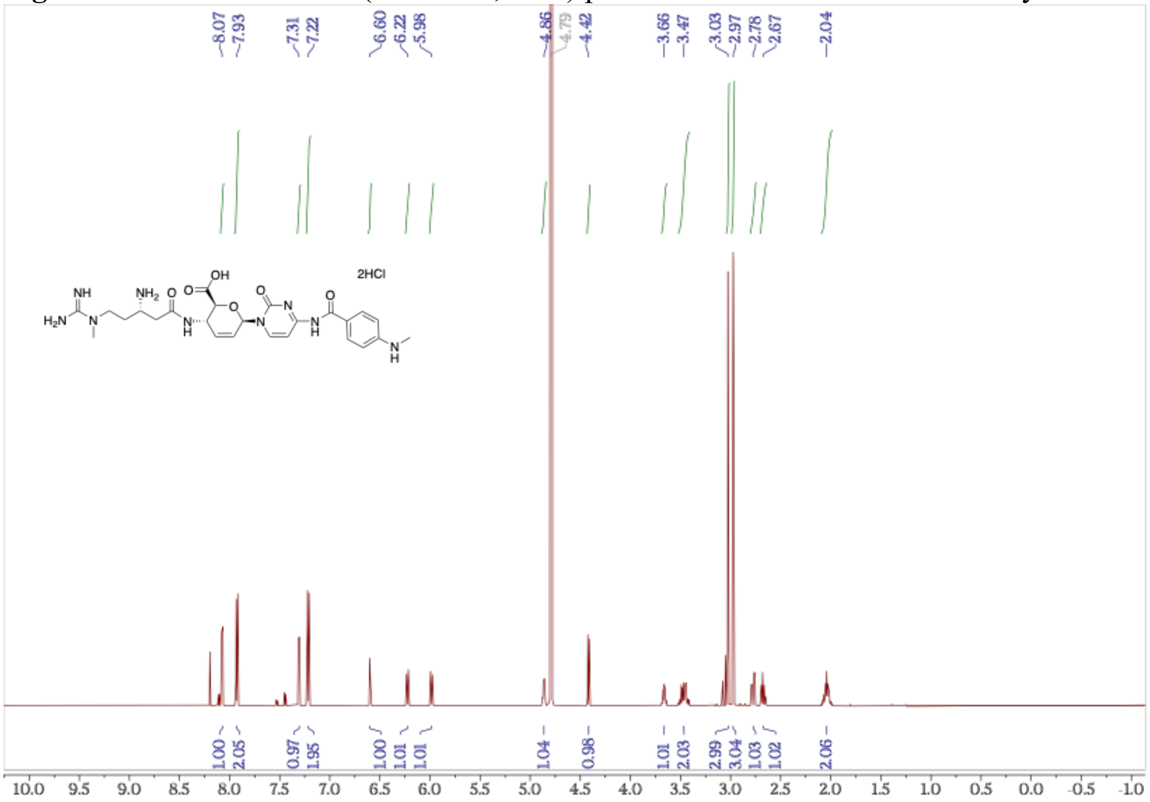

Figure S46. ^{13}C NMR of **44** (125 MHz, D_2O) plus trace formic acid and *N*-methyl PABA.

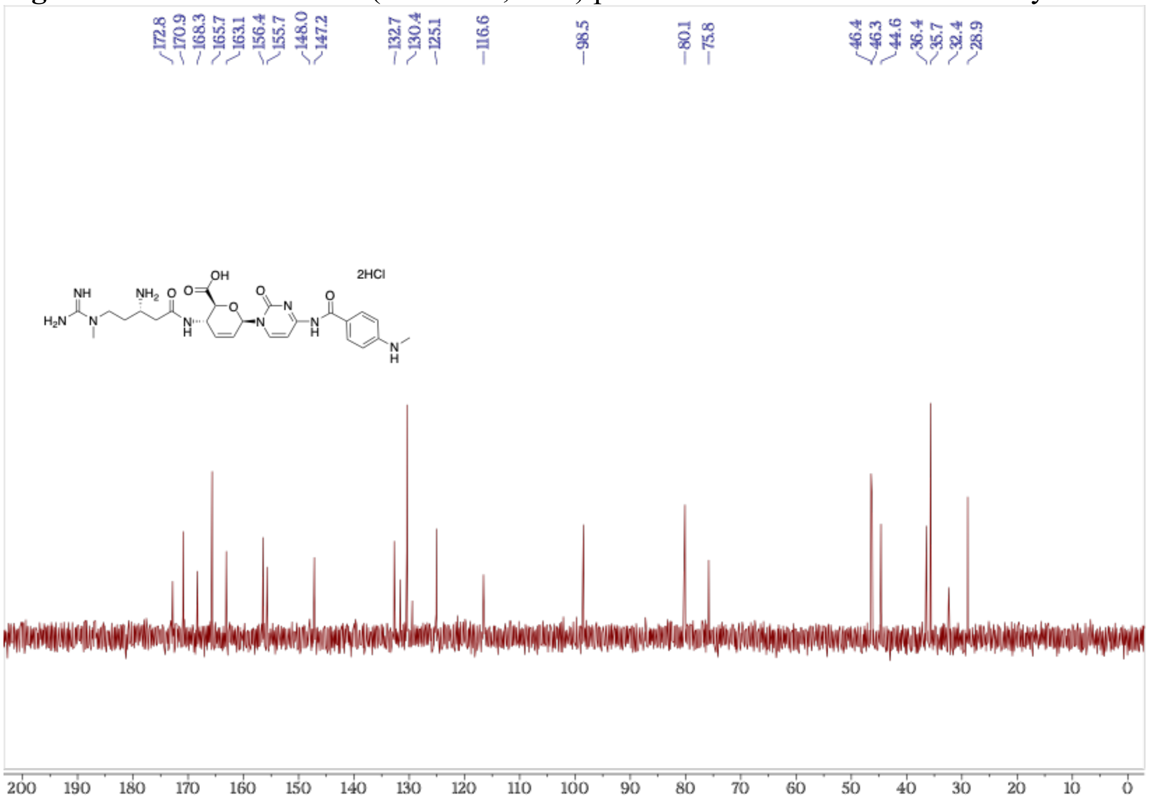

Figure S47. ^{1}H NMR of **45** (600 MHz, D_2O) plus trace formic acid and *N*-acetyl PABA.

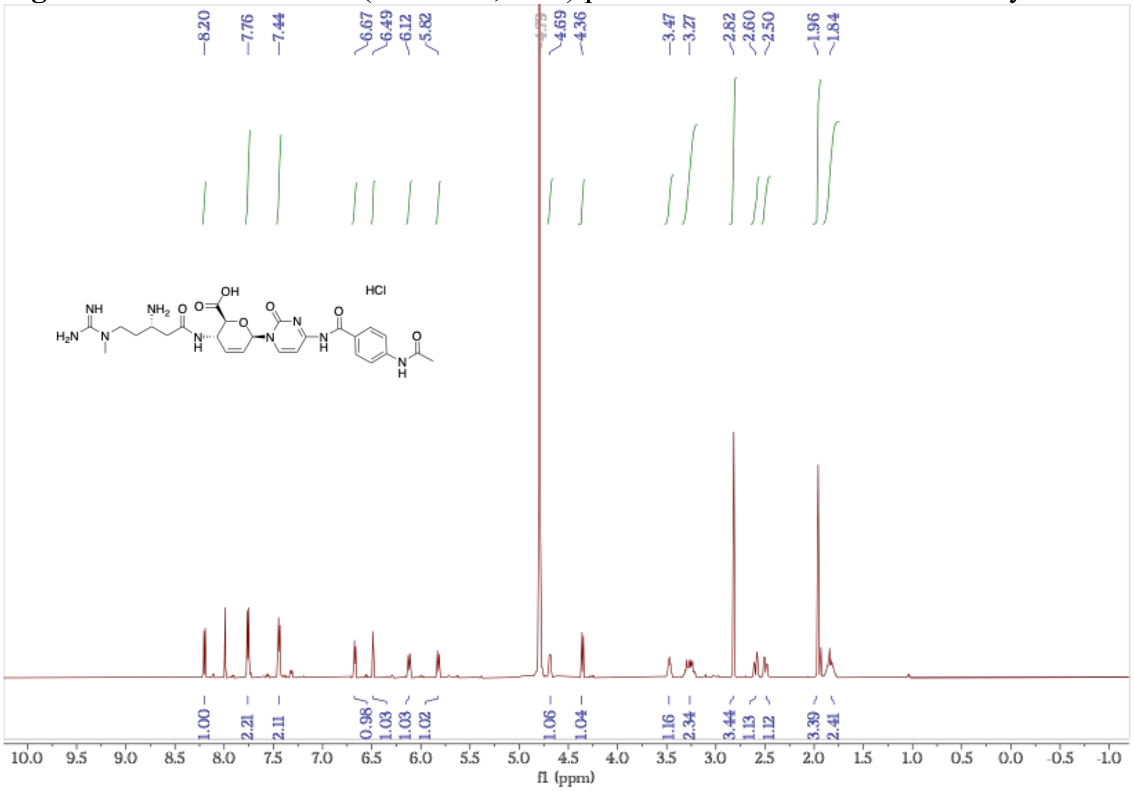

Figure S48. ^{13}C NMR of **45** (125 MHz, D_2O) plus trace formic acid and *N*-acetyl PABA.

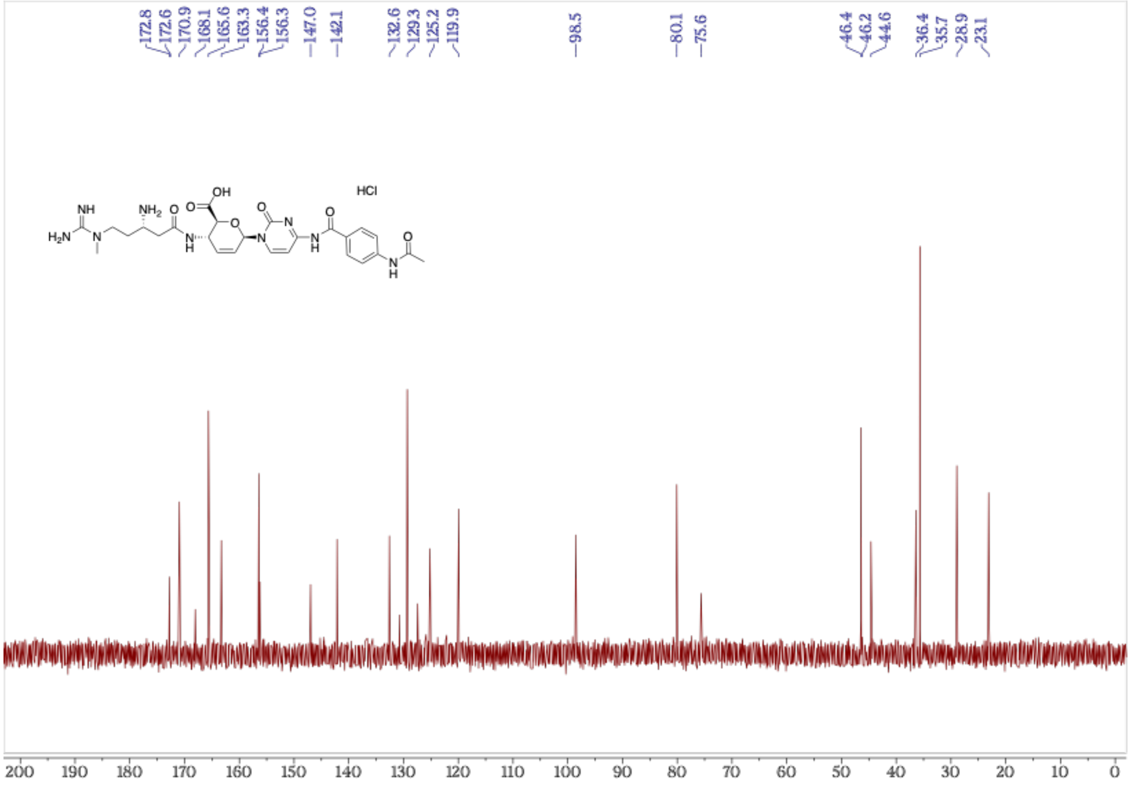

Figure S49. ^{1}H NMR of **46** (600 MHz, D_2O) plus trace Et_3N HCl.

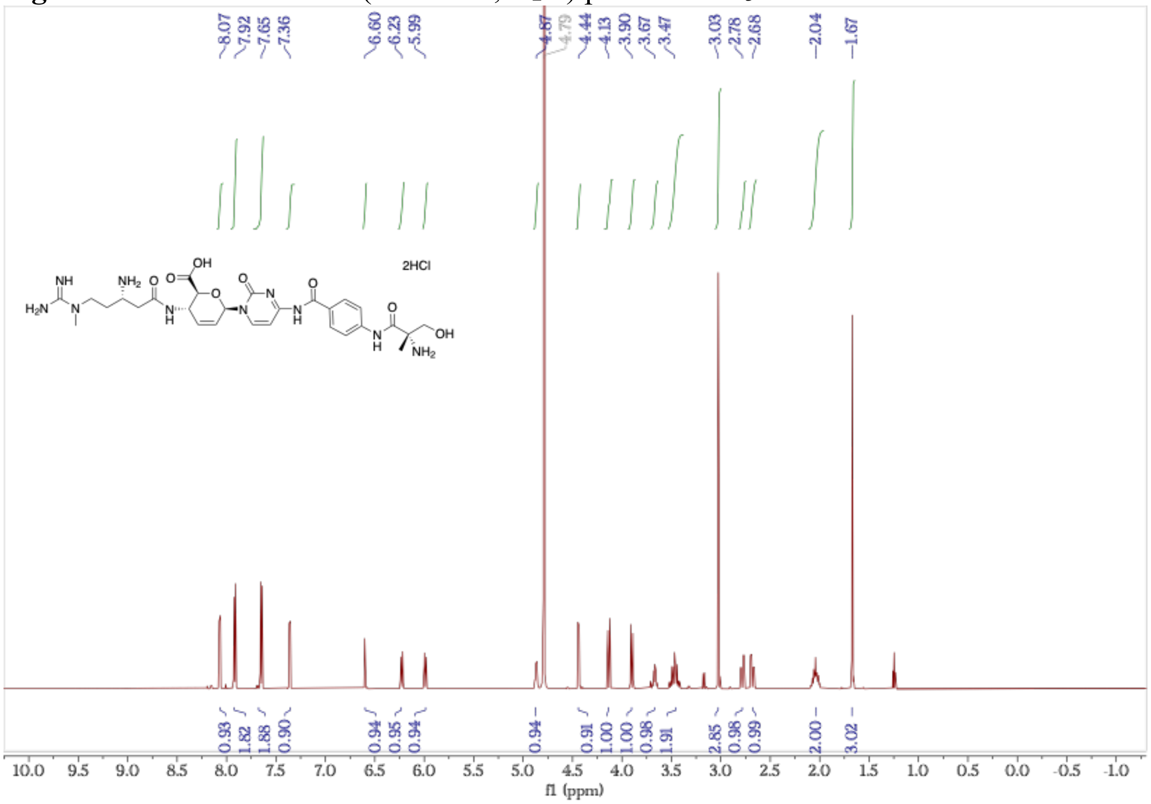

Figure S50. ^{13}C NMR of **46** (125 MHz, D_2O) plus trace Et_3N HCl.

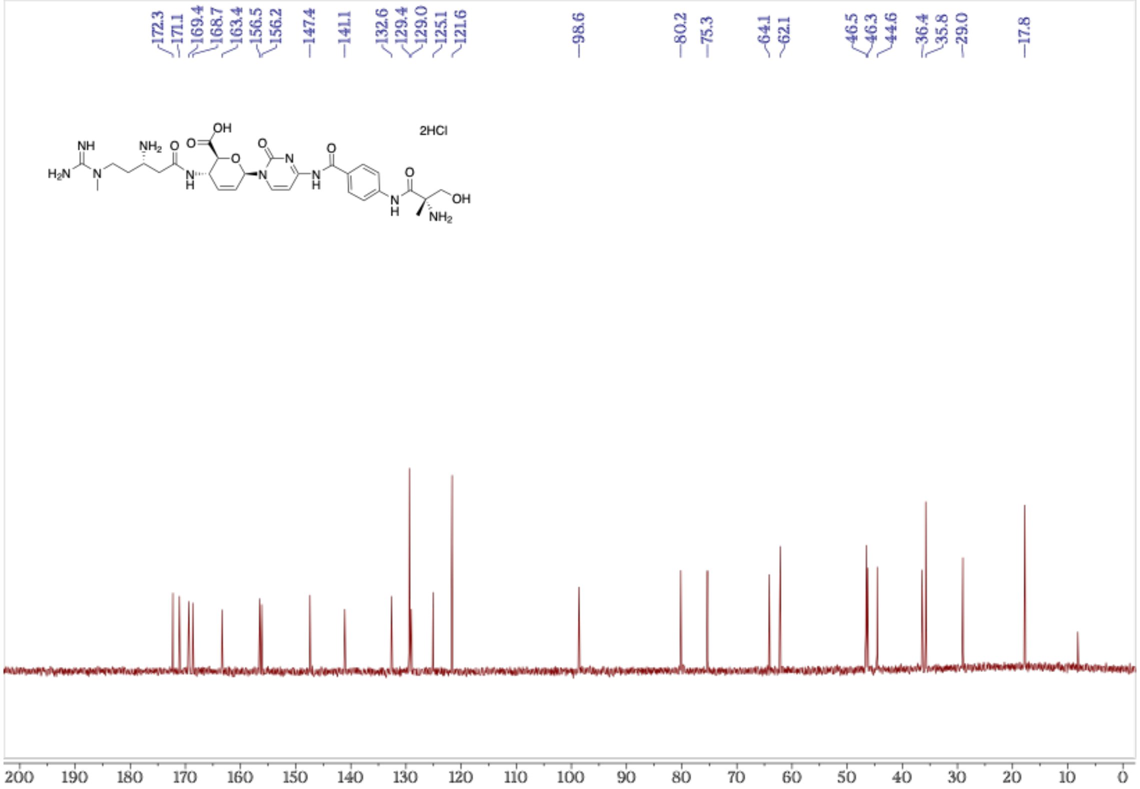

References

(1) Gannett, C.; Tiller, K.; Briganti, A. J.; Brown, A. M.; Weger-Lucarelli, J.; Lowell, A. N. Forgotten natural products: Semisynthetic development of blasticidin S as an antibiotic lead. *ACS Med. Chem. Lett.* **2024**, *15* (3), 362-368.

(2) Fu, J.; Xu, P.; Yu, B. Total synthesis of nucleoside antibiotics amicetin, plicacetin, and cytosaminomycin A—D. *Chin. J. Chem .* **2021**, *39* (10), 2679-2684.

(3) Aysun, A.; Yağmur, K.; Yusuf, B. Cell Proliferation and Cytotoxicity Assays. *Curr. Pharm. Biotechnol.* **2016**, *17* (14), 1213-1221.

(4) Mohammad, H.; Abutaleb, N. S.; Seleem, M. N. Auranofin Rapidly Eradicates Methicillin-resistant Staphylococcus aureus (MRSA) in an Infected Pressure Ulcer Mouse Model. *Sci. Rep.* **2020**, *10* (1), 7251.

(5) Mohammad, H.; Kyei-Baffour, K.; Abutaleb, N. S.; Dai, M.; Seleem, M. N. An aryl isonitrile compound with an improved physicochemical profile that is effective in two mouse models of multidrug-resistant Staphylococcus aureus infection. *Journal of Global Antimicrobial Resistance* **2019**, *19*, 1-7.

(6) Hagras, M.; Hegazy, Y. A.; Elkabbany, A. H.; Mohammad, H.; Ghiaty, A.; Abdelghany, T. M.; Seleem, M. N.; Mayhoub, A. S. Biphenylthiazole antibiotics with an oxadiazole linker: An approach to improve physicochemical properties and oral bioavailability. *Eur. J. Med. Chem.* **2018**, *143*, 1448-1456.

(7) Zgadzay, Y.; Kolosova, O.; Stetsenko, A.; Wu, C.; Bruchlen, D.; Usachev, K.; Validov, S.; Jenner, L.; Rogachev, A.; Yusupova, G.; Sachs, M. S.; Guskov, A.; Yusupov, M. E-site drug specificity of the human pathogen *Candida albicans* ribosome. *Sci. Adv.* **2022**, *8* (21).

(8) Powers, K. T.; Stevenson-Jones, F.; Yadav, S. K. N.; Amthor, B.; Bufton, J. C.; Borucu, U.; Shen, D.; Becker, J. P.; Lavysh, D.; Hentze, M. W.; Kulozik, A. E.; Neu-Yilik, G.; Schaffitzel, C. Blasticidin S inhibits mammalian translation and enhances production of protein encoded by nonsense mRNA. *Nucleic Acids Res.* **2021**, *49* (13), 7665-7679.

(9) Garreau de Loubresse, N.; Prokhorova, I.; Holtkamp, W.; Rodnina, M. V.; Yusupova, G.; Yusupov, M. Structural basis for the inhibition of the eukaryotic ribosome. *Nature* **2014**, *513* (7519), 517-522.

(10) Svidritskiy, E.; Ling, C.; Ermolenko, D. N.; Korostelev, A. A. Blasticidin S inhibits translation by trapping deformed tRNA on the ribosome. *Proc. Natl. Acad. Sci. U.S.A.* **2013**, *110* (30), 12283-12288.

(11) Svidritskiy, E.; Korostelev, A. A. Mechanism of inhibition of translation termination by blasticidin S. *J. Mol. Biol.* **2018**, *430* (5), 591-593.

(12) Serrano, C. M.; Kanna-Reddy, H. R.; Eiler, D.; Koch, M.; Tresco, B. I. C.; Barrows, L. R.; VanderLinden, R. T.; Testa, C. A.; Sebahar, P. R.; Looper, R. E. Unifying the aminohexopyranose- and peptidyl-nucleoside antibiotics: Implications for antibiotic design. *Angew. Chem., Int. Ed.* **2020**, *59* (28), 11330-11333.

(13) The PyMOL Molecular Graphics System. Vol. Schrodinger, LLC.

(14) Abramson, J.; Adler, J.; Dunger, J.; Evans, R.; Green, T.; Pritzel, A.; Ronneberger, O.; Willmore, L.; Ballard, A. J.; Bambrick, J.; Bodenstein, S. W.; Evans, D. A.; Hung, C.-C.; O'Neill, M.; Reiman, D.; Tunyasuvunakool, K.; Wu, Z.; Žemgulytė, A.; Arvaniti, E.; Beattie, C.; Bertolli, O.; Bridgland, A.; Cherepanov, A.; Congreve, M.; Cowen-Rivers, A. I.; Cowie, A.; Figurnov, M.; Fuchs, F. B.; Gladman, H.; Jain, R.; Khan, Y. A.; Low, C. M. R.; Perlin, K.; Potapenko, A.; Savy, P.; Singh, S.; Stecula, A.; Thillaisundaram, A.; Tong, C.; Yakneen, S.; Zhong, E. D.; Zielinski, M.; Žídek, A.; Bapst, V.; Kohli, P.; Jaderberg, M.; Hassabis, D.; Jumper, J. M. Accurate structure prediction of biomolecular interactions with AlphaFold 3. *Nature* **2024**, *630* (8016), 493-500.

(15) Wiederstein, M.; Sippl, M. J. ProSA-web: interactive web service for the recognition of errors in three-dimensional structures of proteins. *Nucleic Acids Res.* **2007**, *35* (suppl_2), W407-W410.

(16) Sippl, M. J. Recognition of errors in three-dimensional structures of proteins. *Proteins: Struct., Funct., Bioinf.* **1993**, *17* (4), 355-362.

(17) Bowie, J. U.; Lüthy, R.; Eisenberg, D. A Method to Identify Protein Sequences That Fold into a Known Three-Dimensional Structure. *Science* **1991**, *253* (5016), 164-170.

(18) McNutt, A. T.; Francoeur, P.; Aggarwal, R.; Masuda, T.; Meli, R.; Ragoza, M.; Sunseri, J.; Koes, D. R. GNINA 1.0: molecular docking with deep learning. *J. Cheminform.* **2021**, *13* (1), 43.
